# Multiphasic modelling and computation of metastatic lung-cancer cell proliferation and atrophy in brain tissue based on experimental data

**DOI:** 10.1007/s10237-021-01535-4

**Published:** 2021-12-17

**Authors:** Wolfgang Ehlers, Markus Morrison (Rehm), Patrick Schröder, Daniela Stöhr, Arndt Wagner

**Affiliations:** 1 Institute of Applied Mechanics, Pfaffenwaldring 7, 70569 Stuttgart, Germany; 2 Institute of Cell Biology and Immunology, Allmandring 31, 70569 Stuttgart, Germany

**Keywords:** Brain tissue, Lung-cancer metastases, Theory of Porous Media, Drug delivery, Experimental data

## Abstract

Cancer is one of the most serious diseases for human beings, especially when metastases come into play. In the present article, the example of lung-cancer metastases in the brain is used to discuss the basic problem of cancer growth and atrophy as a result of both nutrients and medication. As the brain itself is a soft tissue that is saturated by blood and interstitial fluid, the biomechanical description of the problem is based on the Theory of Porous Media enhanced by the results of medication tests carried out in in-vitro experiments on cancer-cell cultures. Based on theoretical and experimental results, the consideration of proliferation, necrosis and apoptosis of metastatic cancer cells is included in the description by so-called mass-production terms added to the mass balances of the brain skeleton and the interstitial fluid. Furthermore, the mass interaction of nutrients and medical drugs between the solid and the interstitial fluid and its influence on proliferation, necrosis and apoptosis of cancer cells are considered. As a result, the overall model is appropriate for the description of brain tumour treatment combined with stress and deformation induced by cancer growth in the skull.

## Introduction

Lung cancer is one of the most frequent cancer types and additionally has the highest death probability for male patients, cf. the report of the Robert–Koch Institute (Kaatsch et al. 2014). One reason for the high mortality results from metastasation, as lung cancer is not only malignant but can also rapidly spread throughout the body. As a consequence, the metastatic spreading to remote organs and the affection of their functions is often tremendous. This motivates the current contribution to model the metastasation process in brain tissue and its related coupled interactions, such as a chemotherapeutic treatment.

Basically, cancer cells emerge from regular healthy cells, where an initial transformation towards a malfunctional cancer cell has occurred caused by specific mutations in the deoxyribonucleic acid (DNA). Usually, malfunctional cells undergo apoptosis, the programmed cell death initiated by specific triggering signals. In contrast to this, the apoptotic behaviour of most cancer cells changes in such a way that there is no reaction to apoptotic signals. Thus, the ability to overcome apoptosis is one of the hallmarks of cancer, cf. Hanahan and Weinberg (2000). In addition to the omitted cell death, cancer cells can acquire properties to successfully spawn towards different parts of the body. During the evolution of a tumour, cell division increases the number of cancer cells until a supply of the growing cell cluster with nutrients and oxygen is no longer possible by diffusive processes alone. Thereafter, the growth of cancer clusters or tumours, respectively, either stagnates or blood vessel growth (angiogenesis) towards the metastases is initiated, cf. Geiger and Peeper (2009). In the latter case, tumours are further supplied via the blood system, thus creating a progressive growth process, while the ability of non-restricted cell division increases the possibility for further mutations in cancer cells. Additionally, cancer cells disseminate from the tumour and migrate into the surrounding extracellular matrix (ECM) by either cutting through the ECM by mesenchymal motion or by crawling along the fibres (amoeboid motion), cf. Friedl and Wolf (2003). Invasive cancer cells can enter the blood vessel or the lymphatic system and migrate to distant organs forming metastases in their tissue, cf. Shaffrey et al. (2004).

Lung metastases mainly spread to bones, the adrenal gland, the liver and the brain, cf. Nguyen et al. (2009). The brain, as the organ under discussion, exhibits a highly complex structure formed by a network of cells embedded in an ECM with interstitial and cerebrospinal fluid and blood vessels. While the brain has essential functions such as memory and coordination, cf. Trepel (2017), a disturbance of brain tissue by metastases can dramatically reduce the performance of the brain and may result in life-threatening effects. However, a colonisation of the brain requires the passing of the so-called blood–brain barrier (BBB), a specific coating of the endothelial cells to block the migration of macromolecules and cells into the brain tissue, cf. Wilhelm et al. (2013). The BBB consists of very tight junctions between the endothelial cells that cancer cells may overwhelm by either breaking these junctions or by finding gaps between endothelial cells, cf. Chaffer and Weinberg (2011). However, the new tissue environment forces the infiltrated cancer cells to adapt to the changed conditions. As a result, cancer cells can be found in a dormant or in an active state. In this regard, the amount of cancer cells forming a metastasis, and thus being in an active state, is quite low in comparison with the overall amount of circling cancer cells, cf. Kienast et al. (2010).

**Fig. 1:**
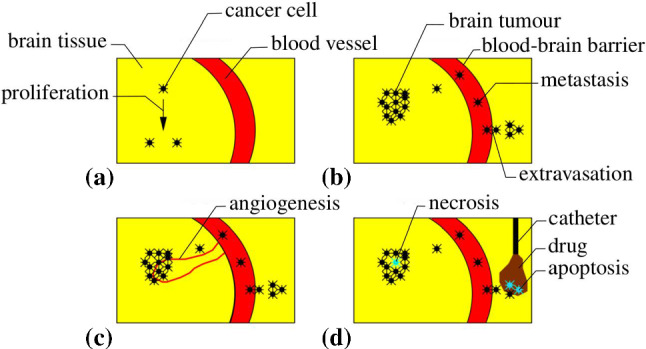
Metastasation. The hallmarks of the formation of metastases are **a** the unrestricted proliferation of cancer cells, **b** the invasion into the blood vessel system and the extravasation at a metastatic site, **c** the angiogenesis to overcome nutrient limitations and **d** the reduced apoptosis or necrosis in the cells, cf. Hanahan and Weinberg (2000)

In case of a metastasation, its initiation is in equivalence with the onset of tumourigenesis, the growth of primary tumours. Cancer cells start to divide, form initial micrometastases, induce angiogenesis and finally grow towards macrometastases, cf. Fig. 1. As a result, the growing metastases displace the surrounding tissue, thus increasing the local pressure and initiating deformation.

In terms of clinical detection processes, the metastases are at this stage large enough to be identified by the use of standard medical imaging devices. Consequently, this time characterises a typical starting point for a medical treatment. In a conventional medical treatment, a surgical removal of the tumour is conducted which is associated by a consecutive intravascular chemotherapy combined with radiotherapy. In cases where this treatment is not feasible, such as deeply in the brain situated tumours, an alternative treatment can be seen in the direct infusion of therapeutic agents via catheters into the brain tissue, cf. Bobo et al. (1994). During this surgery, the extravascular medication enters the brain through the skull, thus bypassing the BBB that typically would have hindered the passage of therapeutics with high molecular weights.

A continuum mechanical description accompanied by a numerical computation of cancer therapies must catch the basic features of the human body, especially of the organ under consideration. As the brain consists of a porous solid matrix composed of a network of cells embedded in the ECM, it must be understood as a typical porous medium that is percolated by interstitial fluid and blood both in separated compartments but interacting in capillary beds between arteries and veins. Following this, a biomechanical description is naturally based on the Theory of Porous Media (TPM), cf. Ehlers (2002, 2009). When cancer comes into play, the cancer cells are sticking to the porous solid, while nutrients, medical drugs and further components come along with the blood and the interstitial fluid.

In the literature, brain tissue is usually described as a very soft viscoelastic solid with heterogeneous microstructure and different properties in tension and compression, cf. Chatelin et al. (2010), Chaudhuri et al. (2015), Goriely et al. (2015), Zhao et al. (2018) and Zhu et al. (2019). As this is not sufficient for the complex structure of the brain, Hakim and Adams (1965), who have been working on the hydrocephalus problem, presented the early hypothesis that the effects occurring in the brain can only be described by the interplay of several brain tissue components. Decades later, their intuition has been followed by other scientists, such as Nagashima et al. (1987) or Smith and Humphrey (2007), who have been working on biphasic brain descriptions based on a porous brain tissue and interstitial fluid. Furthermore, Comellas et al. (2020) explored the porous and viscous responses of brain tissue material in a very recent publication. Apart from brain descriptions, a variety of further biomechanical problems has been based on multicomponent media, such as the general description of tissue growth by Ricken et al. (2007) or Ambrosi et al. (2011), the investigation of avascular and vascular tumour growth by Krause et al. (2012) and Hubbard and Byrne (2013) or the mass transport and deformation of multicomponent tumour growth, cf. Shelton (2011), Sciumè et al. (2013) and Faghihi et al. (2020).

The present contribution aims at modelling the main processes of lung cancer metastasation in the brain, particularly the processes of proliferation and apoptosis. Based on earlier work by Wagner (2014), Ehlers and Wagner (2015) and on a very recent article by Ehlers et al. (2021), a model of three components, namely porous brain tissue, interstitial fluid and blood, is set up in the framework of the TPM. This model is enhanced by experimental data that have been taken at the University of Stuttgart by the group of Professor Morrison at the Institute of Cell Biology and Immunology, cf. Stöhr (2018) and Stöhr et al. (2020). This allows for the identification of the most important model parameters for a reliable description of proliferation and apoptosis processes.

Proceeding from the basic model set-up tailored for the description of metastatic processes within brain tissue, the modelling approach is completed by a set of constitutive equations including a data-based parameter selection and its optimisation. As a result, one obtains a strongly coupled system of partial differential equations that has to be solved numerically. In terms of the present procedure, a finite-element-based implementation is derived within the solver Pandas.^1^ By this procedure, two-dimensional (2-d) boundary-value problems are computed that correspond to parameter optimisation studies focusing on proliferation and atrophy. Based on these studies, a combined numerical example is discussed exhibiting the process of metastasation from the initiation of cell infiltration over angiogenesis to the treatment. Therein, examples of different drug concentration levels and their effects on the overall cancer-cell mass are shown. Finally, an additional insight into a three-dimensional (3-d) problem is presented leading to future research directions.

The tensor calculus and notation used in this article are based on Ehlers (2018a).

## Continuum mechanics of brain tumours

### The TPM in brief

Based on the brain model by Ehlers and Wagner (2015), the continuum mechanical description of growth and atrophy of brain tumour metastases is mainly based on the mass and momentum balances of the model components. These are the healthy or the cancerous brain material, the blood and the interstitial fluid. As the blood primarily exists in arteries and veins, the fluids are assumed to basically exist in different compartments except of the capillary bed, where they interchange oxygen, nutrients and further ingredients. Thus, the present model $\varphi$ is composed of

1 $$\begin{aligned} \varphi = \displaystyle \bigcup _\alpha \,\varphi ^\alpha = \varphi ^S\cup \varphi ^B\cup \varphi ^I\quad {\mathrm{with}}\quad \alpha =\{S,\,B,\,I\}\,. \end{aligned}$$

Therein, $\varphi ^S$ stands for the porous brain solid, $\varphi ^B$ for the blood and $\varphi ^I$ for the interstitial fluid.

From the above composition, volume fractions $n^\alpha$ can formally be defined by the fraction of a volume element ${\mathrm{d}}v^\alpha$ of $\varphi ^\alpha$ over the total volume element ${\mathrm{d}}v$ of $\varphi$. Thus,

2 $$\begin{aligned} n^\alpha :=\frac{{\mathrm{d}}v^\alpha }{{\mathrm{d}}v} \quad \text{ with }\quad {{\mathrm{d}}v}=\sum _{\alpha =S,\,B,\,I}{{\mathrm{d}}v^\alpha }\,. \end{aligned}$$

This definition naturally includes the sum of all volume fractions to yield the so-called saturation condition

3 $$\begin{aligned} \sum \nolimits _\alpha n^\alpha =1\,. \end{aligned}$$

The governing set of balance equations is given by the mass and momentum balances of all basic constituents, solid, blood and interstitial fluid via

4 $$\begin{aligned}&(\rho ^\alpha )^\prime _\alpha \,+\,\rho ^\alpha \,\hbox {div}\, \overset{\prime }{{{\mathbf{x}}}}_\alpha = {\hat{\rho }}{}^\alpha \,,\nonumber \\&\rho ^\alpha \overset{\prime \prime }{\mathbf{x}}_\alpha = \hbox {div}\,{\mathbf{T}}^\alpha \,+\,\rho ^\alpha \,{\mathbf{g}}\,+\, {\hat{\mathbf{p}}}^\alpha \end{aligned}$$

with the constraining side conditions

5 $$\begin{aligned} \sum _\alpha {\hat{\rho }}^\alpha =0\quad \text{ and }\quad \sum _\alpha ({\hat{\mathbf{p}}}^\alpha +{\hat{\rho }}^\alpha \overset{\prime }{{\mathbf{x}}}_\alpha )=\mathbf{0}\,, \end{aligned}$$

cf. Ehlers (2002). In the above equations, $\rho ^\alpha =n^\alpha \rho ^{\alpha R}$ is the partial density of $\varphi ^\alpha$ given as the product of the intrinsic, effective or real density $\rho ^{\alpha R}$ and the volume fraction $n^\alpha$, ${\mathbf{T}}^\alpha$ is the partial Cauchy stress and ${\mathbf{g}}$ the gravitation vector. Furthermore, ${\hat{\rho }}{}^\alpha$ and $\hat{\mathbf{p}}^\alpha$ are the so-called density production and direct momentum production terms coupling the mass and momentum balances of solid and pore fluids. Constraints (5) result from the fact that the continuum mechanical system as a whole is a so-called closed system, while the individual components represent open systems, such that they can mutually interact with each other (Ehlers 2002).

Since the TPM provides a continuum mechanical view onto porous media problems, all terms have to be understood as local means representing the local averages of their microscopic counterparts. In particular, the density production represents an increase of partial density driven by chemo-physical processes like phase transformations or chemical reactions, while the direct momentum production exhibits the volumetric average of the local contact forces acting at the local interfaces (pore walls) between the solid and the pore fluids, on the one hand, and between the fluids, on the other hand. In addition to the above, $\text{ div }\,(\,\cdot \,)$ is the divergence operator corresponding to the gradient operator $\text{ grad }\,(\cdot )=\partial \,(\,\cdot \,)/\partial \,{\mathbf{x}}$ with $\mathbf{x}$ as the local position vector. Furthermore, $\overset{\prime }{{{\mathbf{x}}}}_\alpha$ and $\overset{\prime \prime }{{{\mathbf{x}}}}_\alpha$ are the local velocity and acceleration terms of $\varphi ^\alpha$, respectively, while $(\,\cdot \,)^\prime _\alpha$ is the material time derivative following the motion of $\varphi ^\alpha$.

In the present modelling process, all components are assumed to be materially incompressible meaning that their intrinsic densities $\rho ^{\alpha R}$ remain constant under isothermal conditions assuming a constant temperature of $37^\circ \,\hbox {C}$, such that energy balances can be ignored. For the solid skeleton, this basically means, although the intrinsic solid density remains constant, that the partial solid density $\rho ^S=n^S\rho ^{SR}$ can vary through variations of the solid volume fraction $n^S$ and the solid density production ${\hat{\rho }}^S$. Generally, materially incompressible constituents with constant $\rho ^{\alpha R}$ can be described by volume balances instead of mass balances obtained from (4)$_1$ through division by $\rho ^{\alpha R}$. Thus,

6 $$\begin{aligned} (n^\alpha )^\prime _\alpha +n^\alpha \text{ div }\,\overset{\prime }{\mathbf{x}}_\alpha ={\hat{n}}^\alpha \,, \quad \text{ where }\quad {\hat{n}}^\alpha =\frac{{\hat{\rho }}^\alpha }{\rho ^{\alpha R}}\,. \end{aligned}$$

### The tumour model in detail

**Fig. 2:**
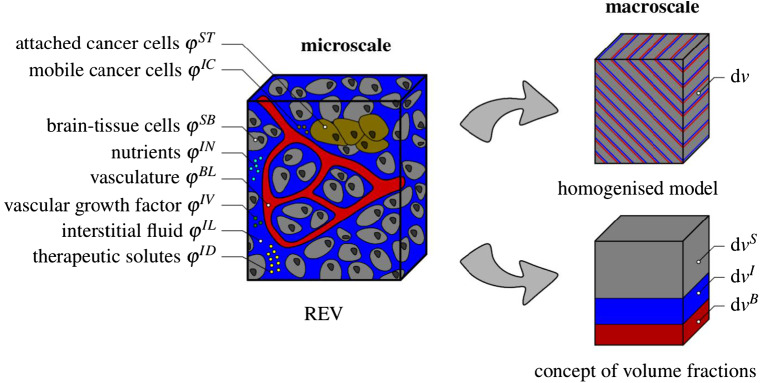
Representative elementary volume (REV) with exemplarily displayed microstructure of tumour-affected brain tissue and macroscopic multiphasic and multicomponental modelling approach

Describing tumour-related processes, we make the assumption that the solid skeleton is composed of healthy brain material $\varphi ^{SB}$ and tumour or metastatic cancer cells $\varphi ^{ST}$. It is furthermore assumed that the tumour can either be in an early avascular stage or, after angiogenesis, in a fully supplied stage. In the avascular stage, the tumour is not supplied by blood vessels and receives nutrients only from the surrounding tissue through the interstitial fluid. After angiogenesis, blood vessels have grown towards the tumour and interchange nutrients and further ingredients with the interstitial fluid and directly with the tumour. This complex situation is simplified by the assumption that the interstitial fluid remains the nutrient supplier, while the effect of the blood is taken into consideration through convenient boundary conditions of the respective numerical model. As a result of these assumptions, composition (1) of the model is specified by

7 $$\begin{aligned}&\varphi = \displaystyle \bigcup _\alpha \varphi ^\alpha = \varphi ^S \,\cup \, \varphi ^B \cup \, \varphi ^I\,,\nonumber \\&\text {where}\quad {\left\{ \begin{array}{ll} \varphi ^{S} = \varphi ^{SB}\,\cup \,\varphi ^{ST}\,,\\ \varphi ^B = \varphi ^{BL}\,,\\ \varphi ^I = \varphi ^{IL}\,\cup \,\varphi ^{IN}\,\cup \,\varphi ^{IC}\,\cup \,\varphi ^{IV}\,\cup \,\varphi ^{ID}\,, \end{array}\right. } \end{aligned}$$

also cf. Fig. 2. It is seen from (7) that by the use of the above assumption, the blood is only taken as the blood liquid $\varphi ^{BL}$, while the interstitial fluid $\varphi ^I$ is described as a mixture of multiple components $\varphi ^{I\gamma }$ with $\gamma =\{L,\,N,\,C,\,V,\,D\}$. Thus, $\varphi ^I$ consists of an interstitial fluid solvent $\varphi ^{IL}$ with nutrients $\varphi ^{IN}$, mobile cancer cells $\varphi ^{IC}$, vascular endothelial growth factors (VEGF) $\varphi ^{IV}$ and therapeutic drugs $\varphi ^{ID}$ as solutes. As a result, the nutrient exchange between the blood and the interstitial fluid is not taken into the model description, such that mass exchanges by mass production terms only occur between the interstitial fluid and the brain solid, such that ${{\hat{\rho }}}^B\equiv 0$.

Given $\varphi ^I=\cup _\gamma \,\varphi ^{I\gamma }$, the mass balance (4)$_1$ can be split into mass balances of the individual components of $\varphi ^I$ yielding

8 $$\begin{aligned}&(\rho ^{I\gamma })^\prime _{I\gamma }+\rho ^{I\gamma }\text{ div }\,\overset{\prime }{\mathbf{x}}_{I\gamma }={\hat{\rho }}{}^{I\gamma }\,,\quad \text{ where }\nonumber \\&\displaystyle \rho ^I=\sum _\gamma \rho ^{I\gamma }\,,\quad {\hat{\rho }}{}^I=\sum _\gamma {\hat{\rho }}{}^{I\gamma }\,,\quad \overset{\prime }{\mathbf{x}}_I=\frac{1}{\rho ^I}\sum _\gamma \rho ^{I\gamma }\overset{\prime }{\mathbf{x}}_{I\gamma } \end{aligned}$$

has been used (Ehlers 2009). Therein, $\overset{\prime }{\mathbf{x}}_{I\gamma }$ is the velocity of $\varphi ^{I\gamma }$, while $(\,\cdot \,)^\prime _{I\gamma }$ is the material time derivative following $\varphi ^{I\gamma }$. Furthermore, rebuilding the mass balance (4)$_1$ for $\varphi ^I$ by summing up (8)$_1$ over $\varphi ^{I\gamma }$ obviously yields

9 $$\begin{aligned} (\rho ^I)^\prime _I +\rho ^I\text{ div }\,\overset{\prime }{\mathbf{x}}_I={\hat{\rho }}{}^I\,, \quad \text{ where }\quad \mathbf{d}_{I\gamma }=\overset{\prime }{\mathbf{x}}_{I\gamma }-\overset{\prime }{\mathbf{x}}_I \end{aligned}$$

together with the identity $\sum _\gamma (\rho ^{I\gamma }\mathbf{d}_{I\gamma })={\mathbf{0}}$ has been used. In this setting, $\mathbf{d}_{I\gamma }$ defines the diffusion velocity of $\varphi ^{I\gamma }$ with respect to the motion of $\varphi ^I$.

The partial densities $\rho ^{I\gamma }$ of the interstitial fluid components $\varphi ^{I\gamma }$ can also be expressed as follows:

10 $$\begin{aligned}&\rho ^{I\gamma }=n^{I\gamma }\rho ^{I\gamma R}=s^{I\gamma }n^I\rho ^{I\gamma R}=n^I\rho ^{I\gamma }_I\nonumber \\&\text{ with }\quad \rho ^{I\gamma }_I:=s^{I\gamma }\rho ^{I\gamma R}\,. \end{aligned}$$

Therein, $n^{I\gamma }$ is the volume fraction of $\varphi ^{I\gamma }$ as $n^I=\sum _\gamma n^{I\gamma }$ is the volume fraction of the interstitial fluid. As a result, $s^{I\gamma }=n^{I\gamma }/n^I$ is the saturation of $\varphi ^{I\gamma }$ in $\varphi ^I$. While $\rho ^{I\gamma }$ defines the partial density of $\varphi ^{I\gamma }$ with respect to the volume of the overall aggregate, $\rho ^{I\gamma }_I$ with effective density $\rho ^{I\gamma R}$ characterises the partial density with respect to the volume of $\varphi ^I$.

As the partial densities $\rho ^{I\gamma }$ can be summed up to yield $\rho ^I$, cf. (8)$_2$, one analogously concludes to the effective density $\rho ^{IR}$ of the interstitial fluid reading

11 $$\begin{aligned} \rho ^{IR}=\sum _\gamma \rho ^{I\gamma }_I . \end{aligned}$$

Furthermore, $\rho ^{I\gamma }_I$ can be expressed by its molar concentration $c^{I\gamma }_m$ given in $\text{ mol/m}^3$ and its molar mass $M^{I\gamma }_m$ given in kg/mol via

12 $$\begin{aligned} \rho ^{I\gamma }_I= & {} c^{I\gamma }_m M^{I\gamma }_m\,, \quad \text{ such } \text{ that }\nonumber \\ \rho ^{IR}= & {} \sum _\gamma c^{I\gamma }_m M^{I\gamma }_m\quad \text{ and }\quad \rho ^I=n^I\sum _\gamma c^{I\gamma }_m M^{I\gamma }_m\,. \end{aligned}$$

cf. Ehlers (2009). As a result of (11) and (12), the mass balance (8)$_1$ of the species $\varphi ^{I\gamma }$ reduces to the concentration balance

13 $$\begin{aligned} (n^I c^{I\gamma }_m)^\prime _{I\gamma }+n^I c^{I\gamma }_m\text{ div }\,\overset{\prime }{\mathbf{x}}_{I\gamma }=\frac{{\hat{\rho }}{}^{I\gamma }}{M^{I\gamma }_m} \end{aligned}$$

through division by the constant molar masses $M^{I\gamma }_m$.

As the partial densities $\rho ^{I\delta }_I$ of the solutes $\varphi ^{I\delta }$ with $\delta =\{N,\,C,\,V,\,D\}\,$ included in (12)$_1$ are generally negligible with respect to the partial density $\rho ^{IL}_I$ of the interstitial fluid solvents $\varphi ^{IL}$, it is justified to use

14 $$\begin{aligned} \rho ^{IR}\,\approx \,\rho ^{IL}_I=c^{IL}_m M^{IL}_m\,, \end{aligned}$$

thus also supporting the assumption of constant $\rho ^{IR}$ at constant temperature for the materially incompressible interstitial fluid components.

Following the assumption of materially incompressible constituents $\varphi ^\alpha$, the mass balances reduce to volume balances, such that (6) results in

15 $$\begin{aligned}&(n^S)^\prime _S+n^S\text{ div }\,({\mathbf{u}}_S)^\prime _S={\hat{n}}^S\,,\nonumber \\&(n^B)^\prime _S+\text{ div }\,(n^B\mathbf{w}_B)+n^B\text{ div }\,\overset{\prime }{\mathbf{x}}_B=0\,,\nonumber \\&(n^I)^\prime _S+\text{ div }\,(n^I\mathbf{w}_I)+n^I\text{ div }\,\overset{\prime }{\mathbf{x}}_I={\hat{n}}^I\,, \end{aligned}$$

where ${\mathbf{w}}_\beta =\overset{\prime }{\mathbf{x}}_\beta -\overset{\prime }{\mathbf{x}}_S$ defines the seepage velocity of $\varphi ^\beta$, while ${\mathbf{u}}_S={\mathbf{x}}-{\mathbf{x}}_0$ characterises the solid displacement vector with ${\mathbf{x}}_0$ as the solid location vector in the reference configuration at time $t=t_0$, thus including $\overset{\prime }{\mathbf{x}}_S=({\mathbf{u}}_S)^\prime _S$. Note that the shape of the fluid balances given in (15)$_2$ is necessary for the numerical treatment of porous media problems, since the solid material is treated by a Lagrangian description on the basis of the solid displacement vector ${\mathbf{u}}_S$, while the pore fluids $\varphi ^\beta$ are described in the framework of a modified Eulerian description relatively to the deforming skeleton by the their seepage velocities ${\mathbf{w}}_\beta$.

Furthermore, the volume productions ${\hat{n}}^S$ and ${\hat{n}}^I$ are coupled via the mass balance side condition (5)$_1$, such that

16 $$\begin{aligned} {{\hat{\rho }}}^S+{{\hat{\rho }}}^I=0 \quad \text{ yielding }\quad {\hat{n}}^S=-\frac{\rho ^{IR}}{\rho ^{SR}}\,{\hat{n}}^I\,, \end{aligned}$$

where (6)$_2$ has been used. Finally, the solid volume balance (15)$_1$ can be integrated analytically to yield

17 $$\begin{aligned} n^S= & {} \displaystyle \,n^S_{0}\,\exp \,\Big (\, \int ^t_0 \frac{{\hat{n}}^S}{n^S} \,{\mathrm{d}}{\tilde{t}} \,\Big )\,(\det \mathbf{F}_S)^{-1}\,\nonumber \\=: & {} \,n^S_g\,(\det {\mathbf{F}}_S)^{-1}\,, \end{aligned}$$

compare "Appendix (a)". In the above equation, $n^S_g$ can be interpreted as the growth-configuration-based solid volume fraction that is related to the current volume fraction $n^S$ through the inverse determinant of the solid’s deformation gradient ${\mathbf{F}}_S={\mathbf{I}}+\text{ Grad}_S\,{\mathbf{u}}_S$ with ${\mathbf{I}}$ as the second-order identity tensor and $\text{ Grad}_S=\partial (\,\cdot \,)/\partial {\mathbf{x}}_0$. Note in passing that $n^S_g$ reduces to the standard reference solid volume fraction $n^S_0$ at $t_0$, whenever the integral in (17) vanishes, which is trivially fulfilled in case of ${\hat{n}}^S\equiv 0$. Finally, it should be noted that the notion “growth” does not only include proliferation but also atrophy by necrosis and/or apoptosis.

As the solid skeleton is built from brain and metastatic material, it is assumed that

18 $$\begin{aligned}&n^S=n^{SB}+n^{ST} \quad \text{ with }\quad \left\{ \begin{array}{l} n^{SB}(t_0)=n^S_0\,,\\ n^{ST}(t_0)=0 \end{array}\right. \nonumber \\&\text{ and }\quad {\hat{n}}^S= {\hat{n}}^{ST}\,. \end{aligned}$$

These equations underline the fact that at $t=t_0$, the solid volume fraction only contains brain material, while metastatic material only comes into play through the existence of $n^{ST}$ after clustering cancer cells have proliferated, such that the number of cells leap over a certain threshold, thus initialising a micrometastasis growing with ${\hat{n}}^{ST}$. Before this happens, cancer cells spread into the brain tissue, while their volume fraction in the brain is not measurable.

Nevertheless, it seems necessary to add the following remark to the above volume balances. Although the components of the overall brain material have been taken as being materially incompressible expressed through constant values of $\rho ^{\alpha R}$, there is a mass transfer between the brain solid and the interstitial fluid, meaning that cancer cells and further fluid species can be added to the brain skeleton. However, assuming that cancer cells have the same effective density as the basic brain material and that the masses of the species are negligible, the assumption $\rho ^{SR}=\text{ const. }$ is still justified, while the proliferation process is taken into consideration by an intake of brain volume through ${\hat{n}}^S$. Concerning the effective densities $\rho ^{\beta R}$ of the interstitial fluid components $\varphi ^I$, it is seen from (7)$_3$ and (12)$_2$ that

19 $$\begin{aligned} \rho ^{IR}=c^{IL}_m M^{IL}_m+\sum _\delta c^{I\delta }_m M^{I\delta }_m\,. \end{aligned}$$

When cancer cells spread into the brain tissue, this process has to be described by concentrations rather than by volume fractions. Following this, the necessary concentration balances of the solutes $\varphi ^{I\delta }$ can be obtained with the aid of (13) and (15) together with the saturation condition (3):

20 $$\begin{aligned}&n^I(c^{I\delta }_m ){}^{\prime }_S+\text{ div }\,(n^Ic^{I\delta }_m\mathbf{w}_{I\delta })+c^{I\delta }_m\,\text{ div }\,(n^{B}{\mathbf{w}}_B)+\,c^{I\delta }_m\text{ div }\,({\mathbf{u}}_S){}^\prime _S\nonumber \\ \displaystyle&=c^{I\delta } _m\Big (\frac{{\hat{\rho }}^S}{\rho ^{SR}}+\frac{{\hat{\rho }}^{I\delta }}{\rho ^{I\delta }_I}\Big )\,. \end{aligned}$$

Therein, and in addition to the standard seepage velocities $\mathbf{w}_\beta$, the relative velocities ${\mathbf{w}}_{I\delta }$ are defined through ${\mathbf{w}}_{I\delta }=\overset{\prime }{\mathbf{x}}_{I\delta }-\overset{\prime }{\mathbf{x}}_S$. As these velocities formally also have the character of a seepage velocity, they can alternatively be described by the diffusion velocity of $\varphi ^{I\delta }$ and the seepage velocity of $\varphi ^I$, such that ${\mathbf{w}}_{I\delta }={\mathbf{d}}_{I\delta }+{\mathbf{w}}_I$ obviously exhibits the diffusion of $\varphi ^{I\gamma }$ in the moving frame of $\varphi ^I$.

Apart from the complex matter of volume and concentration balances, the model equations are only complete after having added momentum balances. In the present case, it is sufficient to proceed with the momentum balance of the overall model under quasi-static conditions. Following this, the momentum balances (4)$_2$ of solid, blood and interstitial fluid are added to yield

21 $$\begin{aligned} {\mathbf{0}} = \text{ div } {\sum _{\alpha }{\mathbf{T}}^{\alpha }}\,+\,\rho \,\mathbf{g}\,+\,\sum _{\alpha }\hat{{\mathbf{p}}}^\alpha \,, \end{aligned}$$

where inertia terms have been neglected. Furthermore, the overall density $\rho$ or the so-called mixture density is defined as

22 $$\begin{aligned} \rho = n^S\rho ^{SR}+\sum _\beta n^\beta \rho ^{\beta R}, \end{aligned}$$

with $$\rho ^{\beta R}$$ from (11).

The equation system described above is only appropriate to solve tumour-relevant problems, once it has been closed by convenient constitutive equations for the Cauchy stresses ${\mathbf{T}}^\alpha$, the direct momentum productions $\hat{\mathbf{p}}^\alpha$ and the volume productions ${\hat{n}}^\alpha$ or the density productions ${\hat{\rho }}^\alpha$, respectively. In contrast to the Cauchy stresses that have to be found for all constituents, direct momentum productions $\hat{\mathbf{p}}^\alpha$ and the density productions ${\hat{\rho }}^\alpha$ are constrained through (5) and ${{\hat{\rho }}}^B\equiv 0$, such that

23 $$\begin{aligned} {\hat{\rho }}^S= & {} -\sum _\beta {\hat{\rho }}^\beta =-{{\hat{\rho }}}^I\,,\nonumber \\ \hat{\mathbf{p}}^S= & {} -\sum _\beta (\,{\hat{\mathbf{p}}}^\beta +{\hat{\rho }}^\beta {\mathbf{w}}_\beta )=-(\,\hat{\mathbf{p}}^B+{\hat{\mathbf{p}}}^I+{{\hat{\rho }}}^I{\mathbf{w}}_I\,)\,. \end{aligned}$$

It should furthermore be noted that the solid volume fraction $n^S$ can be computed from its initial value $n^S_0$, the solid deformation gradient ${\mathbf{F}}_S$ and the constitutive equation for ${\hat{n}}^S$, cf. (17). As a result, the porosity $n^P=n^B+n^I$ is obtained from the saturation condition (3) through $n^P=1-n^S$. Thus, only the porosity and not the blood and interstitial fluid saturations

24 $$\begin{aligned} s^\beta =\frac{n^\beta }{n^P} \quad \text{ with }\quad \sum _\beta s^\beta =1 \end{aligned}$$

can be obtained from the relations given so far, such that either $s^B$ or $s^I$ has to be found by an additional constitutive equation.

## Constitutive equations

The brain-tumour model described above has to be closed by a set of convenient constitutive equations that includes, on the one hand, the full complexity of the model and is, on the other hand, as simple as possible. These two seemingly contradictory goals can be met coincidentally with the following additional assumptions: During the avascular growth of a brain metastasis, nutrients and further ingredients reach the cell cluster by diffusion through the interstitial fluid. As a result, the interstitial fluid has to be modelled as a mixture of liquid solvent and various solutes governed by (7). In this state of growth, the blood does not play a dominant role as carrier of nutrients. However, after angiogenesis, the blood takes over as the main supplier of the growing tumour. To avoid an overburdening of the model with two concurring fluid mixtures in the overall pore space, we have refrained from dealing with an additional blood mixture, thus treating the blood through convenient boundary conditions.

With this in mind, the model $\varphi$ consists of a porous solid $\varphi ^S$, the blood $\varphi ^B$ and the interstitial fluid mixture $\varphi ^I$ with $\cup _\gamma \varphi ^{I\gamma }$ ingredients that split into $\varphi ^{IL}$ as the solvent and $\cup _\delta \varphi ^{I\delta }$ as the solutes.

### Thermodynamical restrictions

As the constitutive equations have to fulfil the entropy inequality of the overall model, one has to take a view upon the Clausius–Planck inequality basically yielding

25 $$\begin{aligned} \sum _\alpha \big [\,{\mathbf{T}}^\alpha \cdot \mathbf{L}_\alpha -\rho ^\alpha (\psi ^\alpha )^\prime _\alpha -{\hat{\mathbf{p}}}^\alpha \cdot \overset{\prime }{\mathbf{x}}_\alpha -{\hat{\rho }}^\alpha (\psi ^\alpha +\textstyle {\frac{1}{2}}\overset{\prime }{\mathbf{x}}_\alpha \cdot \overset{\prime }{\mathbf{x}}_\alpha )\big ]\ge 0\,, \end{aligned}$$

cf. Eq. (106) of Ehlers (2002) for a constant temperature $\theta$. Considering the side conditions (23) and the saturation constraint

26 $$\begin{aligned} 0= & {} \varLambda \big [\, n^S\text{ div }\,\overset{\prime }{\mathbf{x}}_S-\frac{{\hat{\rho }}^S}{\rho ^{SR}}+n^B\text{ div }\,\overset{\prime }{\mathbf{x}}_B+\text{ grad }\,n^B\cdot {{\mathbf{w}}_B}\nonumber \\&+\,\sum _\gamma (n^{I\gamma }\text{ div }\,\overset{\prime }{\mathbf{x}}_{I\gamma }-\frac{{\hat{\rho }}^{I\gamma }}{\rho ^{I\gamma R}}+\text{ grad }\,n^{I\gamma }\cdot {\mathbf{w}_{I\gamma }})\,\big ] \end{aligned}$$

resulting from the time derivative $(\,\cdot \,)^\prime _S$ of the saturation condition (3) multiplied by a Lagrange multiplier $\varLambda$, the entropy inequality for the present tumour model reads

27 $$\begin{aligned}&{[}\,{\mathbf{T}}^S+n^S(\varLambda -\varPsi ^I_I)\,{\mathbf{I}}\,]\cdot \mathbf{L}_S-\rho ^S(\psi ^S)^\prime _S\nonumber \\&\quad -\,{\hat{\rho }}^S\Big (\psi ^{S}-\textstyle {\frac{1}{2}}\overset{\prime }{\mathbf{x}}_S\cdot \overset{\prime }{\mathbf{x}}_S+\displaystyle \frac{\varLambda -\varPsi ^I_I}{\rho ^{SR}}\Big )\nonumber \\&\quad +\,[\,{\mathbf{T}}^B+n^B(\varLambda -\varPsi ^I_I)\,{\mathbf{I}}\,]\cdot \mathbf{L}_B-\rho ^S(\psi ^B)^\prime _B\nonumber \\[1ex]&\quad -\,[\,\hat{\mathbf{p}}^B-(\varLambda -\varPsi ^I_I)\,\text{ grad }\,n^B\,]\,\cdot {\mathbf{w}}_B\nonumber \\[1ex]&\quad +\,\sum _\gamma \,\{[\,\mathbf{T}^{I\gamma }+n^I(s^{I\gamma }\varLambda -\varPsi ^{I\gamma }_I\,)\,\mathbf{I}\,]\,\cdot {\mathbf{L}}_{I\gamma }-n^I(\varPsi ^{I\gamma }_I)^\prime _{I\gamma }\nonumber \\&\quad -\,[\,\hat{\mathbf{p}}^{I\gamma }-(s^{I\gamma }\varLambda -\varPsi ^{I\gamma }_I) \,\text{ grad }\,n^I-n^I\varLambda \text{ grad }\,n^{I\gamma }\,]\,\cdot \mathbf{w}_{I\gamma }\nonumber \\&\quad -\,{\hat{\rho }}^{I\gamma }(\psi ^{I\gamma }+\textstyle \frac{1}{2}\overset{\prime }{\mathbf{x}}_{I\gamma }\cdot \overset{\prime }{\mathbf{x}}_{I\gamma }-\overset{\prime }{\mathbf{x}}_{I\gamma }\cdot \overset{\prime }{\mathbf{x}}_S+\displaystyle \frac{s^{I\gamma }\varLambda -\varPsi ^{I\gamma }_I}{\rho ^{I\gamma }_I})\,\}\ge 0\,. \end{aligned}$$

Obtaining (27) from (25), not only the saturation constraint has been used but also the fact that the free energy of a mixture is usually given per unit mixture volume through $\varPsi ^I_I$ and not per unit mixture mass through $\psi ^I$. Thus, the mass-specific free energy of the interstitial fluid mixture is expressed as

28 $$\begin{aligned}&\rho ^I\psi ^I=n^I\rho ^{IR}\psi ^I=:n^I\varPsi ^I_I=n^I\sum _\gamma \varPsi ^{I\gamma }_I\nonumber \\&\text{ where }~~\varPsi ^{I\gamma }_I=\rho ^{I\gamma }_I\psi ^{I\gamma }~~\text{ and } ~~\rho ^{IR}=\sum _\gamma \rho ^{I\gamma }_I\,, \end{aligned}$$

also cf. (11). Furthermore, note that $s^{I\gamma }=n^{I\gamma }/n^I$ is usually also addressed as a saturation as $s^\beta$ in (24). Here, it typically acts as a volume fraction of the species $\varphi ^{I\gamma }$ in the mixture $\varphi ^I$ with density $\rho ^{IR}$ and can thus be computed via

29 $$\begin{aligned}&\rho ^{I\gamma }= \left\{ \begin{array}{c} n^{I\gamma }\rho ^{I\gamma R}=n^I(s^{I\gamma }\rho ^{I\gamma R})\\ n^I\rho ^{I\gamma }_I \end{array}\right\} \displaystyle \rightarrow ~s^{I\gamma }=\frac{n^{I\gamma }}{n^I}=\frac{\rho ^{I\gamma }_I}{\rho ^{I\gamma R}}\nonumber \\&\text{ with }\quad \sum _\gamma \,s^{I\gamma }=1\,. \end{aligned}$$

Therein, $\rho ^{I\gamma }_I$ is defined through (12) as the partial mixture density of $\varphi ^{I\gamma }$ with effective density $\rho ^{I\gamma R}$.

Based on the principle of phase separation, cf. Ehlers (1989), stating that the free energies of immiscible components of the overall aggregate, as on the microscale, do only depend on their own constitutive variables, the solid free energy $\psi ^S$ of an isotropic elastic solid only depends on the solid deformation through the deformation gradient ${\mathbf{F}}_S$. In case that both incompressible fluids would not only be immiscible but also inert without any ingredients, the free energy of the interstitial fluid $\psi ^I$ would be constant and the free energy $\psi ^B$ of the blood would be a function of the saturation $s^B$ (Ehlers 2009). However, as the interstitial fluid is a fluid mixture, the volume-specific free energy $\varPsi ^I_I$ is taken into account instead of the mass-specific energy $\psi ^I$. Following this, $\varPsi ^I_I=\sum _\gamma \varPsi ^{I\gamma }_I$ is a function of the concentrations $c^{I\gamma }_m$ of the species through

30 $$\begin{aligned} \varPsi ^{I\gamma }_I=\rho ^{I\gamma }_I\psi ^I=c^{I\gamma }_mM^{I\gamma }_m\psi ^{I\gamma }\,, \end{aligned}$$

where $c^{I\gamma }_m$ is the only variable. Thus,

31 $$\begin{aligned} \psi ^S= & {} \psi ^S({\mathbf{F}}_S)\,,\quad \psi ^B=\psi ^B(s^B)\quad \text{ and }\nonumber \\ \varPsi ^I_I= & {} \sum _\gamma \varPsi ^{I\gamma }_I( c^{I\gamma }_m)\,. \end{aligned}$$

Following (31), the time derivatives of the free energies yield with the aid of (6), (8), (11) and (12)

32 $$\begin{aligned} \rho ^S(\psi ^S)^\prime _S= & {} \rho ^S\frac{\partial \psi ^S}{\partial \mathbf{F}_S}\,\cdot \,(\mathbf{F}_S)^\prime _S=\rho ^S\frac{\partial \psi ^S}{\partial {\mathbf{F}}_S}\mathbf{F}^T_S\,\cdot \,{\mathbf{L}}_S\,,\nonumber \\ \rho ^B(\psi ^B)^\prime _B= & {} \rho ^B\frac{\partial \psi ^B}{\partial s^B}(s^B)^\prime _B\nonumber \\= & {} -(s^B)^2\rho ^{BR}\frac{\partial \psi ^B}{\partial s^B}\,\bigg (n^F\text{ div }\,\overset{\prime }{\mathbf{x}}_B\nonumber \\&+\,n^S\text{ div }\,\overset{\prime }{\mathbf{x}}_S-\text{ grad }\,n^S\,\cdot \,\mathbf{w}_B-\frac{{\hat{\rho }}^S}{\rho ^{SR}}\,\bigg )\,,\nonumber \\ n^I(\varPsi ^{I\gamma }_I)^\prime _{I\gamma }= & {} n^I\frac{\partial \varPsi ^{I\gamma }_I}{\partial c^{I\gamma }_m}(c^{I\gamma }_m)^\prime _{I\gamma }\nonumber \\= & {} -c^{I\gamma }_m\frac{\partial \varPsi ^{I\gamma }_I}{\partial c^{I\gamma }_m}\bigg (n^S\text{ div }\,\overset{\prime }{\mathbf{x}}_S-\frac{{\hat{\rho }}^S}{\rho ^{SR}}\nonumber \\&+\,n^B\text{ div }\,\overset{\prime }{\mathbf{x}}_B\,+\text{ grad }\,n^B\,\cdot \,{\mathbf{w}}_B\nonumber \\&+\,n^I\text{ div }\,\overset{\prime }{\mathbf{x}}_{I\gamma }+\text{ grad }\,n^I\,\cdot \,\mathbf{w}_{I\gamma }-\frac{{\hat{\rho }}^{I\gamma }}{\rho ^{I\gamma }_I}\,\bigg )\,. \end{aligned}$$

Inserting these results in (27) leads to the final version of the entropy inequality:

33 $$\begin{aligned}&\Big [\,{\mathbf{T}}^S+n^S\Big (\,\varLambda -\varPsi ^I_I+\sum _\gamma c^{I\gamma }_m\frac{\partial \varPsi ^{I\gamma }_I}{\partial c^{I\gamma }_m}+(s^B)^2\rho ^{BR}\frac{\partial \psi ^B}{\partial s^B}\,\Big )\,{\mathbf{I}}\nonumber \\&\quad -\,\rho ^S\frac{\partial \psi ^S}{\partial {\mathbf{F}}_S}\mathbf{F}^T_S\Big ]\,\cdot {\mathbf{L}}_S -{\hat{\rho }}^S\Big [\psi ^{S}-\textstyle {\frac{1}{2}}\overset{\prime }{\mathbf{x}}_S\cdot \overset{\prime }{\mathbf{x}}_S\nonumber \\&\quad +\,\displaystyle \frac{1}{\rho ^{SR}}\Big (\varLambda -\varPsi ^I_I+\sum _\gamma c^{I\gamma }_m\frac{\partial \varPsi ^{I\gamma }_I}{\partial c^{I\gamma }_m}+(s^B)^2\rho ^{BR}\frac{\partial \psi ^B}{\partial s^B}\Big )\,\Big ]\nonumber \\&\quad +\,\Big [\,{\mathbf{T}}^B+n^B\Big (\,\varLambda -\varPsi ^I_I+\sum _\gamma c^{I\gamma }_m\frac{\partial \varPsi ^{I\gamma }_I}{\partial c^{I\gamma }_m}+s^B\rho ^{BR}\frac{\partial \psi ^B}{\partial s^B}\,\Big )\,{\mathbf{I}}\,\Big ]\,\cdot {\mathbf{L}}_B\nonumber \\&\quad -\,\Big [\,\hat{\mathbf{p}}^B-\Big (\varLambda -\varPsi ^I_I+\sum _\gamma c^{I\gamma }_m\frac{\partial \varPsi ^{I\gamma }_I}{\partial c^{I\gamma }_m}\Big )\,\text{ grad }\,n^B\nonumber \\&\quad +\,(s^B)^2\rho ^{BR}\frac{\partial \psi ^B}{\partial s^B}\,\text{ grad }\,n^S\,\Big ]\,\cdot {\mathbf{w}}_B\nonumber \\&\quad +\,\sum _\gamma \,\Big \{\Big [\,\mathbf{T}^{I\gamma }+n^I\Big (s^{I\gamma }\varLambda -\varPsi ^{I\gamma }_I +c^{I\gamma }_m\frac{\partial \varPsi ^{I\gamma }_I}{\partial c^{I\gamma }_m}\,\Big )\,{\mathbf{I}}\,\Big ]\,\cdot {\mathbf{L}}_{I\gamma }\nonumber \\&\quad -\,\Big [\,\hat{\mathbf{p}}^{I\gamma }-\Big (s^{I\gamma }\varLambda -\varPsi ^{I\gamma }_I+c^{I\gamma }_m\frac{\partial \varPsi ^{I\gamma }_I}{\partial c^{I\gamma }_m}\Big )\,\text{ grad }\,n^I\nonumber \\&\quad -\,n^I\varLambda \text{ grad }\,s^{I\gamma }\,\Big ]\,\cdot \mathbf{w}_{I\gamma } -{\hat{\rho }}^{I\gamma }\Big [\psi ^{I\gamma } +\textstyle \frac{1}{2}\overset{\prime }{\mathbf{x}}_{I\gamma }\cdot \overset{\prime }{\mathbf{x}}_{I\gamma }-\overset{\prime }{\mathbf{x}}_{I\gamma }\cdot \overset{\prime }{\mathbf{x}}_S\nonumber \\&\quad +\,\frac{1}{\rho ^{I\gamma }_I}\Big (s^{I\gamma }\varLambda -\varPsi ^{I\gamma }_I+c^{I\gamma }_m\frac{\partial \varPsi ^{I\gamma }_I}{\partial c^{I\gamma }_m}\Big )\,\Big ]\,\Big \}\ge 0\,. \end{aligned}$$

Based on (33), the exploitation of the entropy principle yields equilibrium and non-equilibrium solutions that can be obtained by the use of the standard Coleman–Noll procedure (Coleman and Noll 1963), also compare Hassanizadeh (1986), Schreyer Bennethum et al. (2000) and Araujo and McElwain (2005). Before carrying out these results, let us have a look at the mixture species $\varphi ^{I\gamma }$ and the classical relations between free energies $\varPsi ^{I\gamma }_I$, molar (mole-specific) chemical potentials $\mu ^{I\gamma }_m$ and osmotic pressures $\pi ^{I\gamma }$ (Ehlers 2009):

34 $$\begin{aligned} \mu ^{I\gamma }_m= & {} \frac{\partial \varPsi ^{I\gamma }_I}{\partial c^{I\gamma }_m} \quad \text{ and }\quad \pi ^{I\gamma }=c^{I\gamma }_m\mu ^{I\gamma }_m-\varPsi ^{I\gamma }_I \quad \text{ yielding }\nonumber \\ \pi ^{I\gamma }= & {} c^{I\gamma }_m\frac{\partial \varPsi ^{I\gamma }_I}{\partial c^{I\gamma }_m}-\varPsi ^{I\gamma }_I\,. \end{aligned}$$

*Thermodynamic equilibrium*:  Considering (33) in thermodynamic equilibrium, the terms in brackets are assumed to be independent of their multipliers, the velocity gradients and seepage velocities. Furthermore, mass productions are considered, at the first glance, as non-equilibrium terms.

Given the above, one concludes from line 7 of (33), where in case of thermodynamic equilibrium the term in brackets has to vanish for arbitrary $\mathbf{L}_{I\gamma }$, that

35 $$\begin{aligned} {\mathbf{T}}^{I\gamma }= & {} -n^I\Big (s^{I\gamma }\varLambda -\varPsi ^{I\gamma }_I +c^{I\gamma }_m\frac{\partial \varPsi ^{I\gamma }_I}{\partial c^{I\gamma }_m}\,\Big )\,{\mathbf{I}}\nonumber \\= & {} -n^I\Big (s^{I\gamma }\varLambda +\pi ^{I\gamma }\Big )\,{\mathbf{I}} \end{aligned}$$

and, as a result,

36 $$\begin{aligned}&{\mathbf{T}}^I=\sum _\gamma \mathbf{T}^{I\gamma }=-n^I\sum _\gamma (s^{I\gamma }\varLambda +\pi ^{I\gamma }\,)\,\mathbf{I}\, =-n^I(\varLambda +\pi ^I)\,{\mathbf{I}}\,,\nonumber \\&\text{ where }\quad \pi ^I=\sum _\gamma \pi ^{I\gamma } \quad \text{ and }\quad \sum _\gamma \,s^{I\gamma }=1 \end{aligned}$$

together with (34) have been used, also cf. (29). As the pressure governing ${\mathbf{T}}^I$ has to equal the partial liquid pressure $p^I=n^I p^{IR}$, one easily concludes to

37 $$\begin{aligned}&{\mathbf{T}}^I=-n^I(\varLambda +\pi ^I)\,{\mathbf{I}}\,=:-n^I p^{IR}\,{\mathbf{I}}\,,\nonumber \\&\text{ and } \text{ thus }\quad \varLambda =p^{IR}-\pi ^I\,=:p^{IR}_m. \end{aligned}$$

As $p^{IR}$ is the effective interstitial fluid pressure, it is easily concluded from (37) that $p^{IR}$ splits into a mechanical part, $p^{IR}_m$, thus also defining the Lagrangian multiplier $\varLambda$, and a chemical contribution, the osmotic pressure $\pi ^I$.

With this in mind, the equilibrium solution for ${\mathbf{T}}^B$ yields

38 $$\begin{aligned} {\mathbf{T}}^B= & {} -n^B\Big (\,\varLambda -\varPsi ^I_I+\sum _\gamma c^{I\gamma }_m\frac{\partial \varPsi ^{I\gamma }_I}{\partial c^{I\gamma }_m}+s^B\rho ^{BR}\frac{\partial \psi ^B}{\partial s^B}\,\Big )\,{\mathbf{I}}\nonumber \\= & {} -n^B\Big (\varLambda +\pi ^I+s^B\rho ^{BR}\frac{\partial \psi ^B}{\partial s^B}\,\Big )\,{\mathbf{I}}\,=:-n^B p^{BR}\,{\mathbf{I}}\,, \end{aligned}$$

such that

39 $$\begin{aligned} p^{{{{\mathrm{dif}}}}}=p^{BR}-p^{IR}=s^B\rho ^{BR}\frac{\partial \psi ^B}{\partial s^B}, \end{aligned}$$

with $p^{{{\mathrm{dif}}}}$ as the difference pressure between the blood and the interstitial fluid. In addition to the fluid stresses, the solid stress reads

40 $$\begin{aligned}&{\mathbf{T}}^S+n^S\Big (\,\varLambda -\varPsi ^I_I+\sum _\gamma c^{I\gamma }_m\frac{\partial \varPsi ^{I\gamma }_I}{\partial c^{I\gamma }_m}+(s^B)^2\rho ^{BR}\frac{\partial \psi ^B}{\partial s^B}\,\Big )\,{\mathbf{I}}\nonumber \\&\quad ={\mathbf{T}}^S+n^S\,\underbrace{[\,(1-s^B)\,p^{IR}+s^B p^{BR}\,]}_{p^{FR}}\,\mathbf{I}=\rho ^S\frac{\partial \psi ^S}{\partial {\mathbf{F}}_S}{\mathbf{F}}^T_S\,, \end{aligned}$$

where (35)–(38) have been used. Note in passing that $p^{FR}$ is the effective overall fluid pressure, also known as pore pressure, and that the above relation defining $p^{FR}$ recovers Dalton’s law (Dalton 1802). It should furthermore be noted that ${\mathbf{T}}^S+n^S p^{FR}\,{\mathbf{I}}$ is the so-called effective stress ${\mathbf{T}}^S_{{\mathrm{eff}}}$ defined as that part of the solid stress that governs the stress–strain relation of the solid (Ehlers 2018b), such that the solid stress ${\mathbf{T}}^S$ reads

41 $$\begin{aligned} {\mathbf{T}}^S=-n^S p^{FR}\,{\mathbf{I}}\,+{\mathbf{T}}^S_{{\mathrm{eff}}} \quad \text{ with }\quad {\mathbf{T}}^S_{\mathrm{eff}}=\rho ^S\frac{\partial \psi ^S}{\partial {\mathbf{F}}_S}{\mathbf{F}}^T_S\,. \end{aligned}$$

Finally, summing up the stresses of solid, blood and interstitial fluid given above, one obtains the total stress of the overall model as

42 $$\begin{aligned} {\mathbf{T}}^S=-p^{FR}\,{\mathbf{I}}\,+{\mathbf{T}}^S_{{\mathrm{eff}}}\,. \end{aligned}$$

In addition to the stresses, the direct momentum productions yield in thermodynamic equilibrium

43 $$\begin{aligned} \hat{\mathbf{p}}^B= & {} \Big (\varLambda -\varPsi ^I_I+\sum _\gamma c^{I\gamma }_m\frac{\partial \varPsi ^{I\gamma }_I}{\partial c^{I\gamma }_m}\Big )\,\text{ grad }\,n^B\nonumber \\&\quad -(s^B)^2\rho ^{BR}\frac{\partial \psi ^B}{\partial s^B}\,\text{ grad }\,n^S\nonumber \\= & {} \, p^{IR}\text{ grad }\,n^B-s^B\,p^{{\mathrm{dif}}}\,\,\text{ grad }\,n^S \,,\nonumber \\ \hat{\mathbf{p}}^{I\gamma }= & {} \Big (s^{I\gamma }\varLambda -\varPsi ^{I\gamma }_I+c^{I\gamma }_m\frac{\partial \varPsi ^{I\gamma }_I}{\partial c^{I\gamma }_m}\Big )\,\text{ grad }\,n^I+n^I\varLambda \text{ grad }\,n^{I\gamma }\nonumber \\= & {} \,\pi ^{I\gamma }\text{ grad }\,n^I+p^{IR}_m\,\text{ grad }\,n^{I\gamma }\,, \end{aligned}$$

where (34)–(38) have been used.

*Dissipation mechanism*: While considering (33) in thermodynamic equilibrium, the terms in brackets have to vanish for arbitrary velocity gradients and seepage velocities. However, beyond thermodynamic equilibrium, the dissipation mechanism allows the terms in brackets to depend on these terms. Basically, the dissipation mechanism *D* can be split in three portions yielding

44 $$\begin{aligned} D=D_T+D_{{\hat{p}}}+D_{{\hat{\rho }}}\ge 0\,, \end{aligned}$$

where $D_T$ addresses the frictional stresses of $\varphi ^B$ and $\varphi ^{I\gamma }$, while $D_{{\hat{p}}}$ and $D_{{\hat{\rho }}}$ describe the dissipative parts of the momentum and mass productions. As the frictional fluid stresses are negligible under creeping flow conditions, cf. (Ehlers 2020), $D_T$ vanishes, such that only $D_{{\hat{p}}}$ and $D_{{\hat{\rho }}}$ remain reading

45 $$\begin{aligned} D_{{\hat{p}}}= & {} \,\hat{\mathbf{p}}^B_{{\mathrm{dis}}}\,\cdot \,\mathbf{w}_B+\sum _\gamma \hat{\mathbf{p}}^{I\gamma }_{{\mathrm{dis}}}\,\cdot \,\mathbf{w}_{I\gamma }\ge 0\,,\nonumber \\ D_{{\hat{\rho }}}= & {} -{{\hat{\rho }}}^S\Big (\psi ^S+\frac{p^{SR}}{\rho ^{SR}}\,\Big )\nonumber \\&+\,\sum _\gamma {\hat{\rho }}^{I\gamma }{\Bigg [\,}\Big (\psi ^{I\gamma } +\frac{\pi ^{I\gamma }+s^{I\gamma }p^{IR}_m}{\rho ^{I\gamma }_I}\Big ) -\textstyle {\frac{1}{2}}{\mathbf{w}}_{I\gamma }\,\cdot \,\mathbf{w}_{I\gamma }\,\Bigg ]\,\ge 0\,, \end{aligned}$$

thus satisfying (44). Note that the relation $\frac{1}{2}\overset{\prime }{\mathbf{x}}_S\,\cdot \,\overset{\prime }{\mathbf{x}}_S-\overset{\prime }{\mathbf{x}}_S\,\cdot \,\overset{\prime }{\mathbf{x}}_{I\gamma } +\frac{1}{2}\overset{\prime }{\mathbf{x}}_{I\gamma }\,\cdot \,\overset{\prime }{\mathbf{x}}_{I\gamma }=\frac{1}{2}{\mathbf{w}}_{I\gamma }\,\cdot \,{\mathbf{w}}_{I\gamma }$ has been used to obtain (45)$_2$.

Taking a closer look at the dissipative parts of the momentum productions included in $D_{{{\hat{p}}}}$, one easily concludes to

46 $$\begin{aligned} \hat{\mathbf{p}}^B_{{\mathrm{dis}}}= & {}\, \hat{{{\mathbf{p}}}}^B-\,p^{IR}\text{ grad }\,n^B+s^B\,p^{\mathrm{dif}}\,\,\text{ grad }\,n^S=-\,{\mathbf{S}}^{BS}{\mathbf{w}}_B\,,\nonumber \\ \hat{\mathbf{p}}^{IL}_{{\mathrm{dis}}}= & {}\, \hat{{\mathbf{p}}}^{IL}-\,\pi ^{IL}\text{ grad }\,n^I-p^{IR}_m\,\text{ grad }\,n^{IL}\nonumber \\= & {} -{\mathbf{S}}^{ILS}{\mathbf{w}}_{IL}-\sum _\delta {\mathbf{S}}^{IL\delta }(\mathbf{w}_{IL}-{\mathbf{w}}_{I\delta })\,,\nonumber \\ \hat{\mathbf{p}}^{I\delta }_{{\mathrm{dis}}}= & {}\, \hat{\mathbf{p}}^{I\delta }-\,\pi ^{I\delta }\text{ grad }\,n^I-p^{IR}_m\,\text{ grad }\,n^{I\delta }\nonumber \\= & {} -{\mathbf{S}}^{I\delta S}{\mathbf{w}}_{I\delta }-\sum _\delta {\mathbf{S}}^{IL\delta }({\mathbf{w}}_{I\delta }-{\mathbf{w}}_{IL})\,, \end{aligned}$$

where ${\mathbf{S}}^{BS}$, ${\mathbf{S}}^{ILS}$, ${\mathbf{S}}^{IL\delta }$ and ${\mathbf{S}}^{I\delta S}$ are positive definite tensors describing a sort of friction between the individual components. Given (46), the dissipation mechanism $D_{{{\hat{p}}}}$ yields

47 $$\begin{aligned} D_{{\hat{p}}}= & {}\, {\mathbf{S}}^{BS}{\mathbf{w}}_B\,\cdot \,{\mathbf{w}}_B+\,\mathbf{S}^{ILS}{\mathbf{w}}_{IL}\,\cdot \,{\mathbf{w}}_{IL}\nonumber \\&+\,\sum _\delta [\,\underbrace{{\mathbf{S}}^{IL\delta }({\mathbf{w}}_{IL}-\mathbf{w}_{I\delta })\,\cdot \,{\mathbf{w}}_{IL}+{\mathbf{S}}^{IL\delta }(\mathbf{w}_{I\delta }-{\mathbf{w}}_{IL})\,\cdot \,{\mathbf{w}}_{I\delta }}_{\mathbf{S}^{IL\delta }({\mathbf{w}}_{IL}-{\mathbf{w}}_{I\delta })\,\cdot \,(\mathbf{w}_{IL}-{\mathbf{w}}_{I\delta })}]\ge 0\,. \end{aligned}$$

While the friction ${\mathbf{S}}^{I\delta S}$ between the interstitial fluid species and the solid is negligible and has thus been neglected, the remainder of friction tensors reads

48 $$\begin{aligned}\left. \begin{array}{l} {\mathbf{S}}^{BS}=(n^B)^2\mu ^{BR}(\mathbf{K}^{SB})^{-1}\\ {\mathbf{S}}^{ILS}=(n^{IL})^2\mu ^{ILR}({\mathbf{K}}^{SIL})^{-1} \end{array}\right\} \quad \text{ permeability } \text{ bound },\nonumber \\ \,{\mathbf{S}}^{IL\delta }\,\,\,\,\,=(n^I)^2R\theta c^{I\delta }_m(\mathbf{D}^{I\delta })^{-1}\,\hspace{2ex}\}\quad \text{ diffusivity } \text{ bound }. \end{aligned}$$

Therein, $\mu ^{BR}$ and $\mu ^{ILR}$ are the effective dynamic viscosities of the blood and the interstitial fluid solvent, $\mathbf{K}^{SB}$ and ${\mathbf{K}}^{SIL}$ are the intrinsic solid permeability tensors measured in $\hbox {m}^2$ with respect to the blood and interstitial fluid compartments. Finally, ${\mathbf{D}}^{I\delta }$ is the diffusion tensor of the solutes $\varphi ^{I\delta }$ in the solution $\varphi ^I$, while *R* is the universal gas constant. Note that in case of homogeneous permeabilities and diffusivities, $\mathbf{K}^{SB}$, ${\mathbf{K}}^{SIL}$ and ${\mathbf{D}}^{I\delta }$ reduce to

49 $$\begin{aligned} {\mathbf{K}}^{SB}=K^{SB}\,{\mathbf{I}}\,,\quad \mathbf{K}^{SIL}=K^{SI}\,{\mathbf{I}}\,,\quad {\mathbf{D}}^{I\delta }=D^{I\delta }\,\mathbf{I}\,, \end{aligned}$$

where the intrinsic permeabilities can be substituted by hydraulic conductivities through the relations

50 $$\begin{aligned} \frac{\mathbf{K}^{S\beta }}{\mu ^{\beta R}}=\frac{{\mathbf{K}}^{\beta }}{\rho ^{\beta R}|\mathbf{g}|}\, \quad \text{ and }\quad \frac{K^{S\beta }}{\mu ^{\beta R}}=\frac{k^{\beta }}{\rho ^{\beta R}|\mathbf{g}|}\,. \end{aligned}$$

In the above relation, $K^{S\beta }$ is given in $\hbox {m}^2$ and $k^\beta$ in m/s.

Concerning the second dissipation mechanism $D_{{\hat{\rho }}}$, one has to guarantee that $D_{{\hat{\rho }}}$ is always positive or zero yielding

51 $$\begin{aligned} D_{{\hat{\rho }}}= & {} -{{\hat{\rho }}}^S\Big (\psi ^S+\frac{p^{SR}}{\rho ^{SR}}\,\Big )\nonumber \\&+\,\sum _\delta {\hat{\rho }}^{I\delta }\Big (\psi ^{I\delta } +\frac{\pi ^{I\delta }+s^{I\delta }p^{IR}_m}{\rho ^{I\delta }_I}\Big )\ge 0\,, \end{aligned}$$

where the mass-specific kinetic seepage energies of the liquid solvents have been neglected. Furthermore, ${\hat{\rho }}^{IL}$ dropped out as the liquid solvent does not contribute to the mass production process. By the use of the mass-specific chemical potentials

52 $$\begin{aligned}&{{\bar{\mu }}}^S=\psi ^S+\frac{p^{SR}}{\rho ^{SR}} \quad \text{ and }\quad {{\bar{\mu }}}^{I\delta }=\psi ^{I\delta } +\frac{p^{I\delta }}{\rho ^{I\delta }_I}\,,\nonumber \\&\text{ where }~~~p^{I\delta }:=s^{I\delta }p^{IR}_m+\pi ^{I\delta } \end{aligned}$$

defines the partial pressure of $\varphi ^{I\delta }$ as a component of $\varphi ^I$, the dissipation mechanism $D_{{\hat{\rho }}}$ can finally be obtained as

53 $$\begin{aligned} D_{{\hat{\rho }}}&=-{{\hat{\rho }}}^S\,{{\bar{\mu }}}^S-\sum _\delta {\hat{\rho } }^{I\delta }{{\bar{\mu }}}^{I\delta }\ge 0\,. \end{aligned}$$

Note that the chemical potentials ${{\bar{\mu }}}^{I\delta }$ are related to their molar counterparts in (34) through $\mu ^{(\,\cdot \,)}_m=M^{(\,\cdot \,)}_m{{\bar{\mu }}}^{(\,\cdot \,)}$.

### Flow and transport of pore-liquid components

*Interstitial fluid mixture*: As the interstitial fluid $\varphi ^I$ consists of the liquid solvent $\varphi ^{IL}$ and solutes $\varphi ^{I\delta }$, the chemical potentials, osmotic pressures and mechanical pressures of these components have to be specified. Given (34), chemical potentials and osmotic pressures of the species $\varphi ^{I\gamma }$ depend on the volume-specific free energies $\varPsi ^{I\gamma }_I$ that can be given as

54 $$\begin{aligned} {\varPsi ^{I\gamma }_I}=c^{I\gamma }_m\mu ^{I\gamma }_{0\,m}+c^{I\gamma }_m R\theta \, (\ln c^{I\gamma }_m-1), \end{aligned}$$

with $\mu ^{I\gamma }_{0\,m}$ as the so-called reference or standard-state chemical potential, such that

55 $$\begin{aligned} \mu ^{I\gamma }_m= & {} \mu ^{I\gamma }_{0\,m}+R\theta \ln c^{I\gamma }_m\,,\nonumber \\ \pi ^{I\gamma }= & {} R\theta c^{I\gamma }_m \quad \text{ and }\quad \pi ^I=R\theta \sum _\gamma c^{I\gamma }_m\,. \end{aligned}$$

Based on (37), the effective interstitial fluid pressure is

56 $$\begin{aligned} p^{IR}=p^{IR}_m+\pi ^I \quad \text{ with }\quad p^{IR}_m=p^{IR}-R\theta \sum _\gamma c^{I\gamma }_m\,. \end{aligned}$$

In (37) as well as in (56), there has no difference been made between the liquid solvent and the solutes concerning the definition of the potential $\varPsi ^{I\gamma }_I$. As a result, $p^{IR}$ has to be found from the boundary-value problem, while $\varLambda =p^{IR}_m$ results from (56).

Furthermore, combining (46) and (48), the direct momentum productions read

57 $$\begin{aligned} \hat{\mathbf{p}}^B =& {} \,p^{IR}\text{ grad }\,n^B-s^B\,p^{{\mathrm{dif}}}\,\,\text{ grad }\,n^S-(n^B)^2\mu ^{BR}({\mathbf{K}}^{SB})^{-1}\mathbf{w}_B\,,\nonumber \\ \hat{\mathbf{p}}^{IL}= &{} \,\pi ^{IL}\text{ grad }\,n^I+p^{IR}_m\,\text{ grad }\,n^{IL}\nonumber \\ &-\,(n^{IL})^2\mu ^{ILR}({\mathbf{K}}^{SIL})^{-1}{\mathbf{w}}_{IL}\nonumber \\ &-\,\sum _\delta (n^I)^2R\theta c^{I\delta }_m(\mathbf{D}^{I\delta })^{-1}({\mathbf{w}}_{IL}-{\mathbf{w}}_{I\delta })\,,\nonumber \\ \hat{{\mathbf{p}}}^{I\delta }= & {} \,\pi ^{I\delta }\text{ grad }\,n^I+p^{IR}_m\,\text{ grad }\,n^{I\delta }\nonumber \\ &-\,(n^I)^2R\theta c^{I\delta }_m({\mathbf{D}}^{I\delta })^{-1}(\mathbf{w}_{I\delta }-{\mathbf{w}}_{IL})\,. \end{aligned}$$

Given the above equations, (57)$_{2,3}$ result in

58 $$\begin{aligned} \hat{\mathbf{p}}^I= & {} \,\hat{\mathbf{p}}^{IL}+\sum _\delta \hat{\mathbf{p}}^{I\delta }\nonumber \\= & {} \,p^{IR}\text{ grad }\,n^I-(n^{IL})^2\mu ^{ILR}(\mathbf{K}^{SIL})^{-1}{\mathbf{w}}_I\,, \end{aligned}$$

where ${\mathbf{w}}_{IL}\approx {\mathbf{w}}_I$ has been used.

Inserting $\hat{\mathbf{p}}^I$ together with ${\mathbf{T}}^I$ from (37) in the fluid momentum balance (4)$_2$ yields under quasi-static conditions

59 $$\begin{aligned} {\mathbf{0}}= & {} \,\hbox {div}\,{\mathbf{T}}^I\,+\,\rho ^I\,{\mathbf{g}}\,+\, {\hat{\mathbf{p}}}^I \nonumber \\= & {} -n^I\text{ grad }\,p^{IR}+n^I\rho ^{IR}\mathbf{g}-(n^{IL})^2\mu ^{ILR}({\mathbf{K}}^{SIL})^{-1}{\mathbf{w}}_I\,,\nonumber \\&\quad \text{ and } \text{ thus }\quad n^I{\mathbf{w}}_I=-\frac{\mathbf{K}^{SIL}}{\mu ^{ILR}}(\text{ grad }\,p^{IR}-\rho ^{IR}{\mathbf{g}}), \end{aligned}$$

with $n^I{\mathbf{w}}_I$ as the filter velocity of the interstitial fluid.

For the solutes $\varphi ^{I\delta }$, the same procedure yields with the aid of $\hat{\mathbf{p}}^ {I\delta }$ and ${\mathbf{T}}^ {I\delta }$ from (35)

60 $$\begin{aligned} {\mathbf{0}}= & {} \,\hbox {div}\,{\mathbf{T}}^{I\delta }\,+\,\rho ^{I\delta }\,\mathbf{g}\,+\, \hat{\mathbf{p}}^{I\delta }\nonumber \\= & {} -n^I\text{ grad }\,\pi ^{I\delta }-n^{I\delta }\text{ grad } \,p^{IR}_m+n^{I\delta }\rho ^{I\delta R}{\mathbf{g}}\nonumber \\&-\,(n^I)^2R\theta c^{I\delta }_m({\mathbf{D}}^{I\delta })^{-1}\mathbf{d}_{I\delta }\,, \end{aligned}$$

where the diffusion velocity ${\mathbf{d}}_{I\delta }=\mathbf{w}_{I\delta }-{\mathbf{w}}_I$ together with ${\mathbf{w}}_I\approx {\mathbf{w}}_{IL}$ has been used. Solving (60) with respect to ${\mathbf{d}}_{I\delta }$ leads to

61 $$\begin{aligned}&n^Ic^{I\delta }_m{\mathbf{d}}_{I\delta }=-\frac{\mathbf{D}^{I\delta }}{R\theta } \bigl [\,\text{ grad }\,\pi ^{I\delta }+\underbrace{s^{I\delta }\big (\text{ grad }\,p^{IR}_m -\rho ^{I\delta R}\mathbf{g}\big )}_{extension}\,\bigr ]\,. \end{aligned}$$

In this equation, the extension can be neglected with respect to the fact that ${\mathbf{S}}^{I\delta S}$ has been neglected in (46)$_3$, cf. the remark between (47) and (48). As ${\mathbf{S}}^{I\delta S}$ would basically have the same shape as the friction tensor ${\mathbf{S}}^{ILS}$ from (48)$_2$, neglecting the extension means to neglect the seepage velocity $\mathbf{w}_{I\delta }$ of the solutes $\varphi ^{I\delta }$ compared to the other terms in (61). Formally, $\mathbf{w}_{I\delta }$ would be governed by

62 $$\begin{aligned} {\mathbf{S}}^{I\delta S}{} \mathbf{w}_{I\delta }=-s^{I\delta }\big ( \text{ grad }\,p^{IR}_m -\rho ^{I\delta R}{\mathbf{g}}\big )\,. \end{aligned}$$

Following this, the momentum balance of the solutes reduces to

63 $$\begin{aligned}&n^Ic^{I\delta }_m{\mathbf{d}}_{I\delta }=-\frac{\mathbf{D}^{I\delta }}{R\theta }\text{ grad }\,\pi ^{I\delta }=-\mathbf{D}^{I\delta }\text{ grad }\,c^{I\delta }_m\,, \end{aligned}$$

where (55)$_2$ has been used.

*Blood plasma*: The momentum balance of the blood has to be combined with the momentum production $\hat{\mathbf{p}}^B$ from (46)$_1$ and the stress tensor $\mathbf{T}^B$ from (38). Thus,

64 $$\begin{aligned} {\mathbf{0}}= & {} \,\hbox {div}\,{\mathbf{T}}^B+\,\rho ^B\,{\mathbf{g}}\,+\, {\hat{\mathbf{p}}}^B \nonumber \\= & {} -n^B\text{ grad }\,p^{BR}-p^{BR}\text{ grad }\,n^B+p^{IR}\text{ grad }\,n^B-s^Bp^{{\mathrm{dif}}}\text{ grad }\,n^S\nonumber \\&+\,n^B\rho ^{BR}{\mathbf{g}}-(n^B)^2\mu ^{BR}(\mathbf{K}^{SB})^{-1}{\mathbf{w}}_B\,,\quad \text{ such } \text{ that }\nonumber \\ n^B{\mathbf{w}}_B= & {} -\frac{\mathbf{K}^{SB}}{\mu ^{BR}}\big [\text{ grad }\,p^{BR}+\frac{p^{{\mathrm{dif}}}}{s^B}\text{ grad }\,s^B-\rho ^{BR}{\mathbf{g}}\big ]\,. \end{aligned}$$

While the pressures $p^{BR}$ and $p^{IR}$ have to be found from the boundary-value problem, the blood saturation $s^B$ has to be constructed constitutively under consideration of (39). Alternatively, one pressure and $s^B$ can be obtained from the boundary-value problem, such that (39) can be used directly for the determination of the missing pressure.

*Blood saturation and angiogenesis:* As has been said before, the volume fraction $n^B=s^Bn^P$ of the blood plasma cannot be found from the solid deformation, only the porosity $n^P=s^I+s^B$ is obtained as a function of $\text{ det }\,{\mathbf{F}}_S$ through $n^P=1-n^S$ with $n^S$ after (17). With this in mind, $s^B$ can be coupled to the angiogenesis in case that relation (39) between the difference pressure $p^{{\mathrm{dif}}}$ and $s^B$ is satisfied and the potential $\rho ^{BR}\psi ^B$ is positive in the range $0\le s^B\le 1$.

**Fig. 3:**
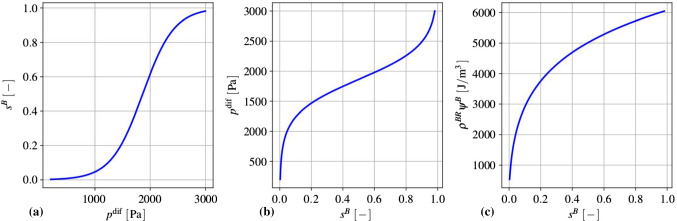
**a** Blood-plasma saturation as sigmoid function $s^B(p^{{\mathrm{dif}}})$, **b** pressure difference as inverse sigmoid function $p^{{\mathrm{dif}}}(s^B)$ and **c** blood-plasma potential (stored Helmholtz free energy) $\rho ^{BR}\psi ^B(s^B)$ illustrated for $0.06\le s^B\le 1$ with $\alpha ^{B}=1863.08~{\mathrm{Pa}}$, $\mu ^{A}=0.0035~{\mathrm{kg/J}}$ and $\mu ^{B}=0.0035~{\mathrm{1/Pa}}$ at an angiogenesis energy of $\rho ^{BR}\psi ^{B,\,{\mathrm{angio}}}=0$

Following this, the saturation function should account for blood vessel growth by angiogenesis induced by vascular endothelial growth factors (VEGF) during the cancer cell proliferation as well as for elastic deformations of arterial walls during a change in the local pressure conditions. In this regard, the saturation curve $s^B$ depends on the pressure difference $p^{{\mathrm{dif}}}$, cf. Ehlers and Wagner (2015), and a mass-specific Helmholtz free energy $\psi ^{B,\,\text {angio}}$ related to the angiogenesis process. Basically, growth processes can be described by smooth Heaviside or sigmoid functions, cf. Hubbard and Byrne (2013), here defined as

65 $$\begin{aligned} \text{ sig }\,(x)=\frac{e^x}{1+e^x}= \frac{\text{ exp }\,(x)}{1+\text{ exp }\,(x)}\,. \end{aligned}$$

For arbitrary but growing *x*, $\text{ sig }\,(x)$ has values between zero and one with $\text{ sig }\,(x=0)=0.5$. By the introduction of the constants $\alpha$, $\beta$ and $\gamma$, the use of the argument $\alpha x$ instead of *x* changes the steepness of the curve with increasing gradients for $\alpha >1$ and decreasing gradients for $0<\alpha <1$. Substituting 1 by $\beta >1$ lowers the value of $\text{ sig }\,(x=0)$ with increasing values for $0<\beta <1$. Finally, substituting $\text{ exp }\,(x)$ by $\text{ exp }\,(\alpha x+\gamma )$ shifts the curve along the x-axis. Extending (65) in this sense yields

66 $$\begin{aligned} f\,(x)=\frac{\text{ exp }\,(\alpha x+\gamma )}{\beta +\text{ exp }\,(\alpha x+\gamma )}\,. \end{aligned}$$

Applying the structure of (66) to $s^B(p^{{\mathrm{dif}}})$ results in

67 $$\begin{aligned} s^B(p^{{\mathrm{dif}}})=\frac{\text{ exp }\,(\mu ^B p^{{\mathrm{dif}}}+\mu ^A\psi ^{B,\,{\mathrm{angio}}})}{\text{ exp }\,(\mu ^B\alpha ^B)+\text{ exp }\,(\mu ^B p^{{\mathrm{dif}}}+\mu ^A\psi ^{B,\,{\mathrm{angio}}})}\,, \end{aligned}$$

where $\alpha$ has been substituted by $\mu ^B$, $\beta$ by $\text{ exp }\,(\mu ^B\alpha ^B)$ and $\gamma$ by $\mu ^A\psi ^{B,\,\mathrm angio}$. As $p^{{\mathrm{dif}}}$ is given in Pa, $\mu ^B$ has the unit $1/{\mathrm{Pa}}$. $\psi ^{B,\,{\mathrm{angio}}}$ is the stored angiogenesis energy per unit mass, such that its unit is J/kg. Thus, $\mu ^A$ is given in ${\mathrm{kg}}/{\mathrm{J}}$. Finally, as $\mu ^B$ is given in 1/Pa, $\alpha ^B$ has the unit Pa of a pressure.

Initially, the volume fractions of the blood plasma and the interstitial fluid are chosen as $n^B_0=0.05$ and $n^I_0=0.20$, cf. Nicholson (2001) and citations therein, such that the initial blood saturation results in $s^B_0=n^B_0/n^P_0=0.20$. This value is also obtained by the use of (67) with $\alpha ^B=1863.08~{\mathrm{Pa}}$, $\mu ^A=0.0035\,\hbox {kg/J}$ and $\mu ^B=0.0035~1/{\mathrm{Pa}}$, thus also establishing an initial pressure difference of $p^{{\mathrm{dif}}}_0=p^{BR}_0-p^{IR}_0=1467~{\mathrm{Pa}}$ at $\psi ^{B,\,\mathrm angio}_0=0$. The corresponding sigmoid function is displayed in Fig. 3a.

The above pressure difference $p^{{\mathrm{dif}}}_0$ originates from the blood and interstitial fluid pressures in the initial state, where the latter corresponds to $p^{IR}_0 = 258$ Pa $\approx \,1.9$ mm Hg and originates from the mean interstitial fluid pressure in the brain, cf. Boucher et al. (1997). Concerning the blood pressure, a homogenisation approach can be taken into account favouring the blood pressure of small blood vessels that are equally distributed within the brain tissue, where larger arteries and veins are neglected. As a result, the implemented blood pressure of $p^{BR}_0 = 1725$ Pa $\approx \,13$ mm Hg is below the observed human mean arterial blood pressure but accepted as the mean blood pressure in the brain.

Given (67), this equation can be solved with respect to $p^{{\mathrm{dif}}}(s^B)$ yielding the inverse sigmoid function

68 $$\begin{aligned} p^{{\mathrm{dif}}}(s^B)=\frac{1}{\mu ^B}\Bigg [\ln \Bigg (\frac{s^B \text{ exp }\,(\mu ^B\alpha ^B)}{1-s^B}\Bigg )-\mu ^A\psi ^{B,\,{{\mathrm{angio}}}}\Bigg ] \end{aligned}$$

displayed in Fig. 3b. The potential corresponding to $p^{{\mathrm{dif}}}$ is obtained from (39) through integration as

69 $$\begin{aligned} \rho ^{BR}\psi ^B(s^B)= & {} \rho ^{BR}\int ^{{s^B}}_{{s_0}} \frac{\partial \psi ^B({\tilde{s}}^B)}{\partial {\tilde{s}}^B}\,\mathrm{d}{\tilde{s}}^B\nonumber \\= & {} \int ^{{s^B}}_{{s_0}}\frac{p^{{\mathrm{dif}}}({\tilde{s}}^B)}{{\tilde{s}}^B}{\mathrm{d}}\,{\tilde{s}}^B\nonumber \\= & {} \frac{1}{\mu ^B}\,\Bigg [\,\frac{1}{2}\,\big [\,\ln \,(s^B \text{ exp }\,(\mu ^B\alpha ^B))\,\big ]^2, (\mu ^A\psi ^{B,\,\mathrm{angio}})\ln \,s^B\,\Bigg ]\nonumber \\&+\, \frac{1}{\mu ^B}\,\Bigg [\,-\int ^{{s^B}}_{{s_0}} \frac{\ln (1-{\bar{s}}^B)}{{\bar{s}}^B}\,\mathrm{d}{\bar{s}}^B\,\Bigg ]+\rho ^{BR}\psi ^B_0\,. \end{aligned}$$

In the above potential function $\rho ^{BR}\psi ^B$, the term in the last brackets cannot formally be integrated, as it is a so-called integral logarithm of the dilogarithm type ${\mathrm{Li}}_2(s^B)$. Instead, ${\mathrm{Li}}_2(s^B)$ can be computed as a sum via $\mathrm{Li}_2(s^B)=\sum ^\infty _{k=1} ({s^B})^k/k^2$. The potential $\rho ^{BR}\psi ^B$ is plotted in Fig. 3c.

When the growing cancer cell cluster has consumed most of the nutrients that could reach the cell cluster by diffusion, the cancer cells start to send out VEGF proteins to initialise blood vessel growth necessary for their further proliferation, cf. Finley and Popel (2013). In particular, blood vessel growth triggers the creation of new vessels from which nutrients can diffuse into the interstitial fluid or directly into the cancerous tissue. In this regard, the angiogenesis Helmholtz free energy term $\psi ^{B,\,{\mathrm{angio}}}$ is chosen to be proportional to the VEGF concentration $c^{IV}_m$ and the threshold ${\bar{c}}^{IV}_m$, thus initiating the blood growth via

70 $$\begin{aligned} \psi ^{B,\,\text {angio}} = \alpha ^{\text {angio}}(c^{IV}_m - {\bar{c}}^{IV}_m) \,. \end{aligned}$$

Therein, the parameter $\alpha ^{\text {angio}}=8.625\times 10^{6}\,({\mathrm{mol~J)/(kg)^2}}$ relates the molar concentration of VEGF to $\psi ^{B,\,\text {angio}}$, while ${\bar{c}}^{IV}_m=2.5\,\times 10^{-11}{\mathrm{mol/m^3}}$, both triggering the increase or decrease in the blood saturation $s^B$.

### Brain skeleton and growth-dependent solid elasticity

Although brain material generally exhibits a viscoelastic material response, we refrain from including the viscous effects of the brain skeleton with respect to the extremely slow deformations resulting from tumour growth. On the other hand, the viscosity of the interstitial fluid and the blood is fully considered.

Thus, the material law for the brain skeleton is described by an elasticity law of neo-Hookean type under consideration of growth phenomena and has to be formulated for the solid stress tensor obtained from (41):

71 $$\begin{aligned} {\mathbf{T}}^S=-n^S p^{FR}\,{\mathbf{I}}\,+\mathbf{T}^S_{{\mathrm{eff}}} \quad \text{ with }\quad \mathbf{T}^S_{{\mathrm{eff}}}=\rho ^S\frac{\partial \psi ^S}{\partial \mathbf{F}_S}{\mathbf{F}}^T_S\,. \end{aligned}$$

**Fig. 4:**
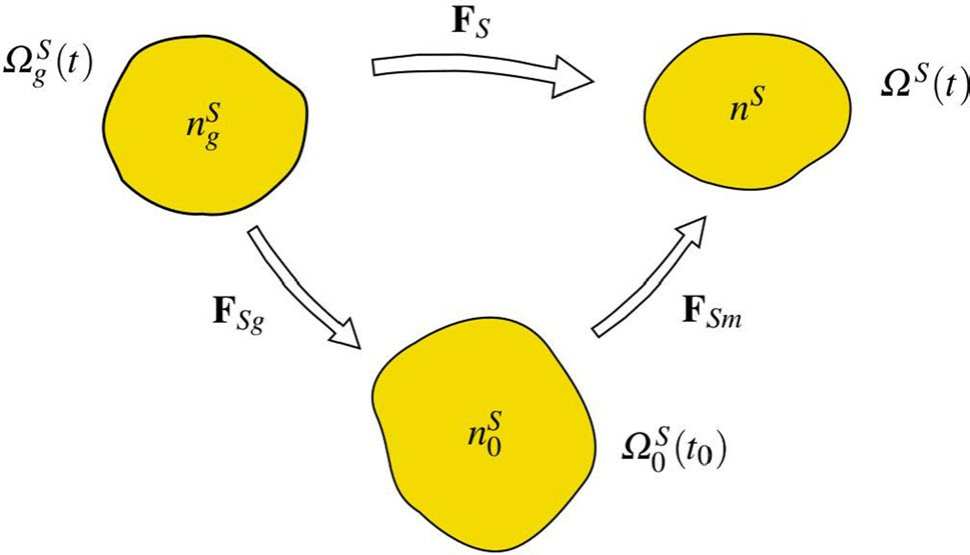
Multiplicative split of the deformation gradient and corresponding configurations

Therein, the deformation gradient ${\mathbf{F}}_S$ can be split, as in thermoelasticity or in elasto-plasticity, into two parts, a purely mechanical part ${\mathbf{F}}_{Sm}$ and a growth-dependent part $\mathbf{F}_{Sg}$, also compare "Appendix (b)". This split can be motivated on the basis of the time-integrated solid volume balance (17) reading

72 $$\begin{aligned} n^S= \underbrace{\displaystyle \,n^S_{0}\,\exp \,\Big (\, \int ^t_{t_0} \frac{{\hat{n}}^S}{n^S} \,{\mathrm{d}}{\tilde{t}} \,\Big )}_{n^S_g}\,(\det {\mathbf{F}}_S)^{-1}=n^S_g\,(\det \mathbf{F}_S)^{-1} \end{aligned}$$

where $n^S_g=:n^S_0\,\det {\mathbf{F}}_{Sg}$ is taken as an a- priori constitutive equation for $\det {\mathbf{F}}_{Sg}$ . As the integration of the solid volume balance (15)$_1$ makes use of the relation $\text{ div }\,({\mathbf{u}}_S)^\prime _S=(\text{ det }\,\mathbf{F}_S)^{-1}(\text{ det }\,{\mathbf{F}}_S)^\prime _S$, it is evident that $\det {\mathbf{F}}_S$ rules the relation between the solid volume fraction $n^S$ of the current configuration and the growth-depending volume fraction $n^S_g$. This implies that $n^S_g$ as a function of growth apparently substitutes the reference volume fraction $n^S_0$. Only in case that no growth appears, such that $n^S_g=n^S_0$ with $\det {\mathbf{F}}_{Sg}=1$, the standard relation $\det \mathbf{F}_S=n^S_0/n^S$ is recovered for materially incompressible solid skeletons. As a result of the above, the multiplicative split of growth-depending materials results in

73 $$\begin{aligned} {\mathbf{F}}_S={\mathbf{F}}_{Sm}{\mathbf{F}}_{Sg} ~~\text{ with }~~ \text{ det }\,{\mathbf{F}}_S=\text{ det }\,\mathbf{F}_{Sm}\,\text{ det }\,{\mathbf{F}}_{Sg}=:J_{Sm}\,J_{Sg}\,, \end{aligned}$$

where the Jacobian determinants can be expressed by solid volume fractions via

74 $$\begin{aligned} \text{ det }\,{\mathbf{F}}_S=n^S_g/n^S\,,\quad \text{ det }\,{{\mathbf{F}}}_{Sm}=n^S_0/n^S\,,\quad \text{ det }\,{\mathbf{F}}_{Sg}=n^S_g/n^S_0\,, \end{aligned}$$

also cf. Fig. 4. As can be seen from this figure, the purely mechanical deformation included in ${\mathbf{F}}_{Sm}$ takes place between the reference configuration $\varOmega ^S_0(t_0)$ and the current configuration $\varOmega ^S(t)$, while the total deformation represented by $\mathbf{F}_S$ is between the growth configuration $\varOmega ^S_g(t)$ and $\varOmega ^S(t)$. This is different from the deformation split in thermoelasticity and in elasto-plasticity, where an intermediate configuration $\varOmega ^S_{{\mathrm{int}}}(t)$ is situated between $\varOmega ^S_0$ and $\varOmega ^S$ and where the purely mechanical, respectively, the purely elastic deformation takes place between $\varOmega ^S_{{\mathrm{int}}}$ and $\varOmega ^S$. This is also different from the assumption made by Rodrigues et al. (1994), Ambrosi and Preziosi (2002) or Garikipati et al. (2004) who all assumed the standard decomposition of ${\mathbf{F}}_S$ in mechanical and growth-dependent parts.

On the other hand, Fig. 4 can also be interpreted differently. In this case, $\varOmega ^S_g$ can be taken as a time-depending reference configuration, while $\varOmega ^S_0$ would act as an intermediate configuration at $t_0$. Anyway, the order of ${\mathbf{F}}_S$ shifting from $\varOmega ^S_g$ to $\varOmega ^S_0$ through $\mathbf{F}_{Sg}$ and from $\varOmega ^S_0$ to $\varOmega ^S$ through ${\mathbf{F}}_{Sm}$ would then be fully classical.

Describing tumour growth, we make the assumption that ${\mathbf{F}}_{Sg}$ is a purely volumetric deformation yielding

75 $$\begin{aligned} {\mathbf{F}}_{Sg}=(\text{ det }\,{\mathbf{F}}_{Sg})^{1/3}\,\mathbf{I}\,, \end{aligned}$$

such that

76 $$\begin{aligned} {\mathbf{F}}_S=(\text{ det }\,{\mathbf{F}}_{Sg})^{1/3}\,\mathbf{F}_{Sm}\,. \end{aligned}$$

With (75), we proceed from the same type of constitutive equation as has been used in thermoelasticity by Lu and Pister (1975).

Based on (73), (75) and (76), the basic deformation tensors read

77 $$\begin{aligned} {\mathbf{C}}_S= & {} \, {\mathbf{F}}^T_S{\mathbf{F}}_S= {\mathbf{F}}^T_{Sg}({\mathbf{F}}^T_{Sm}{} \mathbf{F}_{Sm}){\mathbf{F}}_{Sg}= {\mathbf{F}}^T_{Sg}{\mathbf{C}}_{Sm}{\mathbf{F}}_{Sg}\nonumber \\= & {} \, (\text{ det }\,{\mathbf{F}}_{Sg})^{2/3}\,{\mathbf{C}}_{Sm}\,,\nonumber \\ {\mathbf{B}}_S= & {} \, {\mathbf{F}}_S{\mathbf{F}}^T_S={\mathbf{F}}_{Sm}({\mathbf{F}}_{Sg}{} \mathbf{F}^T_{Sg}){\mathbf{F}}^T_{Sm}={\mathbf{F}}_{Sm}{\mathbf{B}}_{Sg}{\mathbf{F}}^T_{Sm}\nonumber \\= & {} \, (\text{ det }\,{\mathbf{F}}_{Sg})^{2/3}\,{\mathbf{B}}_{Sm}\,. \end{aligned}$$

Note in passing that ${\mathbf{C}}_S$ and ${\mathbf{B}}_S$ are the right and left Cauchy deformation tensors situated in $\varOmega _g$ and $\varOmega$, while ${\mathbf{C}}_{Sm}$ and ${\mathbf{B}}_{Sg}$ both correspond to in $\varOmega _0$.

As in thermoelasticity, the strain energy function does only depend on the elastic contribution of the deformation. In thermoelasticity, this is based on the fact that an unconfined body extends volumetrically when heated without exhibiting stresses. However, under fully confined boundary conditions meaning that no volumetric extension is possible, the overall volumetric strain is zero but splits into a thermal and a mechanical part of equal size. Here, the mechanical part induces stresses to compensate the heat extension.

Comparing volumetric growth to extension under heat makes apparent that growth only induces stresses when a free growing is hindered. Following this, the solid strain energy function is only based on the elastic part of the deformation governed by ${\mathbf{C}}_{Sm}$. A detailed derivation of the following elasticity law is found in "Appendix (c)".

Based on (71), the effective stress can be modified towards

78 $$\begin{aligned}&{\mathbf{T}}^S_{{\mathrm{eff}}}=\rho ^S\frac{\partial \psi ^S}{\partial {\mathbf{F}}_S}{} \mathbf{F}^T_S=2\,(\text{ det }\,{\mathbf{F}}_{Sm})^{-1}{\mathbf{F}}_S\frac{\partial W^S}{\partial {\mathbf{C}}_S}{\mathbf{F}}^T_S \end{aligned}$$

with $W^S:=\rho ^S_0\psi ^S$ as the hyperelastic solid strain energy including the partial solid density $\rho ^S_0=n^S_0\,\rho ^{SR}$ of the solid’s reference configuration at $t_0$. Following the argumentation by Ehlers and Eipper (1999), a nonlinear strain energy function of neo-Hookean type for porous solid materials formulated in the first and third invariant of $\mathbf{C}_{Sm}$, $I_m={\mathbf{C}}_{Sm}\cdot \,{\mathbf{I}}\,$ and $III_m=\text{ det }\,{\mathbf{C}}_{Sm}$, is given by

79 $$\begin{aligned}&W^S=\textstyle {\frac{1}{2}}\,\mu ^S_0\,(\,I_m-3)-\mu ^S_0\ln J_{Sm}+U^S(J_{Sm})\nonumber \\&\text{ with }\quad U^S(J_{Sm})=\lambda ^S_0(1-n^S_0)^2 \Bigg (\frac{J_{Sm}-1}{1-n^S_0}-\ln \frac{J_{Sm}-n^S_0}{1-n^S_0}\Bigg )\,, \end{aligned}$$

where $J_{Sm}=(\text{ det }\,{\mathbf{C}}_{Sm})^{1/2}$, while $\lambda ^S_0$ and $\mu ^S_0$ are the Lamé constants defined with respect to the solid reference configuration $\varOmega ^S_0$. Based on (78) and (79), the solid effective stress reads

80 $$\begin{aligned} {\mathbf{T}}^S_{{\mathrm{eff}}}=J_{Sm}^{-1}\,\Bigg [\,2\,\mu ^S_0\,\mathbf{K}_{Sm}+\lambda ^S_0(1-n^S_0)^2\Bigg (\frac{J_{Sm}}{1-n^S_0}-\frac{J_{Sm}}{J_{Sm}-n^S_0}\Bigg )\,\mathbf{I}\,\Bigg ] \end{aligned}$$

with ${\mathbf{K}}_{Sm}=\frac{1}{2}({\mathbf{B}}_{Sm}-\,{\mathbf{I}}\,)$ as the mechanical part of the Karni–Reiner strain $\mathbf{K}_S=\frac{1}{2}({\mathbf{B}}_S-\,{\mathbf{I}}\,)$. cf. Ehlers (2018b).

**Fig. 5:**
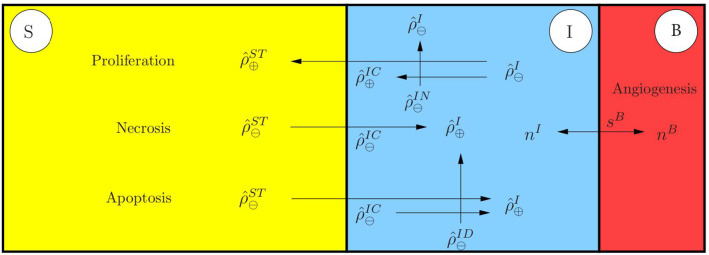
Overview of considered mass interactions in the metastasis model. Proliferation appears through nutrient consumption (upper left), whereas atrophy (upper left) is either related to insufficient nutrient supply, necrosis or the presence of a drug, apoptosis. These processes interchange mass between the solid and the interstitial fluid. The process of angiogenesis couples the mass exchange between the interstitial fluid and the blood which, in contrast to density productions, is described within the constitutive formulation of the saturation function

In the literature, the neo-Hookean term $2\,\mu ^S_0\,{\mathbf{K}}_{Sm}$ is often modified, for example, for the inclusion of anisotropy, cf. Ehlers and Wagner (2015), or for the inclusion of different material properties under tension and compression, cf. Comellas et al. (2020). In the present case, these features are of minor importance, as tumour growth and atrophy initiate pure volumetric deformations.

As the Karni–Reiner strain ${\mathbf{K}}_{Sm}$ and the Jacobian $J_{Sm}$ only represent the mechanical part of deformation and strain, these terms have to be substituted by the total deformation represented by ${\mathbf{K}}_{S}$ and $J_{S}=\text{ det }\,{\mathbf{F}}_S$ and the growth-dependent deformation through (73) and (75). As a result, the growth-dependent elasticity law yields

81 $$\begin{aligned} {\mathbf{T}}^S_{{\mathrm{eff}}}= & {} \frac{J_{Sg}}{J_{S}}\,\Bigg [\,\mu ^S_0\,(J^{-2/3}_{Sg}\mathbf{B}_{S}-\,{\mathbf{I}}\,) \nonumber \\&+\,\lambda ^S_0(1-n^S_0)^2\Bigg (\frac{{J_{S}}/{J_{Sg}}}{1-n^S_0} -\frac{{J_{S}}/{J_{Sg}}}{{J_{S}}/{J_{Sg}}-n^S_0}\Bigg )\,\mathbf{I}\,\Bigg ]\,. \end{aligned}$$

### Metastatic mechanisms

#### Basic setting

The mass production terms of the model govern the biological processes occurring during the evolution of tumours. These quantities have to be derived constitutively in a thermodynamically consistent way meaning that they have to fulfil the thermodynamical restrictions (53).

For the further understanding of the mass interaction process, recall that ${\hat{\rho }}^S+{\hat{\rho }}^I=0$ and that ${\hat{\rho }}^I$ splits into contributions ${\hat{\rho }}^{I\delta }$ of the interstitial fluid solutes $\varphi ^{I\delta }$ with $\delta =\{N,C,V,D\}$, nutrients, cancer cells, VEGF and drugs. As the individual ${\hat{\rho }}^{I\delta }$ can interchange with the solid skeleton, each ${\hat{\rho }}^{I\delta }$ has a solid counterpart ${\hat{\rho }}^{S\delta }$ in ${\hat{\rho }}^S$, such that

82 $$\begin{aligned} {\hat{\rho }}^I=-{\hat{\rho }}^S \quad \text{ and }\quad {\hat{\rho }}^{I\delta }=-{\hat{\rho }}^{S\delta }\,. \end{aligned}$$

Thus, (53) can be rewritten to yield

83 $$\begin{aligned}&D_{{\hat{\rho }}}=\sum _\delta \big [\,{{\hat{\rho }}}^{S\delta }\, ({{\bar{\mu }}}^{I\delta }-{{\bar{\mu }}}^S)\,\big ]\,\ge 0\,. \end{aligned}$$

Furthermore, the individual production terms generally split in their gains ${{\hat{\rho }}}^{I\delta }_{\oplus }$ and losses ${{\hat{\rho }}}^{I\delta }_{\ominus }$ following Krause et al. (2012):

84 $$\begin{aligned} {{\hat{\rho }}}^{I\delta }_*={{\hat{\rho }}}^{I\delta }_{\oplus ,*} -{{\hat{\rho }}}^{I\delta }_{\ominus ,*}\,. \end{aligned}$$

Therein, $*$ is a placeholder for the source of gains and losses. In the following, mass interactions for biologically induced metastatic processes, proliferation, necrosis and apoptosis, cf. Fig. 5, are stated for the solid skeleton and the interstitial fluid.

#### Mass production terms of the metastatic solid skeleton

Basically, nutrients supply the healthy brain as well as the metastatic tumours with energy for basal reactions as well as for their proliferation. Here, the metastatic proliferation ${\hat{\rho }}^{ST}_{\oplus ,\,IN}$ as a part of ${{\hat{\rho }}}^S$ corresponds to its nutrient consumption ${\hat{\rho }}^{IN}_{\ominus ,\,ST}$ as a part of ${{\hat{\rho }}}^I$.

In the present study, only those nutrients are taken into consideration that are consumed by the cancer cells during proliferation or basal reactions, cf. Guppy et al. (2002). All other substances needed for basal reactions and cell proliferation are assumed to be available in a sufficient amount. In malnutrition states, meaning an undersupply of nutrients, the solid skeleton (cancer and regular cells) will undergo a necrotic process via ${\hat{\rho }}^{ST}_{\ominus ,\,IN}$ and ${\hat{\rho }}^{SB}_{\ominus ,\,IN}$. Right before this incident, the cancer cells start synthesising VEGF to initialise angiogenesis. In addition to this, the apoptosis reaction ${\hat{\rho }}^{ST}_{\ominus ,\,ID}$ based on the effect of the therapeutic agent is also considered resulting in a mass-loss term. Following this, the mass production term of the solid skeleton is found as

85 $$\begin{aligned} \hat{\rho }^{S} = \underbrace{\hat{\rho }^{ST}_{\oplus ,\,IN} \,-\, \hat{\rho }^{ST}_{\ominus ,\,IN} \,-\, \hat{\rho }^{SB}_{\ominus ,\,IN}}_{\hat{\rho }^{SB/ST}_{IN}} \,-\, \hat{\rho }^{ST}_{\ominus ,\,IV} \,-\, \hat{\rho }^{ST}_{\ominus ,\,ID} \,. \end{aligned}$$

where only ${{\hat{\rho }}}^{SB/ST}_{IN}$ exhibits gains and losses. Furthermore, all gain and loss processes are interacting with corresponding gains and losses of the interstitial fluid.

#### Mass production terms of the interstitial fluid

*Nutrients*: The change in nutrient mass is mainly governed by its consumption from metastatic and freely floating cancer cells through ${\hat{\rho }}^{IN}_{\ominus ,\,ST}$ and ${\hat{\rho }}^{IN}_{\ominus ,\,IC}$ as well as from healthy brain tissue through ${\hat{\rho }}^{IN}_{\ominus ,\,SB}$. The only increase is due to the triggering angiogenesis based on the VEGF concentration ${\hat{\rho }}^{IN}_{\oplus ,\,IV}$. Therefore, the overall nutrient mass changes via

86 $$\begin{aligned} \hat{\rho }^{IN} = \hat{\rho }^{IN}_{\oplus ,\,IV} \,-\, \hat{\rho }^{IN}_{\ominus ,\,IC} \underbrace{-\,\hat{\rho }^{IN}_{\ominus ,\,ST} \,-\, \hat{\rho }^{IN}_{\ominus ,\,SB}}_{\hat{\rho }^{IN}_{SB/ST}}\,. \end{aligned}$$

*Cancer cells*: Like the metastases, freely moving cancer cells proliferate through ${\hat{\rho }}^{IC}_{\oplus ,\,IN}$ due to the consumption of nutrients via ${\hat{\rho }}^{IN}_{\ominus ,\,IC}$. On the other hand, the lack of nutrients reduces the cancer cell mass by ${\hat{\rho }}^{IC}_{\ominus ,\,IN}$. Additionally, the interplay with the therapeutic agent leads to a mass loss governed by ${\hat{\rho }}^{IC}_{\ominus ,\,ID}$. Summing up, the mass production of the cancer cells results in

87 $$\begin{aligned} \begin{aligned} \hat{\rho }^{IC} = \underbrace{\hat{\rho }^{IC}_{{\oplus ,\,IN}} \,-\, \hat{\rho }^{IC}_{\ominus ,\,IN}}_{{\hat{\rho }}^{IC}_{IN}} \,-\, \hat{\rho }^{IC}_{\ominus ,\,ID}. \end{aligned} \end{aligned}$$

*VEGF*: The increase in nutrient consumption decreases the amount of nutrients in the interstitial fluid. This leads, firstly, to a stop in cancer cell proliferation and, secondly, to necrosis. Counteracting necrosis, VEGF is synthesised within the metastases and released into the interstitial fluid resulting in ${\hat{\rho }}^{IV}_{\oplus ,\,ST}$. Here, VEGF binds to the respective ligand side at the endothelial cells, thus triggering its growth in the direction of decreasing VEGF, cf. Hanahan and Weinberg (2011), Geiger and Peeper (2009) and Potente et al. (2011). However, the synthesis is much higher as its amount is consumed by the growth of endothelial cells with the result that there is a measurable amount of VEGF increase in the interstitial fluid and the blood circulation, cf. Werther et al. (2002) and Kut et al. (2007). This leads to

88 $$\begin{aligned} {\hat{\rho }}^{IV} = {\hat{\rho }}^{IV}_{\oplus ,\,ST}. \end{aligned}$$

*Therapeutic agent*: Metastases can only be discovered if their mass, respectively, their size, is above a certain threshold. Upon detection, a drug treatment can be initialised triggering apoptosis of cancer cells in the interstitial fluid through ${\hat{\rho }}^{ID}_{{\ominus ,\,IC}}$ and of metastases in the solid skeleton via ${\hat{\rho }}^{ID}_{\ominus ,\,ST}$. Furthermore, the therapeutic agent is reduced over time through ${\hat{\rho }}^{ID}_{\ominus ,\,IL}$ resulting in a half life of the drug of roughly 5 hours in case of a TRAIL^2^ derivate within mice, cf. Walczak et al. (1999). In the present study, the half life is considered to 5 h to compensate for the use and clearance of drugs as well as for its depletion into the blood vessels. Thus,

89 $$\begin{aligned} {\hat{\rho }}^{ID} = -{\hat{\rho }}^{ID}_{\ominus ,\,ST} \,-\, {\hat{\rho }}^{ID}_{\ominus ,\,IC}\,-\, {\hat{\rho }}^{ID}_{\ominus ,\,IL}\,. \end{aligned}$$

#### Results of the metastatic mechanism

Based on the above results, the individual production terms can be grouped together with their respective counterparts yielding

90 $$\begin{aligned}&{{\hat{\rho }}}^{SB/ST}_{IN}+{{\hat{\rho }}}^{IN}_{SB/ST}=0\,,\quad {{\hat{\rho }}}^{IN}_{IC}+{{\hat{\rho }}}^{IC}_{IN}=0\,,\nonumber \\&{{\hat{\rho }}}^{ST}_{ID}+{{\hat{\rho }}}^{ID}_{ST}=0\,,\quad {{\hat{\rho }}}^{IC}_{ID}+{{\hat{\rho }}}^{ID}_{IC}=0\,,\nonumber \\&{{\hat{\rho }}}^{ST}_{IV}+{{\hat{\rho }}}^{IV}_{ST}=0\,, \end{aligned}$$

where (84) has been used. The quantities ${{\hat{\rho }}}^{IN}_{\oplus ,\, IV}$ and ${{\hat{\rho }}}^{ID}_{\ominus ,\,IL}$ do not appear in (90) as ${{\hat{\rho }}}^{IN}_{IV}$ is based on the angiogenesis process and ${{\hat{\rho }}}^{ID}_{IL}$ is constantly depleting. Furthermore, it has been assumed that in addition to the general constraint of the mass productions, ${{\hat{\rho }}}^S+{{\hat{\rho }}}^I=0$, the particular mass productions together with their counterparts vanish. Based on this assumption, (83) and (90) result in

91 $$\begin{aligned}&{{\hat{\rho }}}^{SB/ST}_{IN}({{\bar{\mu }}}^{IN}-{{\bar{\mu }}}^S)\ge 0\,,\quad {{\hat{\rho }}}^{IC}_{IN}({{\bar{\mu }}}^{IN}-{{\bar{\mu }}}^{IC})\ge 0\,,\nonumber \\&{{\hat{\rho }}}^{ST}_{ID}({{\bar{\mu }}}^{ID}-{{\bar{\mu }}}^S)\ge 0\,,\quad {{\hat{\rho }}}^{IC}_{ID}({{\bar{\mu }}}^{ID}-{{\bar{\mu }}}^{IC})\ge 0\,, \nonumber \\&{{\hat{\rho }}}^{ST}_{IV}({{\bar{\mu }}}^{IV}-{{\bar{\mu }}}^S)\ge 0\,. \end{aligned}$$

Furthermore, the nutrient gain ${{\hat{\rho }}}^{IN}_{\oplus ,\,IV}$ and the drug loss ${{\hat{\rho }}}^{ID}_{\ominus ,\,IL}$ are only related to their specific chemical potentials ${\bar{\mu }}^{IN}$ and ${\bar{\mu }}^{ID}$, respectively, such that

92 $$\begin{aligned}&{{\hat{\rho }}}^{IN}_{\oplus ,\,IV}({{\bar{\mu }}}^{IN})\ge 0\,,\nonumber \\&{{\hat{\rho }}}^{ID}_{\ominus ,\,IL}({{\bar{\mu }}}^{ID})\ge 0\,. \end{aligned}$$

From a biological point of view, the nutrients would be released from the blood into the tissue of brain solid and interstitial fluid, cf. Abi-Saab et al. (2002). However, the model does not represent the blood as a mixture including interchangeable nutrients that would result in a dissipation relation similar to (91). Thus, the solid chemical potential is omitted in the dissipation inequality for the nutrient gain as it is not the primary interacting term. As a result, ${\bar{\mu }}^{IN}$ is assumed negative, thus allowing for a simplification of the model that ensures the biological process and allows, at the same time, for a positive constitutive ansatz function for ${{\hat{\rho }}}^{IN}_{IV}$. Similarly, the drug loss ${{\hat{\rho }}}^{ID}_{IL}$ is related to metabolisation as well as to depletion into the blood vessels. In the tissue, the metabolisation always results in smaller molecules increasing the dissipation. As a result, ${\bar{\mu }}^{ID}$ is assumed negative to allow for a positive ansatz function to equalise the negative sign of the mass reduction. Therewith, the dissipation inequality (83) is not only satisfied in a sufficient manner, but, at the same time, one gains a switch for the biological processes behind the particular productions. No matter, if the productions included in (91) are gains or losses, the corresponding process is only active when the particular inequality is fulfilled. For the gains and losses of the healthy brain material $\varphi ^{SB}$, for example, one obtains in dependence of nutrients $\varphi ^{IN}$

93 $$\begin{aligned} {{\hat{\rho }}}^{SB}_{\oplus ,\,IN}({{\bar{\mu }}}^{IN}-{{\bar{\mu }}}^S)\ge 0 \quad \text{ vs. }\quad {{\hat{\rho }}}^{SB}_{\ominus ,\,IN}({{\bar{\mu }}}^S-{{\bar{\mu }}}^{IN})\ge 0\,. \end{aligned}$$

For the gains and losses of all further mass productions, equivalent inequalities are obtained. This implies a threshold for the process and furthermore allows for a positive constitutive ansatz for the individual mass productions. Thereby, it is unnecessary to distinguish between gains and losses, as in case of losses, the order of chemical potentials in (91) changes.

#### Constitutive setting of mass production terms

*Solid skeleton*: For the description of the individual mass production terms of the solid, Monod-like kinetics are used for the proliferation in accordance with Ambrosi and Preziosi (2002), Shelton (2011) and Sciumè et al. (2013), whereas atrophy follows a linear behaviour. Following this, the growth and atrophy terms describing reactions to nutrient supply and drug medication can be modelled via

94 $$\begin{aligned} {\hat{\rho }}^{ST}_{{\oplus ,\,IN}} &= \displaystyle \,n^{ST}\rho ^{STR}\mathcal{H}_{1}\nu _{\oplus \,ST,\,{\mathrm{max}}}\, \frac{c^{{IN}}_{m}\,-\,{\bar{c}}^{{IN}}_{m}}{K_{gr}\, +\,(c^{IN}_{m}\,-\,{\bar{c}}^{IN}_{m})}\,,\nonumber \\ {\hat{\rho }}^{ST}_{{\ominus ,\,IN}} &= \displaystyle n^{ST}\rho ^{STR}\mathcal{H}_{2}\nu _{\ominus \,ST,\,{\mathrm{max}}}\,,\nonumber \\[1ex] {\hat{\rho }}^{ST}_{ID} &= \displaystyle \,n^{ST}\rho ^{STR}({\nu }_{\,ID,\,{\mathrm{max}}}\, \,+\,c^{{ID}}_{m} {\tilde{\nu }}_{\,ID,\,{\mathrm{max}}} )\,,\nonumber \\[1ex] {\hat{\rho }}^{SB}_{IN} &= \displaystyle n^{SB}\rho ^{SBR}\mathcal{H}_{3}\nu _{\ominus \,SB,\,{\mathrm{max}}}\,. \end{aligned}$$

Therein, $\nu _{\oplus \,ST,\,{\mathrm{max}}}$, $\nu _{\ominus \,ST,\,\mathrm{max}}$, $\nu _{\,ID,\,{\mathrm{max}}}$ and $\nu _{\ominus \,SB,\,{\mathrm{max}}}$ are maximum proliferation rates with the dimension $\mathrm 1/s$. ${\tilde{\nu }}_{\,ID,\,{\mathrm{max}}}$ is given in $\mathrm{m^3/(mol\cdot s)}$ and characterises the maximum reaction rate. Furthermore, $K_{gr}$ indicates the concentration of $c^{IN}_m$, at which half of the maximum proliferation rate $\nu _{\oplus \,ST,\,{\mathrm{max}}}$ is reached. Moreover, the mass production terms are only taken into account if their considered process is relevant. In case of the proliferation process, this appears when the cancer cells need to have a sufficient nutrient concentration ${\bar{c}}^{IN}_{m}$ available that is triggered by switching the Heaviside function $\mathcal{H}_{1}({\bar{c}}^{IN}_{m})$ from 0 to 1. Only above this concentration, ${\hat{\rho }}^{ST}_{{\oplus ,\,IN}}$ is taken into account. The same is true for the necrosis processes, where the Heaviside functions $\mathcal{H}_{2}({\tilde{c}}^{ID}_{m})$ and $\mathcal{H}_{3}(\breve{c}^{ID}_{m})$ initiate necrosis for nutrient concentrations below ${\tilde{c}}^{IN}_m$ or $\breve{c}^{IN}_m$, respectively.

*Interstitial fluid*: In contrast to the solid, where partial densities such as $\rho ^{ST}=n^{ST}\rho ^{STR}$ have been included, the mass productions of the interstitial fluid depend on molar concentrations and molar masses. Thus, partial densities such as $\rho ^{IC}$ are described via $\rho ^{IC}=n^{IC}\rho ^{ICR}=n^I\rho ^{IC}_I=n^Ic^{IC}_mM^{IC}_m$, cf. (12). As a result, one obtains for the growth and atrophy of cancer cells in analogy to (94) that

95 $$\begin{aligned} {\hat{\rho }}^{IC}_{\oplus ,\,IN} &= \displaystyle n^I c^{IC}_m\, M^{IC}_m\,\nu _{\oplus \,ST,\,{\mathrm{max}}}\, \frac{c^{{IN}}_{m}\,-\,{\bar{c}}^{{IN}}_{m}}{K_{gr}\, +\,(c^{IN}_{m}\,-\,{\bar{c}}^{IN}_{m})}\,,\nonumber \\ {\hat{\rho }}^{IC}_{\ominus ,\,IN} &= n^I c^{IC}_m\, M^{IC}_m\mathcal{H}_{2}\nu _{\ominus \,ST,\,{\mathrm{max}}}\,,\nonumber \\[1ex] {\hat{\rho }}^{IC}_{ID} &= \displaystyle n^I c^{IC}_m M^{IC}_m\, ({\nu }_{\,ID,\,{\mathrm{max}}}\, \,+\,c^{{ID}}_{m} {\tilde{\nu }}_{\,ID,\,{\mathrm{max}}})\,. \end{aligned}$$

When the nutrient concentration declines, this is a result of the nutrient demand of the cells for basal cell reactions sustained by ${\hat{\rho }}^{{IN}}_{\ominus ,\,\text {basal}}$, on the one hand, and for their proliferation guaranteed by ${\hat{\rho }}^{{IN}}_{\ominus ,\,\text {proli}}$, on the other hand. Thus,

96 $$\begin{aligned} {\hat{\rho }}^{{IN}}_{\ominus \,SB/ST} &= {\hat{\rho }}^{{IN}}_{\ominus ,\,\text {basal}} \,+\, {\hat{\rho }}^{{IN}}_{\ominus ,\,\text {proli}}\,,\nonumber \\ {\hat{\rho }}^{{IN}}_{\ominus ,\,\text {basal}} &= \displaystyle \,\nu _{IN,\,\text {basal}}\,M^{IN}_m N^{IC}\,(n^I{c^{IC}_{m}}M^{IC}_m \,+\, \,n^{ST}\rho ^{STR})\,, \nonumber \\ {\hat{\rho }}^{{IN}}_{\ominus ,\,\text {proli}} &= \displaystyle f_{\,\text {proli}} \,({\hat{\rho }}^{IC}_{\oplus ,\,IN} \,+\, {\hat{\rho }}^{ST}_{\oplus ,\,IN})\,. \end{aligned}$$

In (96)$_2$, $\nu _{IN,\,\text {basal}}$ is the maximum basal consumption rate of nutrients given in $\text {mol}/({\mathrm{cells\cdot s}})$. Furthermore, $\nu _{IN,\,\text {basal}}$ is based on the metabolic glucose uptake, cf. for example, Kallinowski et al. (1988), Vaupel et al. (1989), Choi et al. (2002) or Cherk et al. (2006). Moreover, $N^{IC}$ is the number of cells per unit mass, given in ${\mathrm{cells/kg}}$. In (96)$_3$, $f_{\,\text {proli}}$ is a constant factor multiplying the sum of production terms.

The remaining term within the nutrient production ${{\hat{\rho }}}^{IN}$ corresponds to the angiogenesis and the nutrient increase of the interstitial fluid. This term reads

97 $$\begin{aligned} {\hat{\rho }}^{{IN}}_{\oplus ,\,IC} = \displaystyle n^I M^{IN}_m\,\nu _{\oplus \,IV,\,{\mathrm{max}}}\, ({c}^{IN}_{0\,m}\,-\,c^{IN}_m)(n^B\,-\, n^B_0)\,. \end{aligned}$$

Therein, the nutrient increase is proportional to the maximum nutrient release rate $\nu _{\oplus \,IV,\,{\mathrm{max}}}$ corresponding to the blood volume fraction. Together with the nutrient concentration $c^{IN}_m$, its initial value $c^{IN}_{0\,m}$ and the molar mass $M^{IN}_m$ , it is responsible for the angiogenesis resulting from $n^B-n^B_0$.

Recapitulating, the increase in VEGF has been related to an increase in the blood saturation or the blood volume fraction, respectively, and finally to the nutrient concentration. Thus, the metastatic brain tumour triggers the angiogenesis process by the production and release of VEGF into the interstitial fluid. To simplify this process on the modelling side, it is assumed that the cancer cells generate VEGF according to the following equation:

98 $$\begin{aligned} {\hat{\rho }}^{IV}_{\oplus ,\,ST} = \displaystyle n^I M^{IV}_m \nu _{\oplus \,IV,\,{\mathrm{max}}}\,n^{ST} \frac{c^{IN}_{0\,m}\,-\,c^{IN}_m}{ c^{IN}_{0\,m}\,-\,c^{IN}_m \,+\,{\bar{c}}^{IN}_{m}} \frac{{\bar{c}}^{IV}_{m} - c^{IV}_m}{{\bar{c}}^{IV}_{m}}\,. \end{aligned}$$

The VEGF synthesis is initiated for nutrient concentrations below ${\tilde{c}}^{IN}_{m}$ and is proportional to the molar mass $M^{IV}_m$ of VEGF and the maximum rate $\nu _{\oplus \,IV,\,\mathrm{max}}$ given in $\mathrm {mol/(m^3\cdot s)}$.

However, the apoptosis is only activated if the threshold concentration ${\bar{c}}^{ID}_{m}$ of therapeutic drugs is reached and the Heaviside function $\mathcal{H}_{4}({\bar{c}}^{ID}_{m})$ switches from 0 to 1. This enables a tumour and cancer cell decline due to drugs, while the apoptosis reduces the amount of the therapeutic agents at the same time. As the drug binds to the receptor side on the cell surface, it does not contribute to any further apoptosis reactions. Following this, the mass of the therapeutic agent is reduced as follows:

99 $$\begin{aligned} {{\hat{\rho }}}^{ID}_{\ominus ,\,ST/IC} = \,\,\breve{\nu }_{ID,\,\mathrm max}\,\,\mathcal{H}_{4}( {\hat{\rho }}^{ST}_{ID} \,+\, {\hat{\rho }}^{IC}_{ID})\,-{\bar{\nu }}_{ID}c^{ID}_m M^{ID}_m\,. \end{aligned}$$

Therein, the two terms of (89) given in (94)$_3$ and (95)$_3$ have been summarised to yield ${{\hat{\rho }}}^{ID}_{\ominus ,\,ST/IC}$. Furthermore, the dimensionless factor $\breve{\nu }_{ID,\,{\mathrm{max}}}$ relates the mass loss of the cancer cells to the therapeutic agent, and ${\bar{\nu }}_{ID}$ corresponds to the half life of the drug.

#### The micrometastatic switch

During the cancer cell proliferation and the infiltration of cancer cells into the brain tissue, a volumetric growth occurs over time and can be expressed as a volume fraction after the growth has exceeded a certain critical cancer cell concentration. This situation characterises the so-called micrometastatic switch. As the cancer cell concentration is inherently not measurable by volume, there exists a related volume given by the volume fraction $n^{IC}$ mainly as a function of the cancer cell concentration $c^{IC}_m$.

In order to relate concentrations to volume fractions, recall that partial densities can be expressed by volume fractions and effective densities, on the one hand, and by molar concentrations and molar masses, on the other hand. For the species $\varphi ^{I\delta }$ of the interstitial fluid, this yields

100 $$\begin{aligned}&\rho ^{I\delta }=n^{I\delta }\rho ^{I\delta R}=n^I c^{I\delta }_mM^{I\delta }_m\,,\nonumber \\&\displaystyle \text{ such } \text{ that }\quad n^{I\delta }=\frac{n^I c^{I\delta }_mM^{I\delta }_m}{\rho ^{I\delta R}}\,, \end{aligned}$$

where (10) together with (12)$_1$ have been used.

In the avascular stage, cancer cells reach the brain as components of the interstitial fluid. Applying this to the general relation (100)$_2$ yields the volume fraction of metastatic cancer cells in the interstitial fluid of the brain as

101 $$\begin{aligned} n^{IC}=\frac{n^I c^{IC}_mM^{IC}_m}{\rho ^{ICR}}\,. \end{aligned}$$

During proliferation, the number of cells increases until a micrometastasis evolves, cf. Geiger and Peeper (2009). This situation is known as the micrometastatic switch defined by the critical cancer cell concentration ${\tilde{c}}^{IC}_m$. At this stage, the micrometastasis that has been floating in the interstitial fluid adheres to the solid skeleton, such that it is, on the one hand, a loss for the interstitial fluid and, on the other hand, a gain for the solid skeleton. Thus,

102 $$\begin{aligned} n^{IC}=n^{ST}=\frac{n^I {\tilde{c}}^{IC}_mM^{IC}_m}{\rho ^{STR}} \longrightarrow {\left\{ \begin{array}{ll} ~n^I=\sum _\gamma \,n^{I\gamma }-n^{IC}\\ ~n^S=n^{SB}+n^{ST}, \end{array}\right. } \end{aligned}$$

with $\rho ^{ICR}=\rho ^{STR}=\rho ^{SR}$. Typically, micrometastases are characterised by a specific diameter of approximately 2 mm which can be related to a number of roughly 1.3 million cells, cf. Hermanek et al. (1999). This yields a cancer cell concentration threshold of ${\tilde{c}}^{IC}_m = 1.7\times 10^{-16}~\text{ mol/m}^3$. Thus, once this concentration is exceeded through

103 $$\begin{aligned} \displaystyle c^{IC}_m~>~{\tilde{c}}{}^{IC}_m\,, \end{aligned}$$

a micrometastasis has emerged.

Following this, the micrometastatic switch from the threshold cancer cell concentration ${\tilde{c}}^{IC}_{m}$ to the initiation of the metastatic volume fraction $n^{ST}_0$ at $t=t_{ST}\ge t_0$ is governed by

104 $$\begin{aligned} \displaystyle n^{ST}_0 = \displaystyle \frac{n^{I} {\tilde{c}}^{IC}_{m} M^{IC}_{m}}{\rho ^{STR}}\,\text {.} \end{aligned}$$

Further growth is contributed to the metastatic volume fraction $n^{ST}$ via ${\hat{n}}^{ST}$.

## Weak form of the governing equations describing the finite-element analysis of growth and atrophy of brain metastases

Targeting the use of the finite-element analysis (FEA) for the numerical computation of proliferation and atrophy processes, the basic governing equations have to be transformed into their weak forms. As the problem is generally highly coupled through the simultaneous action of the overall momentum balance, the fluid mass balances and the concentration balances of the interstitial fluid species, a fully coupled algorithm is strove for, where the system of weak equations is solved simultaneously within the Bubnov–Galerkin method.

Starting with the overall momentum balance (21) tested by $\delta {\mathbf{u}}_S$, the corresponding weak form reads with the aid of (23)

105 $$\begin{aligned} \displaystyle G_{u_S}= & {} \displaystyle \int _\varOmega \,\sum _{\alpha }{{\mathbf {T}}}^{\alpha }\cdot {{{\mathrm{grad}}}}\,\delta {{\mathbf {u}}}_{S}\,\mathrm {d}v\,-\,\displaystyle \int _\varOmega \,\rho \,{\mathbf {g}}\cdot \delta {{\mathbf {u}}}_{S} \,\mathrm {d}v\nonumber \\&-\, \displaystyle \int _{\varOmega } \,{\hat{\rho }}^I\mathbf{w}_I\cdot \delta {{\mathbf {u}}}_{S}\,\mathrm {d}v\,-\, \int _{\Gamma }\, \bar{\mathbf{t}} \cdot \delta {{\mathbf {u}}}_{S}\,{{\mathrm{d}}}{a} = 0\,\text {,} \end{aligned}$$

where $\bar{\mathbf{t}} = \sum _{\alpha }{\mathbf{T}}^\alpha {\mathbf{n}}$ is the total external load vector acting at the Neumann boundary with outward-oriented unit normal vector $\mathbf{n}$, while $\varOmega$ and $\Gamma$ are the domain and the surface of the solid skeleton under study.

On the basis of (15)$_2$, the volume balances of the blood and the overall interstitial fluid are tested by $\delta p^{\beta R}$ and yield in their weak forms

106 $$\begin{aligned} \displaystyle G_{p^{BR}}= & {} \displaystyle \int _\varOmega \Big [ (n^B)^\prime _S \,+\, n^B \text{div}\, ({{\mathbf {u}}}_{S})^\prime _S\Big ] \,\delta p^{BR} \, \mathrm {d}v\nonumber \\&-\, \displaystyle \int _\varOmega n^B {\mathbf{w}}_B \cdot {{{\mathrm{grad}}}}\,\delta p^{BR}\, \mathrm {d}v\,+\,\displaystyle \int _\Gamma {\overline{v}}^B \,\delta p^{BR} \,\mathrm {d}a= 0 \,,\nonumber \\ \displaystyle G_{p^{IR}}= & {} \displaystyle \int _\varOmega \Big [ (n^I)^\prime _S \,+\, n^I \text{div}\, ({{\mathbf {u}}}_{S})^\prime _S \,-\,{\hat{n}}^I\Big ] \,\delta p^{IR} \, \mathrm {d}v\nonumber \\&- \,\displaystyle \int _\varOmega n^I {\mathbf{w}}_I \cdot {{{\mathrm{grad}}}}\,\delta p^{IR}\, \mathrm {d}v\,+\,\displaystyle \int _\Gamma {\overline{v}}^I \,\delta p^{IR} \,\mathrm {d}a= 0 \,, \end{aligned}$$

where ${\overline{v}}^\beta = n^{\beta }{\mathbf {w}}_{\beta }\cdot {\mathbf {n}}$ are the volumetric effluxes of the pore fluids $\varphi ^\beta$.

Finally, the relevant dissolved species $\varphi ^{I\delta }$ of the interstitial fluid mixture, cancer cells, nutrients and drugs, are described by the weak formulations of their concentration balances (20) tested by $\delta c^{I\delta }_m$ yielding

107 $$\begin{aligned} G_{c^{I\delta }_m}= & {} \displaystyle \int _\varOmega \Big [n^I(c^{I \delta }_m)^\prime _S\,+\,c^{I\delta }_m\,\text{div}\, ({{\mathbf {u}}}_{S})^\prime _S\,+\,c^{I \delta }_m\,\text{div}\, (n^{B}{{\mathbf {w}}}_B)\nonumber \\&\displaystyle -\,n^I c^{I\delta }_m {\mathbf{w}}_{\delta I} \cdot {{{\mathrm{grad}}}}\,\delta c^{I \delta }_m\displaystyle \,-\,c^{I\delta }_m\Big (\frac{{\hat{\rho }}^S}{\rho ^{SR}}+ \frac{{\hat{\rho }}^{ I\delta }}{\rho ^{I\delta }_I}\Big )\,\Big ]\,\delta c^{I\delta }_m\,\mathrm {d}v\nonumber \\&\displaystyle \displaystyle +\,\int _\Gamma {{\bar{\iota }}}^{I\delta }\,\delta c^{I\delta }_m\,\mathrm {d}a= 0\,. \end{aligned}$$

Therein, ${{\bar{\iota }}}^{I\delta } = n^Ic^{I\delta }_m\,\mathbf{w}_{\delta I}\cdot {\mathbf {n}}$ is the molar efflux of $\varphi ^{I\delta }$ across the Neumann boundary. As the problem under study is growth-dependent, such that $n^S$ cannot be found from $n^S_0$ and $\text{ det }\,{\mathbf{F}}_S$ alone, an additional equation is necessary to obtain $n^S$. This term can either be found by combination of (6) and (17) yielding

108 $$\begin{aligned} n^S=\displaystyle \,n^S_{0}\,\exp \,\Big (\, \int ^t_{t_{ST}} \frac{{\hat{\rho }}^S}{\rho ^S} \,{\mathrm{d}}{\tilde{t}} \,\Big )\,(\det \mathbf{F}_S)^{-1}\, \end{aligned}$$

or by the use of the weak form of the solid volume balance (6) tested by $\delta n^S$, such that

109 $$\begin{aligned} G_{n^S}= & {} \int _\varOmega \big [(n^S)^\prime _S-\frac{{{\hat{\rho }}}^S}{\rho ^{SR}}-(\mathbf{u}_S)^\prime _S\,\cdot \,\text{ grad }\,n^S\,\big ]\,\delta n^S\,{\mathrm{d}}v\nonumber \\&-\,\int _\varOmega n^S({\mathbf{u}}_S)^\prime _S\,\cdot \,\text{ grad }\,\delta n^S\,{\mathrm{d}}v+\int _\Gamma {\bar{v}}^S\,\delta n^S\,{\mathrm{d}}a = 0\,. \end{aligned}$$

Therein, ${\bar{v}}^S=n^S({\mathbf{u}}_S)^\prime _S\,\cdot \,{\mathbf{n}}=0$. This means that in a Lagrangian setting, no solid volume flux across the surface of $\varphi ^S$ is possible.

Commenting on the choice of using either (108) or (109) in a numerical study, one must be aware that (108) can only be taken when ${\hat{\rho }}^S$ is given in an integrable form. Otherwise, one has to proceed with (109) instead.

The above equations are sufficient to compute the primary variables of the model. These are the solid displacement ${\mathbf{u}}_S$, the effective pressures $p^{BR}$ and $p^{IR}$ of the blood and the interstitial fluid mixture and the relevant concentrations $c^{I\delta }_m$ of the considered interstitial fluid species, while the solid volume fraction, as a result of the integrability of ${\hat{\rho }}^S$, is based on (108) and is therefore a secondary variable. The remaining secondary variables, such as the stresses and the momentum productions, can be obtained as functions of these terms, cf. Ehlers (2009) and Ehlers and Wagner (2015).

The spatial solution of the overall model makes use of the solver PANDAS and is based on the extended Taylor–Hood elements with quadratic approximation functions for the solid displacement $\mathbf{u}_S$ and linear approximation functions for the remainder of primary variables. In the time domain, we proceed from an implicit Euler scheme.

## Parameter identification and optimisation based on experimental data

### Parameter identification and optimisation

Due to the large number of simulation input parameters in the governing balance equations of the overall model, a great uncertainty is found for the quantitative description of the simulated processes. This problem can partially be overcome with the aid of raw data directly obtained from experimentalists, and a subsequent identification and optimisation of the most relevant model parameters. In this procedure, a first step is to show what parameters are sensitive for the currently considered process, while, in a second step, the most crucial parameters are optimised. In this study, we rely on cancer cell proliferation and on apoptosis-triggering medication experiments that have been conducted in vitro on cancer cell cultures.

The following parameter identification process is based on the parameter sampling method originating from Morris (1991) together with sensitivity measures introduced by Campolongo et al. (2007). In this context, the deviation between the model and the data is denoted as the model output *y*. The dependency of *y* on each parameter is evaluated using the elementary effects method introduced by Morris (1991) and improved in Campolongo et al. (2007). This method is preferred to measures that make use of the variance of a model as were proposed, for example, by Sobol (1993) or by Saltelli (2002). The variance-based measure requires a high amount of model executions in the sense of Monte Carlo samples that is not affordable in a reasonable time for the model under discussion. On the contrary, the Morris method only requires a small number of executions, which are related to the number of tested parameters and the introduced step size for the parameter modification.

The fundamental idea is the definition of an elementary effect

110 $$\begin{aligned}&EE(p_i) = \frac{y_1(p_1,\ldots ,p_{i-1},\,p_i+\varDelta ,\,p_{i+1},\ldots ,p_k)\,-\,y_2(P)}{\varDelta }\nonumber \\&\text{ with }\quad P=(p_1,\ldots ,\,p_k) \end{aligned}$$

describing the change of the model output *y* by modifying one of the *k* parameters $p_i$ with a specific step size $\varDelta$. Then, the two model outputs $y_1$ and $y_2$ are compared to each other. The specific step size $\varDelta =1/(l-1)$ depends on the number of parameter changes *l*, called levels. Thus, the parameters $p_i$ are usually not in a normalised interval between the normalised lower boundary $nlb=0$ and the normalised upper boundary $nub=1$. As a result, $\varDelta$ has to be transferred to the parameter space defined between the lower and upper boundaries $[lb_i,ub_i]$.

The overall method is based on changing one parameter at a time in such a way, that only a minimum of model runs has to be performed and that the parameter space is sampled efficiently. Therefore, a trajectory *r* within the parameter space is constructed by randomly selecting initial normalised parameter values ${\bar{p}}_i$ which range from *nlb* to $nub-\varDelta$. Based on this parameter set, one of the parameters ${\bar{p}}_i$ is then randomly selected and increased by $\varDelta$. The whole newly created parameter set itself is then the basis for an increase in another parameter. Further on, this process is repeated until all parameters have changed, cf. Morris (1991) and King and Perera (2013). This results in $k+1$ parameter sets. To end up with a sufficient number of elementary effects for a single parameter, $r=10$ trajectories are selected resulting in $r(k+1)$ model evaluations. This is still feasible with regard to the simplified models that are used to identify the crucial parameters. The final quantity of interest in determining the importance of a parameter is achieved by the absolute mean $\mu ^*_i$ of all *r* absolute elementary effects of a parameter $p_i$ yielding

111 $$\begin{aligned} \mu ^*_i = \sum _i \mid {EE(p_i)}\mid /r\,, \end{aligned}$$

cf. Campolongo et al. (2007) and King and Perera (2013). This mean or sensitivity, respectively, allows for a comparison of $p_i$ with the other parameters, where the highest values are considered significant, cf. King and Perera (2013). However, this method only allows for an alignment of the relevant parameters, while the decision on what parameters are relevant for the model under discussion has still to be made by the user of the method.

Furthermore, the parameter optimisation strategy is based on maximum likelihood estimations with the goal to determine the model parameters from experimental data, cf. Myung (2003). This is done by relating simulation results $y_\text {sim}$ to the measurement data $y_\text {data}$ on the basis of a logarithmic error $\log \epsilon = \log (y_\text {dat})\, -\,\log (y_\text {sim})$. This error is assumed to be Gaussian distributed resulting in the likelihood function

112 $$\begin{aligned} {\mathscr {L}} = \displaystyle \sum _i \log \Big (\frac{1}{\sigma _i\sqrt{2\pi }} \Big )\,-\,\frac{(y_{\text {dat},\,i} -y_{\text {sim},\,i})^2}{2\sigma ^2_i}\,. \end{aligned}$$

Therein, $\sigma _i$ is the variance and $\mathscr {L}$ the summed likelihood function including both the experimental and the simulated data at point *i*. In order to optimise the crucial model parameters, the likelihood function $\mathscr {L}$ is minimised within the commercial software Matlab^3^ and especially within its optimiser fmincon.

For the parameter identification process, two basic experimental procedures are studied, growth by proliferation of cancer cells and atrophy by apoptosis through medical drugs.

### Study of the proliferation process

The following section focuses on one of the main cancer-related processes, the proliferation of cancer cells under sufficient nutrient supply. The corresponding experiments have been carried out by people of the group around Professor Morrison, while the experimental results and data are part of the dissertation thesis of Daniela Stöhr (Stöhr 2018) and a following article (Stöhr et al. 2020).

Despite the numerical model that is able to describe proliferation and atrophy of cancer cells, the model parameters related to these processes have still to be identified and determined. Therefore, the above sensitivity analysis is applied resulting in a model output *y* composed of the likelihood ${{{\mathscr {L}}}}$ of the error $\varepsilon$ between the experimental data and the simulation results. Therewith, the relevant parameters of the proliferation model can be identified and an optimal parameter set can be determined. As a result, the adaptation to the experimental data is obtained.

#### Proliferation experiment and data

**Fig. 6:**
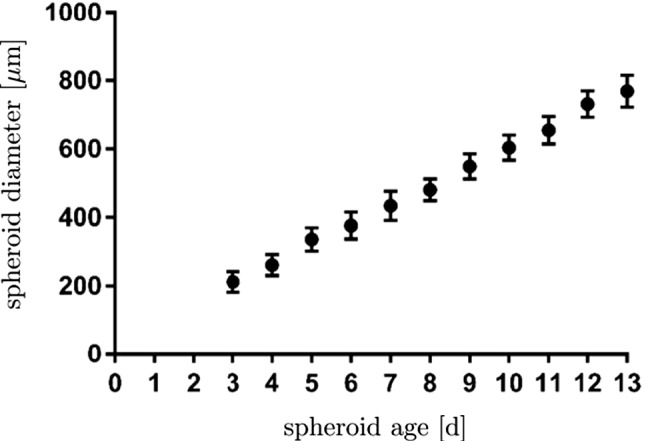
NCI-H460 spheroids cultivated in agarose-coated 96-well plates with the media changed every third day. Pictures were taken with an EVOS inverted digital microscope, and the diameter was determined using the Fiji software. Displayed are mean values ± SD of 51 spheroids from four independent experiments after Stöhr (2018)

**Fig. 7:**
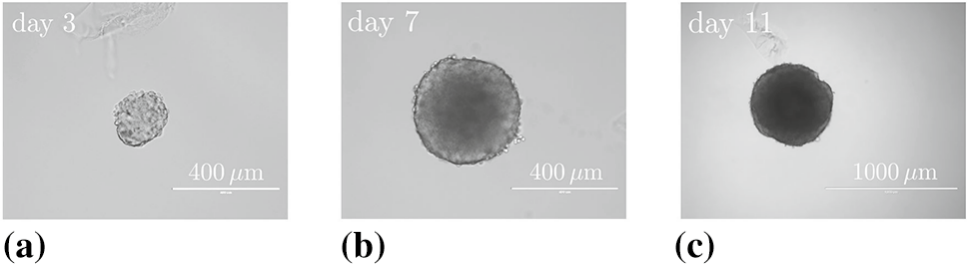
Images of the growing tumour cluster at days 3, 7 and 11 after Stöhr (2018)

In this study, the human lung cancer cell line NCI-H460 was used that was obtained from the American Type Culture Collection (LGC Standards GmbH, $\#\mathrm{HTB-}177$). The cells were maintained in RPMI 1640 medium (Life Technologies, Gibco, Karlsruhe, Germany) supplemented with 5% foetal calf serum at $37^\circ$ C in a humidified incubator with 5% $\mathrm{CO}_2$. The experimental procedure was as follows. Firstly, one hundred cancer cells were seeded onto Terasaki multiwell plates for three days and were then transferred onto agarose-coated well plates. Thereon, the cancer cells stuck together and formed a cancer cell spheroid, cf. Figs. 6 and 7. The proliferation of the spheroid was recorded by an inverted digital microscope (EVOS FL Imaging System, Thermo Fisher Scientific, Waltham, MA, USA) and was analysed with the image-editing program Fiji, cf. Schindelin et al. (2012), thus taking the values of the cross-sectional area and the corresponding volume. In a post-processing step, the volume was converted into cell numbers, where the assumption was made that the single cells are spherical occupying a volume of $3.053\times 10^{-15}\,\text {m}^3$ per cell with no voids in between them. By division of the whole spheroid volume, the amount of cells was obtained. During the experiments, four different sets of twice 11 and once 14, respectively, 15 spheroids have been measured for 13 days. The volume, respectively, the cell numbers, have been summed up and the mean volume $m^{\,IC}_{\, {\mathrm{vol}}}$ and the corresponding standard deviation $std^{\,IC}_{\,{\mathrm{vol}}}$ have been calculated. $N_{Sp}$ indicates the number of spheroids that have been used for the measurement at the respective day, cf. Table 1. Note that not all spheroids have been measured each day leading to varying spheroid numbers $N_{Sp}$.

**Table 1:** Cancer-cell proliferation data

Time in $[\,\text {d}\,]$	${m}^{\,IC}_{\,\text {vol}}$ in $[\text {mm}^3]$	${std}^{\,IC}_{\,\text {vol}}$	$N_{Sp}$ in [–]
3	0.0052126	0.0023879	50
4	0.0092883	0.0036419	50
5	0.019935	0.0062061	26
6	0.025853	0.0092565	51
7	0.04252	0.01313	37
8	0.058203	0.011653	24
9	0.086102	0.017856	49
10	0.11521	0.021385	44
11	0.14935	0.027576	34
12	0.20214	0.054374	14
13	0.23523	0.044431	22

#### Numerical simulation of the proliferation experiment

*Model*: In accordance with the experiment, the numerical model aims at determining the proliferation of cancer cells during the time interval of the experiment resulting in a low cancer cell mass as well as volume.

Despite the experimental set-up, where the spheroids are dissolved in a solution, the corresponding numerical simulation treats the spheroid in a simplified 2-d approach. In particular, the spheroid is described here as a certain amount of cancer cells dissolved in an interstitial fluid solution as described in Sect. 2.2.

The basic set-up includes the brain tissue as the solid $\varphi ^{S}$ together with the interstitial fluid solution $\varphi ^{I}$ with dissolved nutrients $\varphi ^{IN}$ and cancer cells $\varphi ^{IC}$. Although the blood does not play any role in the proliferation model, the blood is not omitted in this study in order to maintain similarities with the complex overall model and to enable a more reliable transfer between the models. However, $\varphi ^B$ is included in a simplified manner by the use of a constant volume fraction $n^B=s^Bn^P$ instead of the angiogenesis formulation based on (67). This leads to a reduced set of governing equations that, in their weak forms, are the overall momentum balance (105), where the gravitational term has been neglected, the volume balance (106) of the interstitial fluid, and the concentration balances (107) of nutrients and cancer cells. This system of coupled equations is solved for the primary variables solid displacement ${\mathbf{u}}_{S}$, blood and liquid pore pressures, $p^{BR}$ and $p^{IR}$, and nutrient and cancer concentrations, $c^{IN}_{m}$ and $c^{IC}_{m}$, while the solid volume fraction is obtained from (108).

Finally, the mass interactions (mass production terms) reduce to the proliferation of cancer cells (95)$_{1}$ and the nutrient consumption (96).

**Fig. 8:**
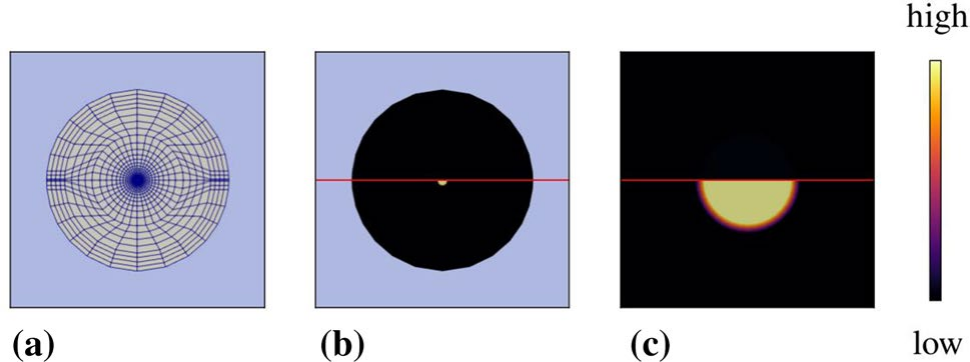
Numerical results corresponding to the proliferation experiments. **a** Circular domain (FE mesh) of interest with a diameter of 0.2 m. **b**, **c** Initial situation (top) and final situation (bottom) of an initial cancer cell cluster with a diameter of 0.0025 m and a cancer cell concentration of $c^{IC}_{m}=1.0\times 10^{-15}\,\text {mol}/\text {m}^{3}$ that spreads towards a final diameter of 0.0108 m at a cancer cell concentration of $c^{IC}_{m}=4.45\,\cdot \,10^{-14}\,\text {mol}/\text {m}^{3}$. **c** Zoomed-in situation of **b**

*Parameter identification and optimisation*: The goal of this proliferation study is the parameter identification and optimisation of the most sensitive proliferation input parameters. The first step towards this goal is the creation of the model domain. This is handled by the use of the software “Cubit” and results in a 2-d circular finite element (FE) mesh with a diameter of $0.2\,{\mathrm{m}}$ subdivided in 954 Taylor–Hood elements with 2899 nodes, cf. Fig. 8a. The boundary and initial conditions are such that the solid displacements are set to zero along the boundary line, while the external pressures are set to ${{\bar{p}}}^{BR}_0=1725\,{\mathrm{Pa}}$ and ${{\bar{p}}}^{IR}_0=258\,{\mathrm{Pa}}$ which, in turn, basically implies a free-flow condition across the boundary for the blood and the interstitial fluid. Furthermore, the nutrient concentration at the boundary is set to $c^{IN}_m={\mathrm{1\,mol/m^3}}$, while the same values of pressures and of nutrient concentration are assumed to be initially distributed in the whole domain. This ensures a sufficient nutrient supply as has been used in the experimental set-up. Furthermore, the cancer cell concentration is restricted to stay within the domain, which is satisfied by the use of a no-flow boundary condition. Initially, the cancer cells occupy the middle of the domain within a radius of 0.0025 m with a concentration of $c^{IC}_{m}=10^{-15}\,\text {mol}/\text {m}^{3}$. This corresponds to a similar amount of cancer cells as in the experiment. Over time, the cancer cells slightly migrate throughout the domain, although the proliferation is mainly concentrated in the middle of the domain, as can be seen from the concentration difference between the beginning and end of the simulation, cf. Fig. 8b, c.

The proliferation model is governed by a lot of input data such as material and model parameters that have a certain sensitivity on the numerical problem under study, cf. Table 2. Therein, the most sensitive parameters are included in the order of decreasing values of the mean $\mu ^*_i$ of their elementary effect. As the values of $\mu ^*_i$ range across 14 orders of magnitude, a horizontal line has been included beneath the most important ones. These are the maximum proliferation rate $\nu _{\oplus \,ST,\,{\mathrm{max}}}$, the amount $N^{IC}$ of cancer cells per unit mass, the molar cancer cell mass $M^{IC}_m$, the constant $K_{gr}$ indicating half of the maximum of nutrient concentration, and the threshold nutrient concentration ${{\bar{c}}}^{IN}_m$ necessary for proliferation. Parameters that are not in the list do not play a measurable role in this context.

**Table 2:** Sensitivities of proliferation parameters ranked from high to low values in accordance with the absolute mean $\mu ^{*}_i$ of their elementary effect

Parameter	$\mu ^{*}_i$
$\nu _{\oplus \,ST,\,{\mathrm{max}}}$	$1.87\times 10^{4}$
$N^{IC}$	$5.02\times 10^{3}$
$M^{IC}_m$	$2.02\times 10^{3}$
$K_{gr}$	$1.35\times 10^{3}$
${\bar{c}}^{IN}_{m}$	$8.45\times 10^{2}$
$D^{IC}$	1.66
$N^{IC}$	0.03
$K^{SI}$	$3.33\times 10^{-8}$
$\lambda ^S_0$	$2.3\times 10^{-9}$
$\mu ^S_0$	$2.01\times 10^{-9}$
$\mu ^{IR}$	$9.9\times 10^{-10}$

Parameters with vanishing $\mu ^{*}_i$ are omitted in the table

**Table 3:** Default parameters of the proliferation and apoptosis models

	Value	Unit	References
$\rho ^{SR}$	1190.0	$[{\mathrm{kg/m}}^{3}]$	Based on 1.5 kg (Azevedo et al. 2009) and $1.26\times 10^{-3}\,{\mathrm{m}}^3$ volume (Cosgrove et al. 2007)
$\rho ^{BR}$	1055.0	$[{\mathrm{kg/m}}^{3}]$	In the range of $1060\,{\mathrm{kg/m}^3}$ (White et al. 1992)
$\rho ^{IR}$	993.3	$[{{\mathrm{kg/m}}}^{3}]$	According to the density of water at $37^{\circ }\hbox {C}$ (Vanoni 2006)
$\mu ^{S}_{0}$	662.0	$[{\mathrm{Pa}}]$	Based on Young’s modulus for white matter (Budday et al. 2015) and Poisson’s ratio of $\nu ^S_0=0.42$
$\lambda ^{S}_{0}$	3312.0	$[{\mathrm{Pa}}]$	Based on Young’s modulus for white matter (Budday et al. 2015) and Poisson’s ratio of $\nu ^S_0=0.42$
$n^{B}_{0}$	0.05	[ – ]	Nicholson (2001)
$n^{I}_{0}$	0.20	[ – ]	Nicholson (2001)
$n^S_0$	0.75	[ – ]	$n^S_0=1-n^P_0$ with $n^P_0=n^B_0+n^I_0$
$\mu ^{BR}$	$3.5\times 10^{-3}$	$[{\mathrm{Pa}}\, \mathrm{s}]$	Rounded at a shear rate of $0.7\,\text {s}^{-1}$ (Windberger et al. 2003)
$\mu ^{IR}$	$0.7\times 10^{-3}$	$[{\mathrm{Pa}}\, {\mathrm{s}}]$	According to water at $37^{\circ }$ C (Huber et al. 2009)
$k^{B}$	$5.0\times 10^{-8}$	$[{\mathrm{m/s}}]$	Su and Payne (2009)
$k^{I}$	$5.0\times 10^{-13}$	$[{\mathrm{m/s}}]$	Kaczmarek et al. (1997)
$D^{ID}$	$5.0\times 10^{-9}$	$[{\mathrm{m^2/s}}]$	Chosen between $M^{ID}_m=93\,{\mathrm{kg/mol}}$ (Young et al. 1980) and $M^{ID}_m=62\,{\mathrm{kg/mol}}$ (Peters 1984)
$D^{IN}$	$6.6\times 10^{-10}$	$[{\mathrm{m^2/s}}]$	Molar diffusion coefficient for glucose (Hober et al. 1946)
$D^{IV}$	$4.0\times 10^{-11}$	$[{\mathrm{m^2/s}}]$	Anderson and Chaplain (1998)
$M^{IC}_m$	$1.3\times 10^{13}$	$[{\mathrm{kg/mol}}]$	Chosen based on Freitas (1999) and Da Silva and Williams (2001)
$M^{IN}_m$	0.18	$[{\mathrm{kg/mol}}]$	Molar mass of glucose
$M^{ID}_m$	93	$[{\mathrm{kg/mol}}]$	Siegemund et al. (2012) for $\hbox {Db}_{\alpha \text {EGFR}}$-scTRAIL
$M^{IV}_m$	50	$[{\mathrm{kg/mol}}]$	Ferrara et al. (2003)
$N^{IC}$	$10^{11}$	$[{\mathrm{cells/kg}}]$	Del Monte (2009)

While all input data of Table 3 are taken from the literature, the most important proliferation data of Table 2 have been optimised by the use of the Morris method, cf. Sect. 5.1. The optimisation was performed for 25 varied starting parameter sets to disturb the maximum-likelihood method in order to find global or at least a variety of different local minima. The best optimised parameter set corresponds to the highest likelihood and is shown in Table 2. As the molar mass $M^{IC}_m$ and the cell amount $N^{IC}$ are reliable quantities, the parameter optimisation basically concerns $\nu _{\oplus \,ST,\,{\mathrm{max}}}$, $K_{gr}$ and ${\bar{c}}^{IN}_m$, cf. Table 4.

Note that $K^{SI}$ of Table 2 is the intrinsic permeability of the brain with respect to the interstitial fluid compartment given in ${\mathrm{m}}^2$, whereas $k^I$ in Table 3 is the corresponding hydraulic conductivity given in ${\mathrm{m/s}}$, also compare (49). The same applies to the intrinsic and hydraulic conductivities $K^{SB}$ and $k^B$ of the blood.

**Table 4:** Optimised proliferation parameters

	Value	Unit
$\nu _{\oplus \,ST,\,{\max }}$	$4.15\times 10^{-6}$	$[\,1/ \text {s}\,]$
$K_{gr}$	0.156	$[\,\text {mol} /\text {m}^{3}\,]$
${\bar{c}}^{IN}_{m}$	0.35	$[\,\text {mol} /\text {m}^{3}\,]$

**Fig. 9:**
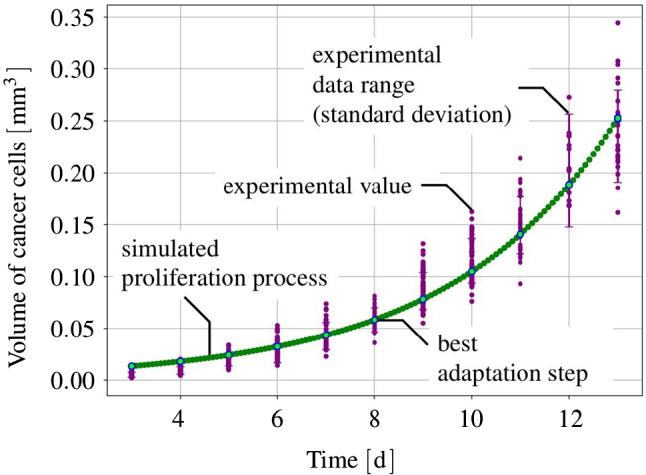
Proliferation cancer cell data and optimised simulation results. Volume of cancer cells from the experiment is depicted in violet. The simulation results are given in blue including the best result. The numerical simulation with the optimised parameters is given in green

Comparing the optimisation results with the experimental data as depicted in Fig. 9 reveals, on the one hand, the wide distribution of the single spheroid volume (indicated by purple points) and, on the other hand, the close match of the simulation results (in sky blue) that lay within the standard deviation of the experiment results (purple bars). The results at the individual time steps (in green) illustrate that the time progression follows an exponential function as in the beginning of the cancer cell growth, cf. Laird (1965) and Freyer and Sutherland (1986), as well as in bacterial cell cultures, cf. Ram et al. (2019), and in yeast, cf. Slavov et al. (2014). A direct comparison of the experimental and the numerical results, cf. Figs. 6 and 9 together with Table 1, exhibits, for example, for day 12, that the optimised curve of Fig. 9 yields a cancer cell volume of $0.19\,{\mathrm{mm}}^3$ compared to a volume of $0.20\,{\mathrm{mm}}^3$ given in Table 1.

### Study of the apoptosis process

This section focuses on the second main cancer-related process, the cancer cell death triggered by a death-receptor agonist. Thereby, the apoptosis experiments of cancer cell cultures are presented, and the resulting survival–cancer cell ratios are shown. Again, the Morris method is applied in order to find parameter sensitivities and to optimise the most important model parameters. Thus, the significant parameters are adapted to the data and the resulting parameter set as well as a numerical visualisation are given.

#### Apoptosis experiment and data

The cancer cell survivability in solutions with different concentrations of the therapeutic agent TRAIL, a tumour necrosis factor-related apoptosis-inducing ligand or, more precisely, $\hbox {Db}_{\alpha \text {EGFR}}$-scTRAIL, has been experimentally determined using cell staining and flow cytometry techniques, where the material and methods of this experimental set-up are presented in Stöhr (2018) and in Stöhr et al. (2020). In the following, a short summary is given to illustrate the set-up. The cancer cells under consideration are again human lung cancer carcinoma cells NCI-H460 that have been grown with 5% foetal calf serum in 2-d cell cultures. The treatment with TRAIL induces apoptosis in the cells by binding to tumour necrosis factor-related apoptosis-inducing ligand receptors (TRAILR1 and TRAILR2) on the cell surface. In the process of apoptosis, the lipid phosphatidylserine flips from the inside to the outside of the cell membrane, thus allowing staining with Annexin V-EGFP, an enhanced green-fluorescent protein, whose emitted fluorescence can be analysed by the flow cytometry technique. As a result, the percentage of viable cells can be calculated by comparing the number of not stained cells with the overall cell number in a treated cell culture.

During the experiments, the cells have been treated for 24 h with TRAIL resulting in the data shown in Table 5.

**Table 5:** Ratio of viable cancer cells at TRAIL concentrations of $c^{ID}_m~\in \,[\,10^{-10},\,10^{-9},\,10^{-8},\,10^{-7},\,10^{-6}\,]$ given in mol/$\hbox {m}^3$

$c^{ID}_{m}$ in $[\,{{\mathrm{mol}}}/{\mathrm{m}}^3\,]$	${m}^{IC}$ in [%]	${std}^{\,IC}$ in [–]
$10^{-10}$	87.07	7.15
$10^{-9}$	75.95	7.30
$10^{-8}$	55.50	10.41
$10^{-7}$	20.37	14.33
$10^{-6}$	3.36	1.83

Mean value ${m}^{IC}$ and standard deviation ${std}^{\,IC}$ of viable NCI-460 cells after stimulation with TRAIL for 24 h

#### Numerical simulation of the apoptosis experiment

*Model*: With regard to the numerical simulation of the apoptosis process, the overall model is simplified to include only the relevant processes concerning the cell death via TRAIL. The relatively fast cell death during the laboratory experiments allows for major simplifications of the complex overall model. However, despite the experimental set-up of cancer cells bathing within a solution on a plate, the continuum mechanical model is, as for the proliferation experiment before, still related to an initial metastasis $\varphi ^{ST}$ within brain tissue $\varphi ^S$. The metastasis is supplied via nutrients $\varphi ^{IN}$ existing as solutes in the interstitial fluid $\varphi ^{I}$. Furthermore, the medication in both the experimental and the numerical set-up is applied to the solution that is distributed in the whole interstitial fluid domain. This results in an immediate interaction with the cancer cells or the metastasis, respectively.

To trigger apoptosis, the drug TRAIL is included as a further dissolved component $\varphi ^{ID}$ of the interstitial fluid. As there is again no angiogenesis as in the proliferation model, the blood is treated likewise. Following this, the simplified set of constituents, solid $\varphi ^S$ with tumour material $\varphi ^{ST}$, blood $\varphi ^B$ and interstitial fluid $\varphi ^I$ with dissolved nutrients $\varphi ^{IN}$ and drugs $\varphi ^{ID}$, basically leads to the same reduced set of governing equations as for the proliferation model before. In their weak forms, these are overall momentum balance (105), the volume balance (106) of the interstitial fluid and the concentration balances (107) of nutrients and drugs, while the solid volume production is computed by the use of (108). This system is again solved for the primary variables, namely the solid displacement ${\mathbf{u}}_{S}$, the blood pressure $p^{BR}$, the interstitial fluid pressure $p^{IR}$ and the nutrient and drug concentrations, $c^{IN}_{m}$ and $c^{ID}_{m}$.

**Fig. 10:**
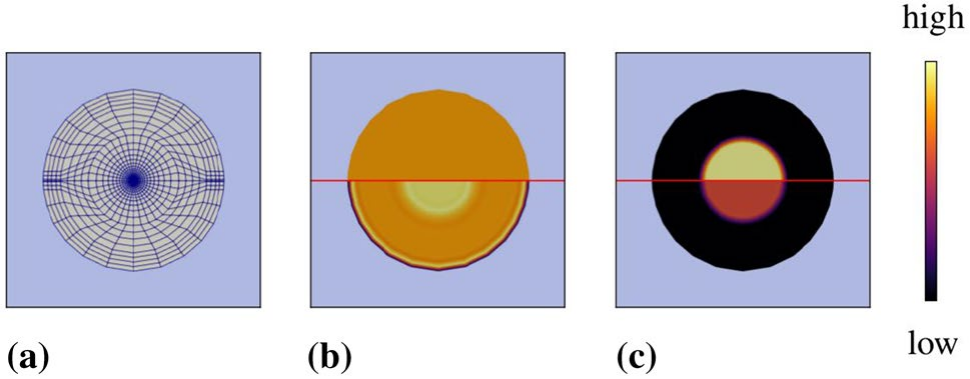
Numerical results according to the apoptosis experiments for a drug concentration of $10^{-8} \text {mol}/\text {m}^3$: **a** circular domain with diameter of $\mathrm 0.2\,\text{m}$; **b** initial and final drug concentration (top: initially, bottom: finally); **c** amount of viable cancer cells in [%] (top: initially 100%, bottom: finally 59.28%)

**Table 6:** Sensitivities of apoptosis parameters ranked from high to low values in accordance with the absolute mean $\mu ^{*}_i$ of their elementary effect

Parameter	$\mu ^{*}_i$
$\nu _{ID,\,{\mathrm{max}}}$	6.004
${{\tilde{\nu }}}_{ID,\,{\mathrm{max}}}$	0.648
$\breve{\nu }_{ID,\,{\mathrm{max}}}$	$1.79\times 10^{-3}$
$\lambda ^S_0$	$2.13\times 10^{-4}$
$\mu ^S_0$	$5.57\times 10^{-5}$
$K^{SI}$	$1.14\times 10^{-5}$
$\mu ^{IR}$	$1.10\times 10^{-5}$
$D^{IN}$	$1.72\times 10^{-7}$
$\mu ^{BR}$	$1.35\times 10^{-10}$
$K^{SB}$	$2.94\times 10^{-11}$

Parameters with vanishing $\mu ^{*}_i$ are omitted in the table

**Fig. 11:**
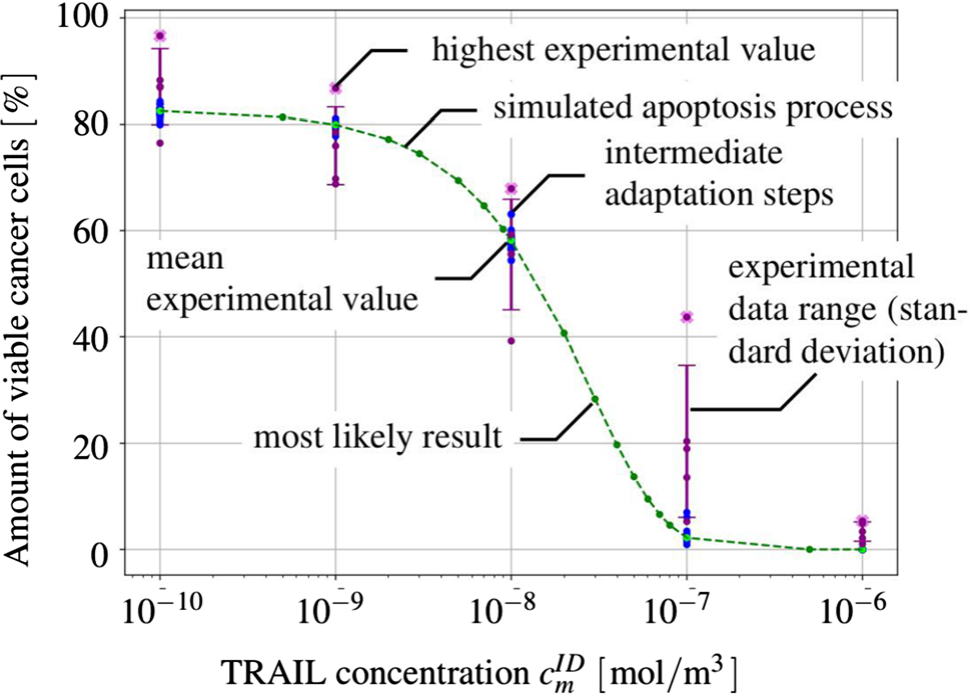
The mean viable cancer cell data from the experiment are depicted in purple, while the single viable cell ratios are depicted in dark red. The pink crosses indicate the experiment with the highest values. The simulation results from the optimisation are given in blue including the best result in light green. Additional simulation results corresponding to the best parameter set are given in dark green

*Parameter study and optimisation*: The goal of this apoptosis study is the parameter identification and optimisation of the most sensitive apoptosis input parameters. Here, the same FE mesh is used as in the proliferation study, cf. Fig. 8a or Fig. 10a, respectively. Again, the boundary conditions prescribe no solid displacements along the boundary line together with blood and initial fluid pressures of ${{\bar{p}}}^{BR}_0=1725$ ${\mathrm{Pa}}$ and ${{\bar{p}}}^{IR}_0=258\,{\mathrm{Pa}}$ and a nutrient concentration of $c^{IN}_m={\mathrm{1\,mol/m^3}}$. The same values are assumed initially distributed in the whole domain, while the drug concentration is similarly applied. Five different scenarios of drug concentrations, namely $c^{ID}_{m}\,\in \,[\,10^{-10},\,10^{-9},\,10^{-8},\,10^{-7},\,10^{-6}\,]$ given in mol/$\hbox {m}^3$, have been investigated at the same nutrient concentration, cf. Fig. 11. When the simulations are initialised, the metastasis is assumed in the centre of the experiment with a diameter of 0.04 m and an initial volume fraction of $n^{ST}_{0} = 0.1$, cf. the upper part of Fig. 10b. Over a period of 24 h, the drug triggers apoptosis within the cancer cells, thus leading to a cancer cell reduction, cf. Fig. 10c. During the same period, the drug concentrates in the cancerous domain, while the whole domain is shrinking, cf. the lower part of Fig. 10b.

As the proliferation model, the apoptosis model is governed by the same default and model parameters, cf. Table 3, with certain sensitivities, cf. Table 6. While all default parameters of Table 3 are taken from the literature, the apoptosis parameters of Table 7 have been optimised as the proliferation parameters before by the use of the elementary effect together with a minimisation of the likelihood function.

**Table 7:** Optimised apoptosis parameters

	Value	Unit
${\nu }_{\,ID,\,{\mathrm{max}}}$	$1.97\times 10^{-6}$	$[1/\text {s}]$
${\tilde{\nu }}_{\,ID,\,{\mathrm{max}}}$	$4.033\times 10^{2}$	$[\,\text {m}^{3}/(\text {mol}\,\text {s})\,]$
$\breve{\nu }_{\,ID,\,{\mathrm{max}}}$	$2.29\times 10^{-9}$	[ – ]

**Table 8:** Summary of proliferation and apoptosis parameters (ATP: adenosine triphosphate)

	Value	Unit	References
$\alpha ^B$	1863.08	$[\,{\mathrm{Pa}}\,]$	Chosen
$\alpha ^{{\mathrm{angio}}}$	$8.625\times 10^{6}$	$[({\mathrm{mol}}\cdot {\mathrm{J}}) /({\mathrm{kg}})^2]$	Chosen
$\mu ^A$	$3.5\times 10^{-3}$	$[{\mathrm{kg/J}}]$	Chosen
$\mu ^B$	$3.5\times 10^{-3}$	$[\,{\mathrm{1/Pa}}\,]$	Chosen
$p^{IR}_0$	258	$[\,{\mathrm{Pa}}\,]$	Boucher et al. (1997)
$p^{BR}_0$	1724	$[\,{\mathrm{Pa}}\,]$	Chosen
$c^{IN}_{0m}$	1	$[\,{\mathrm{mol/m}}^3\,]$	In the range of Abi-Saab et al. (2002)
$f_{\,{\mathrm{proli}}}$	$5.33\times 10^{-10}$	[ – ]	From ATP consumption during cell proliferation (Simpson et al. 2007)
${\bar{c}}^{IN}_m$	0.35	$[\,{\mathrm{mol/m^3}}\,]$	Optimised
${\tilde{c}}^{IN}_m$	0.31	$[\,{\mathrm{mol/m^3}}\,]$	Based on 0.90 ${\bar{c}}^{IN}_m$
$\breve{c}^{IN}_m$	0.26	$[\,{\mathrm{mol/m^3}}\,]$	Based on 0.75 ${\bar{c}}^{IN}_m$
${\tilde{c}}^{IC}_m$	$1.7\times 10^{-16}$	$[\,{\mathrm{mol/m^3}}\,]$	Chosen
${\bar{c}}^{ID}_m$	$10^{-11}$	$[\,{\mathrm{mol/m^3}}\,]$	Doubled sensitivity compared to Siegemund et al. (2012)
${\bar{c}}^{IV}_m$	$2.5\times 10^{-11}$	$[\,{\mathrm{mol/m^3}}\,]$	Chosen
$K_{gr}$	0.156	$[\,{\mathrm{mol/m^3}}\,]$	optimised
$\nu _{\oplus \,ST,\,{\mathrm{max}}}$	$4.15\times 10^6$	$[\,{\mathrm{1/s}}\,]$	Optimised
$\nu _{\ominus \,ST,\,{\mathrm{max}}}$	$10^{-5}$	$[\,{\mathrm{1/s}}\,]$	Chosen
$\nu _{ID,\,{\mathrm{max}}}$	$1.97\times 10^{-6}$	$[\,{\mathrm{1/s}}\,]$	Optimised
${{\tilde{\nu }}}_{ID,\,{\mathrm{max}}}$	$4.033\times 10^2$	$[\, {\mathrm{m}}^3/({\mathrm{mol}}\, {\mathrm{s}})\,]$	Optimised
$\breve{\nu }_{ID,\,{\mathrm{max}}}$	$2.29\times 10^{-9}$	$[\,-\,]$	Optimised
$\nu _{\ominus \,SB,\,{\mathrm{max}}}$	$10^{-6}$	$[\,{\mathrm{1/s}}\,]$	Chosen
$\nu _{IN,\,{\mathrm{basal}}}$	$10^{-16}$	$[\,{\mathrm{mol}}/({\mathrm{cells}}\, {\mathrm{s}})]$	Kallinowski et al. (1988)
$\nu _{\oplus \,IV,\,{\mathrm{max}}}$	0.155	$[\,{\mathrm{1/s}}\,]$	Based on Glucose transport (de Graaf et al. 2001) and calculated nutrient consumption from (96)$_2$

## Numerical studies

Proceeding from the material and model parameters given in Tables 3 and 8, the following chapter will give insight into the results of numerical experiments on 2-d and 3-d initial-boundary-value problems.

As not all parameters included in Table 8 could be found from the literature or on the basis of experimental data, some of them remained undetermined and had to be marked as chosen. However, those parameters that could be determined by optimisation generally fall into the category of parameters with high values of the absolute mean $\mu ^*_i$ of their elementary effect, cf. Table 6. At the same time, these parameters have a stronger impact on the numerical results compared to those with lower values of $\mu ^*_i$. For the latter group of parameters, their values had to be chosen, unless they could not be found elsewhere. Although these parameters only have a low impact on the numerical results, this does not mean that they have been chosen arbitrarily. Instead, they have been chosen with respect to sound numerical results. For example, the parameters $\alpha ^B$, $\alpha ^{{\mathrm{angio}}}$, $\mu ^A$, $\mu ^B$ and ${\bar{c}}^{IV}_{m}$ together with $\psi ^{B,\,{\mathrm{angio}}}$ after Eq (70) govern the relation of the sigmoid function (67). These values are chosen for a reasonable steepness of the functions $s^B(p^{{\mathrm{dif}}})$, $p^{\mathrm{dif}}(s^B)$ and $\rho ^{BR}\psi ^B(s^B)$.

In addition to the above and in contrast to the values given in Table 3, the Lamé constants have been changed towards the values of Table 9. While the parameters of Table 3 represent white matter parameters (Budday et al. 2015) that correspond to the experimental set-up, the values of Table 9 are based on an optimisation of the whole brain, white and gray matter, cf. Soza et al. (2004), and additionally emphasise values that have been found by experiments on pig brains.

The following studies proceed again from the full set of governing equations (105)–(108) that are further solved by the use of the FE solver PANDAS. This procedure guarantees a fully coupled numerical analysis of all features including growth and atrophy of cancer-cell clusters as well as solid deformation and stress, flow of blood and the interstitial-fluid mixture together with diffusion of fluid species.

### Two-dimensional study of the overall model

Based on the above statements, a 2-d computation of growth and atrophy of a single lung cancer metastasis within the brain tissue is simulated. The goal of the following study is to present the capability of the introduced model including cancer cell proliferation, angiogenesis, necrosis and apoptosis.

While the numerical examples of the preceding chapter, that have been needed to identify material parameters, exhibit boundary-value problems with rotational symmetry, the actual problem is fully two-dimensional.

**Fig. 12:**
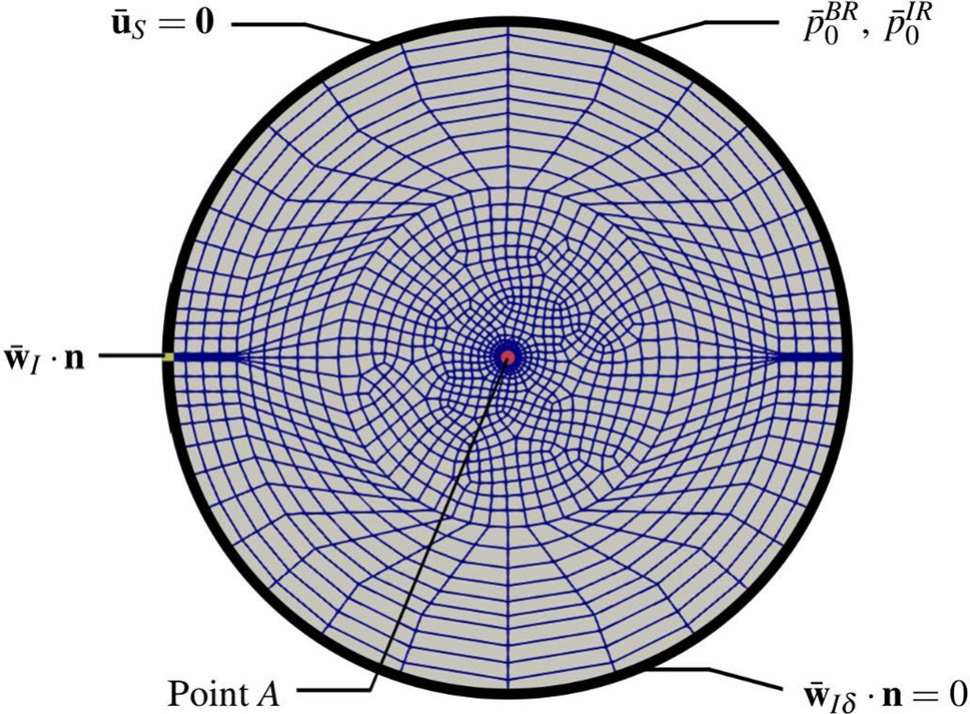
Geometry and mesh (diameter 0.2 m) of the 2-d initial-boundary-value problem. The outer boundary (black rim) is spatially fixed at $\bar{{\mathbf {u}}}_S = {\mathbf{0}}$, while the blood and initial-fluid pressures are prescribed to ${\bar{p}}^{BR}_0 = 1725$ Pa and ${\bar{p}}^{IR}_0 = 258$ Pa both at the boundaries and as initial conditions throughout the domain. The flow of the interstitial fluid species across the boundary is set to $\bar{{\mathbf {w}}}_{I\delta }\cdot {\mathbf{n}} = 0$, while the solvent flow is not restricted. At the infusion site (yellow mark), the inflow condition $\bar{{\mathbf {w}}}_{I}\cdot \mathbf{n}$ of the interstitial fluid including its species varies over time. The red point (Point *A*) is in the centre of the domain, from where the metastasis grows

**Fig. 13:**
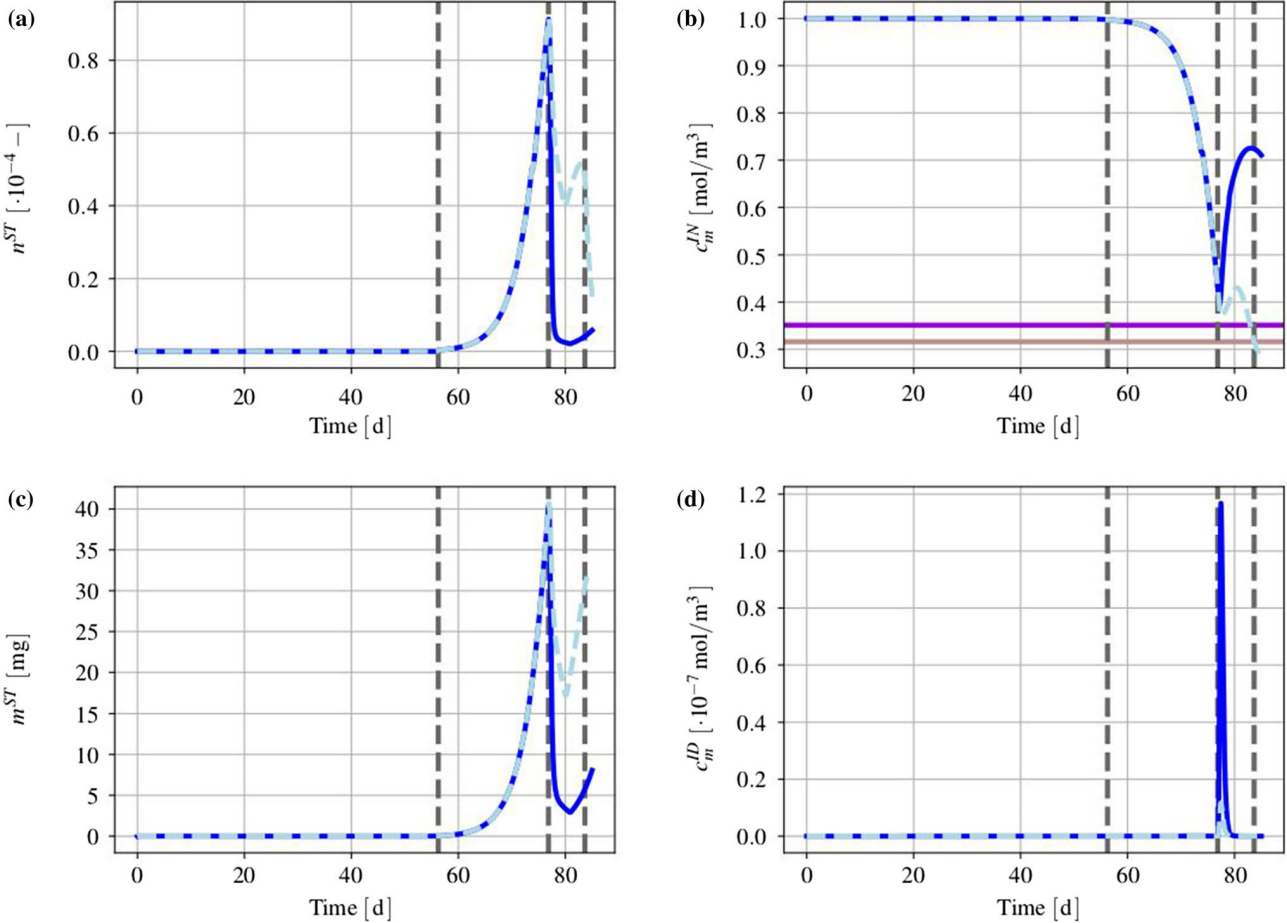
Proliferation and apoptosis of the lung cancer metastasis in the brain at an early evolution state, all values at Point *A*. **a** Cancer volume fraction $n^{ST}$ versus time, **b** nutrient concentration $c^{IN}_m$ versus time, **c** aggregated nutrient mass $m^{ST}$ versus time and **d** medical drug (TRAIL) concentration versus time. The dark blue lines correspond to the infusion with the high drug concentration of $1.5\times 10^{-3}\,\text {mol}/\text {m}^3$, while the light blue lines exhibit the infusion with the low drug concentration of $1.5\times 10^{-4}\,\text {mol}/\text {m}^3$. At point *A*, these infusions result in concentrations of $1.16761\times 10^{-7}\,\text {mol}/\text {m}^3$ or of $1.16773\times 10^{-8}\,\text {mol}/\text {m}^3$, respectively

#### Set-up

The model includes all introduced primary variables, namely the solid displacement ${\mathbf{u}}_S$, the blood and interstitial fluid pressures, $p^{BR}$ and $p^{IR}$, as well as the nutrient, cancer cell, VEGF and drug concentrations, $c^{IN}_m$, $c^{IC}_m$, $c^{IV}_m$ and $c^{ID}_m$. As before, the study is carried out on a circular domain with a radius of 0.2 m representing a geometrically simplified model of a brain bathing in a rigid skull with an initial cancer cell cluster in the middle, cf. Fig. 12. Concerning the initial and boundary conditions, the solid skeleton is fixed along the boundary with $\bar{\mathbf{u}}_S\equiv {\mathbf{0}}$. Constant fluid pressures ${\bar{p}}^{BR}_0 = 1725$ Pa and ${\bar{p}}^{IR}_0 = 258$ Pa are prescribed at the boundary and initially in the entire domain together with $n^B_0=0.05$ and $n^I_0=0.20$. Moreover, all fluid fluxes across the boundary are set to zero with the exception of the interstitial fluid-solvent flux $\bar{\mathbf{w}}_{IL}\cdot {\mathbf{n}}$. In addition, the injected interstitial fluid flux $\bar{\mathbf{w}}_{I}\cdot {\mathbf{n}}$ including the drug-species at the yellow point in the middle of the left side, cf. Fig. 12, can vary over time, such that a therapeutic infusion can be applied.

**Fig. 14:**
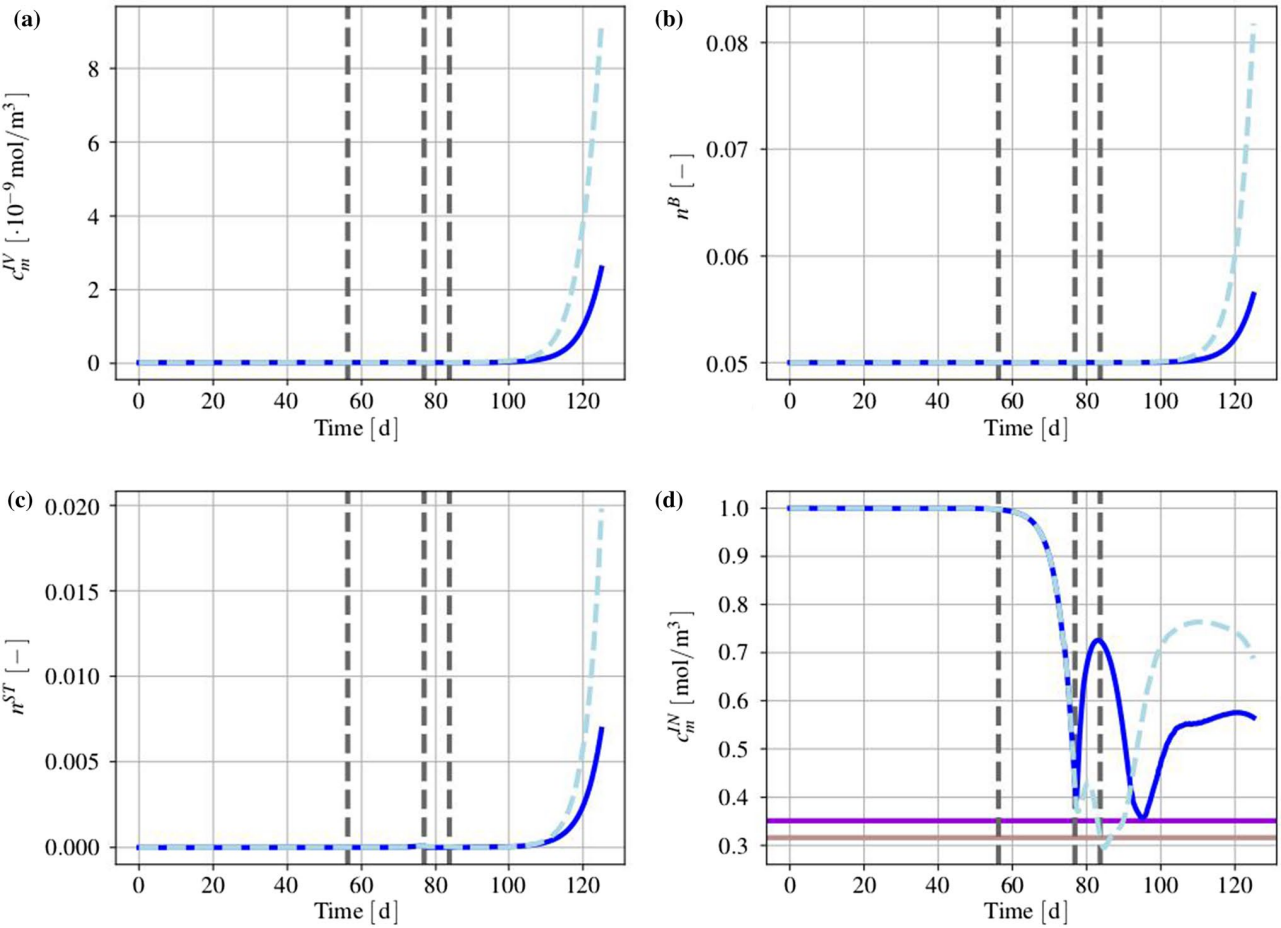
After an early therapeutic treatment, cf. Fig. 13, the cancer cells proliferate gain and start to send out vascular endothelial growth factors (VEGF) in order to cope with the decreasing nutrient concentration and to sustain their basal reactions as well as their cell divisions, all values at Point *A*. **a** Progression of the VEGF concentration $c^{IV}_m$ versus time, **b** blood volume fraction $n^B$ versus time, **c** tumour volume fraction $n^{ST}$ versus time and **d** nutrient concentration versus time. The dark blue lines correspond to the infusion with the high drug concentration of $1.5\times 10^{-3}\,\text {mol}/\text {m}^3$, while the light blue lines exhibit the infusion with the low drug concentration of $1.5\times 10^{-4}\,\text {mol}/\text {m}^3$. The dark purple and the umber line indicate the minimum nutrient concentration ${\bar{c}}^{IN}_m$ needed to initiate proliferation, and the threshold concentration $\breve{c}^{IN}_m$, where necrosis occurs at concentrations below

**Fig. 15:**
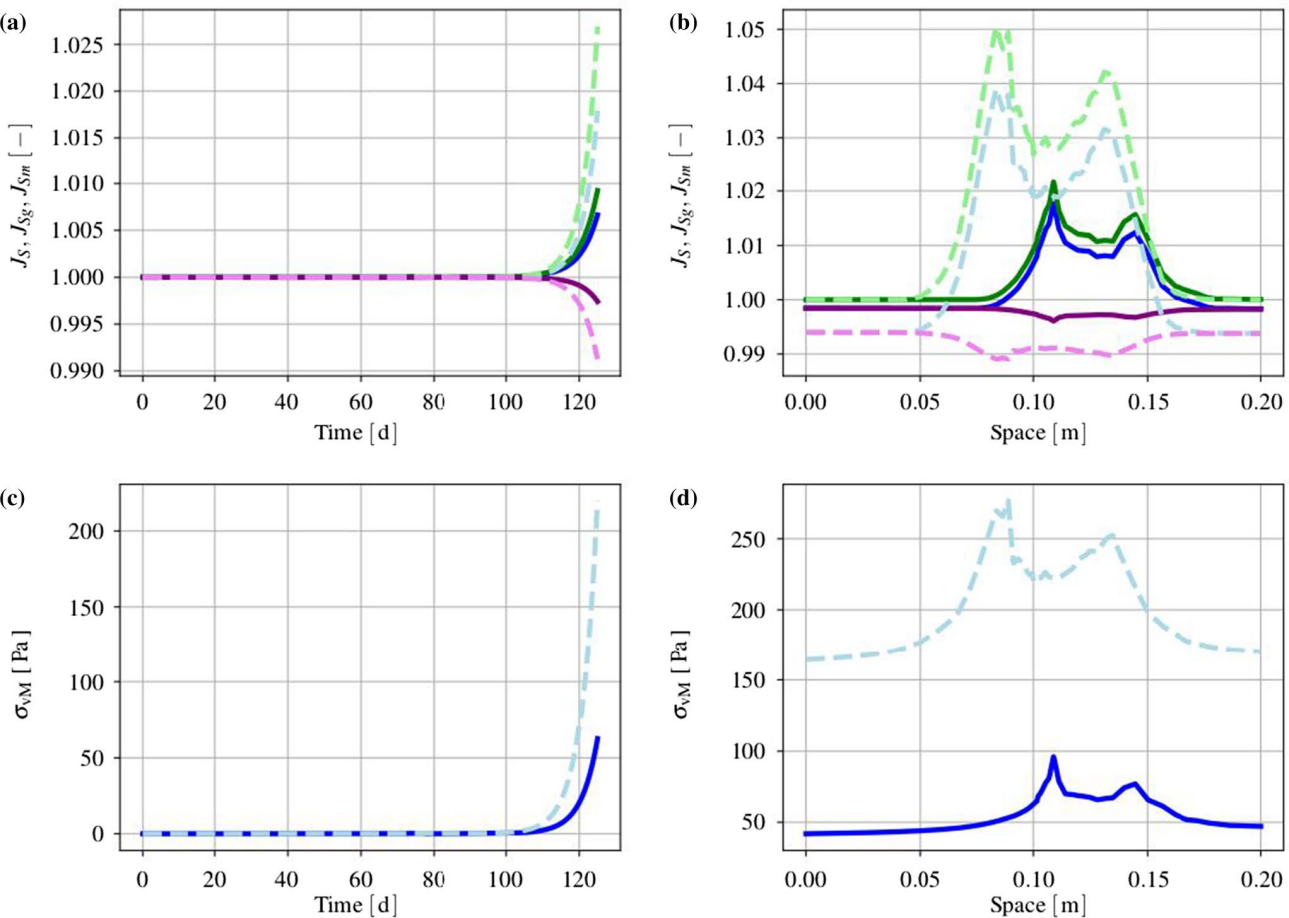
Solid deformations and stresses versus time and space. **a**, **b** Jacobian determinants $J_S$ of the total deformation, $J_{Sg}$ of the deformation induced by growth (here apoptosis), and $J_{Sm}$ of the purely mechanical deformation, cf. (73) and (74). Solid lines correspond to the higher medication of $1.5\times 10^{-3}\,\text {mol}/\text {m}^3$, dashed lines to the lower medication of $1.5\times 10^{-4}\,\text {mol}/\text {m}^3$ with $J_S$ in blue, $J_{Sg}$ in green and $J_{Sm}$ in purple. **c**, **d** von Mises stresses. **b**, **d** display Jacobians and von Mises stresses at day 125 along a horizontal line through Point *A* at 0.10 m

**Fig. 16:**
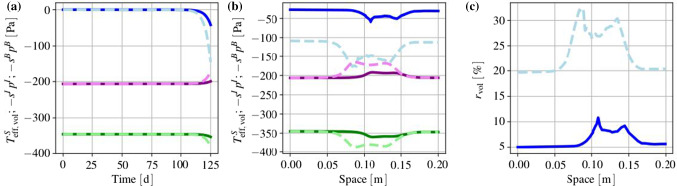
**a**, **b** Effective volumetric solid stress $T^S_{{\mathrm{eff,\,vol}}}$ (blue) together with partial blood (violet) and interstitial-fluid (green) pressures $s^Bp^{BR}$ and $s^Ip^{IR}$. Dark solid lines correspond to the high drug concentration of $1.5\times 10^{-3}\,{\mathrm{mol/m}}^3$and light dashed lines to the low drug concentration of $1.5\times 10^{-4}\,{\mathrm{mol/m}}^3$. **a** Results at point *A* over time between day zero and day 125, **b** results over space, **c** ratio of $T^S_{{\mathrm{eff,\,vol}}}$ over the initial negative pore pressure $p^{FR}_0=s^B_0p^{BR}_0+s^I_0p^{IR}_0$ with peak values of 10.80% (high medication) and 32.08% (low medication). **b**, **c** Values along the horizontal line through Point *A* at day 125

The infusion is assigned to last for 8 h including a 2-h period of linear infusion rate increase and another 2-h period of linear infusion rate decrease. Altogether, two infusion scenarios are investigated. In the first case, the maximum medication rate reaches $1.5\times 10^{-10}\,{\mathrm{mol/(m^2\, s)}}$ at the infusion site resulting from a therapeutic agent concentration of $1.5\times 10^{-3}\,{\mathrm{mol/m^3}}$ at an interstitial fluid velocity of $10^{-7}\,{\mathrm{m/s}}$. In a second case, the maximum medication rate is reduced to $1.5\times 10^{-11}\,{\mathrm{mol/(m^2\, s)}}$ resulting from $1.5\times 10^{-4}$ ${\mathrm{mol/m^3}}$ at the same interstitial fluid velocity of $10^{-7}\,{\mathrm{m/s}}$. As the medical drug is injected with the interstitial fluid, the dissemination of the drug is governed not only by diffusion but also by the fluid flow, thus resulting in a convection-enhanced delivery (CED) of the medical drugs. Furthermore, note in passing that in contrast to previous studies, such as Linninger et al. (2008) or Ehlers and Wagner (2015), the infusion site is in this example not located at the end of an infusion needle intruded into the brain but at the outer boundary. As a result, the motion of the interstitial fluid with included medical drug is governed by the fluid’s influx velocity of $10^{-7}\,\text {m}/\text {s}$. In case of an approximately vanishing solid velocity, the influx velocity equals the seepage velocity, such that the corresponding filter velocity results in $2\times 10^{-8}\,\text {m}/\text {s}$ at an interstitial fluid volume fraction of $n^I=0.2$.

**Table 9:** Modified elasticity parameters used in Sect. 6

	Value	Unit	References
$\mu ^{S}_{0}$	$2.27\times 10^{3}$	$[{\mathrm{Pa}}]$	Related to Soza et al. (2004)
$\lambda ^{S}_{0}$	$2.42 \times 10^{4}$	$[{\mathrm{Pa}}]$	Related to Soza et al. (2004)

#### Results

For the initial-boundary-value problem described above, Fig. 13 exhibits the process of proliferation and related nutrient consumption as well as the response of a growing tumour to an early treatment. The latter, of course, is only possible if the tumour has been discovered at this early state before angiogenesis has started. The results presented in Figs. 13, 14, 15 and 16 display values at the centre of the investigated domain (Point *A*). In the course of the treatment study, two different medication examples have been considered displayed by a solid dark blue and a dashed light blue line. While the dark blue line corresponds to a high drug concentration during the medical treatment, the light blue line belongs to the lower drug concentration. Furthermore, Figs. 13 and 14 contain three dashed grey vertical lines. The left of them indicates the micrometastatic switch approximately at day 58. The middle line at approximately day 77 represents the moment, when the tumour can firstly be detected and the medication can start, while the right line at approximately day 84 characterises the moment of VEGF release. In addition to the vertical lines, Figs. 13b and 14d furthermore exhibit two horizontal lines. While the upper purple line indicates the minimum nutrient concentration ${{\bar{c}}}^{IN}_m$ necessary for the tumour to regrow, the lower umber line characterises the nutrient threshold ${{\tilde{c}}}^{IN}_m$, where a nutrient concentration below that line would necrotise the tumour.

*Proliferation:* Once a metastasis-based tumour cell cluster has settled in the brain, a volume increase of the tumour takes place under fully nutrient-supplied conditions. This can be seen from Fig. 13, where the tumour volume fraction $n^{ST}$, the tumour mass $m^{ST}$, the nutrient concentration $c^{IN}_m$ and the drug concentration $c^{ID}_m$ are plotted versus time. It is furthermore seen from Fig. 13a–c that the tumour increases exponentially beyond the micrometastatic switch at day 58, while the nutrient concentration strongly decreases as a result of nutrient consumption of the growing tumour. This behaviour stops at day 77, when the tumour has been detected and a medical treatment has been initiated, cf. Fig. 13d.

*Apoptosis:* Once the medication has started, the dark and light blue lines follow different paths describing the situation at Point *A* of the tumour site. While the dark blue line corresponds to a maximum drug infusion rate of $1.5\times 10^{-10}$ ${{\mathrm{mol/(m^{2}}} \, \mathrm{s})}$, the light blue line proceeds from a maximum value of only $1.5\times 10^{-11}$ ${{\mathrm{mol/(m^{2}}} \, \mathrm{s})}$. Following this, it is seen from Fig. 13a that, depending on the medication rate, the dark blue line exhibits a much steeper descent of the tumour volume fraction than the light blue one. Furthermore, when the medication stops at approximately day 80, the tumour recreates much faster when the lower medication has been applied instead of the higher one. These results also correspond to the nutrient concentration at the tumour site that decreases when the tumour grows and recreates when the tumour growth ends, however, following different paths depending on the chosen drug infusion rate.

Corresponding to the course of the tumour volume fraction $n^{ST}$, one observes the growth of the aggregated tumour mass $m^{ST}$, cf. Fig. 13c, that reaches 40 mg when the medication starts. It is furthermore seen from Fig. 13d, how different the two medications represented by the dark and light blue lines are. While the dark blue line describes a drug concentration of the infusion of $1.5\times 10^{-3}\,{\mathrm{mol/m^3}}$, the light blue line only results from a concentration of only $1.5\times 10^{-4}\,{\mathrm{mol/m^3}}$. As a result of the CED process, the drug concentration at Point *A* at the tumour site, cf. Fig. 13d, reaches approximately $1.2\times 10^{-7}$ ${\mathrm{mol/m^3}}$, in the first case, and approximately $1.2\times 10^{-8}\,{\mathrm{mol/m^3}}$, in the second case.

*Angiogenesis and necrosis:* When the medication has stopped at day 80, the tumour starts regrowing, cf. Fig. 13a. Note that this regrowth cannot be observed in Fig. 14c as a result of the scale difference between these two figures. However, it is seen from Fig. 13a that, depending on the medication rate, the dark blue line exhibits a much steeper descent of the tumour volume fraction than the light blue one. Furthermore, after the medication, the tumour recreates much faster when the lower medication has been applied instead of the higher one. These results also correspond to the nutrient concentration at the tumour site that decreases when the tumour grows and recreates when the tumour growth ends, however, following different paths depending on the chosen drug-infusion rate. On the other hand, the nutrient consumption belonging to the dark blue line is so high that the dark blue line touches the purple line, while the light blue line enters the critical region at the umber line. However, the tumour succeeds in both cases to continue growing.

Stimulated by the heavy nutrient consumption, VEGF is released at day 84 and blood vessels start to grow towards the tumour, which also results in an increase of the nutrient concentration, cf. Fig. 14b, d. After day 120, the nutrient concentration decreases again and the tumour would further on undergo necrosis unless VEGF is released again.

Figure 15 exhibits the volumetric solid deformations expressed by the Jacobian determinants $J_S$ of the total deformation, $J_{Sg}$ of the deformation induced by growth (including apoptosis), and $J_{Sm}$ of the purely mechanical deformation, cf. (73) and (74). Solid lines correspond to the higher medication of $1.5\times 10^{-3}\,\text {mol}/\text {m}^3$and dashed lines to the lower medication of $1.5\times 10^{-4}\,\text {mol}/\text {m}^3$ with $J_S$ in blue, $J_{Sg}$ in green and $J_{Sm}$ in purple. As the Jacobians stay at 1.00 in the undeformed state up to approximately day 100, it is concluded that measurable deformations only start after the angiogenesis. Figure 15a displays Jacobians with values either greater or less than one resulting in volumetric extensions or compressions. As the stresses are obtained from $J_{Sm}=J_S/J_{Sg}$ in purple, the growing tumour leads to compressive stresses after angiogenesis, cf. Fig. 15a. Figure 15b that, as well as Fig. 15d represents its values at day 125 along a horizontal line through Point *A*, moreover exhibits that the highest values of the Jacobian determinants are not at the centre of the domain at 0.1 m but shifted to the left and right. On the other hand, the centre of the metastasis between the two peaks exhibits necrosis leading to a reduction of the volume fraction, whereas the boundary could be supplied with nutrients and proliferates as a result of the angiogenesis process.

Figure 15c shows the von-Mises stresses with higher values for the lower and lower value for the higher medication. Furthermore, Fig. 15d presents the same shifting behaviour for the von Mises stresses as for the Jacobians with the highest value of approximately 277 Pa. Please note that small numerical instabilities occurred that can be seen in these figures due to the sharp switch based on the Heaviside function included in the apoptosis model (94) and (99) at the threshold concentrations ${\bar{c}}^{ID}_m$ and ${\bar{c}}^{IN}_m$.

As the von-Mises stress $\sigma _{vM}=\sqrt{\frac{3}{2}\mathbf{T}^{SD}_{{\mathrm{eff}}}\cdot {\mathbf{T}}^{SD}_{{\mathrm{eff}}}}$, cf. Fig. 15d, is a positive mean of the deviator of the total solid effective stress ${\mathbf{T}}^S_{{\mathrm{eff}}}$, it is worth to take a look at the volumetric stresses

113 $$\begin{aligned}&\textstyle T^S_{{\mathrm{eff, \,vol}}}=\frac{1}{3}({\mathbf{T}}^S_{\mathrm{eff}}\cdot \,{\mathbf{I}}\,)\quad \text{ and }\nonumber \\&\textstyle T_{{\mathrm{vol}}}=-p^{FR}+\frac{1}{3}({\mathbf{T}}^S_{\mathrm{eff}}\cdot \,{\mathbf{I}}\,)\,, \end{aligned}$$

cf. (40)–(42). At this point, it should be noted that in case of a fully 2-d problem, as it is solved here, $T^S_{{\mathrm{eff,\,vol}}}$ reduces to $\frac{1}{2}[\,(T^S_{\mathrm{eff}})_{11}+(T^S_{{\mathrm{eff}}})_{22}\,]$. Like the graphs of the Jacobians and the von-Mises stress, cf. Fig. 15, it is seen from Fig. 16 that the effective volumetric stress $T^S_{{\mathrm{eff, \,vol}}}$ as well as the partial pore pressures $s^Bp^{BR}$ and $s^Ip^{IR}$ only reacts to the tumour growth after angiogenesis initialised around day 83. With increasing growth, the brain tissue is pressed aside and gets under pressure. Once this has happened, it is seen from Fig. 16a that the reaction to tumour growth at Point *A* strongly depends on the medication with the stronger reaction depending on the lower drug medication (dashed lines). Figure 16b displays the volumetric stress and the pressures along a horizontal line through Point *A*. Here, it is seen as a result of the injection site at the left of the domain that at day 125 the tumour centre has been shifted to the right with an amount depending on the medication. Note that necrosis occurs in both cases, but is not as dominant as apoptosis due to the medication. However, as the tumour regrows after the medication has stopped, the effect on stress and pressures is larger in case of the lower medication. In addition to the individual graphs included in Fig. 16a, b, it is worth to compare $T^S_{{\mathrm{eff, \,vol}}}$ to the effective pore pressure $p^{FR}$, thus comparing the two parts of $T_{{\mathrm{vol }}}$ to each other. This can be done by taking the ratio of $T^S_{{\mathrm{eff, \,vol}}}$ over the negative effective pore pressure yielding

114 $$\begin{aligned} r_{{\mathrm{vol}}}=\frac{T^S_{\mathrm{eff, \,vol}}}{-p^{FR}}\,~~\text{ such } \text{ that }~~ T_{{\mathrm{vol}}}=-(1+r_{\mathrm{vol}})p^{FR}\,. \end{aligned}$$

As $p^{FR}=s^Bp^{BR}+s^Ip^{IR}$ is nearly constant, $p^{FR}$ in (114) has been substituted by its initial value $p^{FR}_0$ with the result that

115 $$\begin{aligned} r_{{\mathrm{vol}}}=\frac{T^S_{\mathrm{eff, \,vol}}}{-p^{FR}_0}\,. \end{aligned}$$

Figure 16c exhibits the course of $r_{\mathrm{vol}}$ at day 125 along a horizontal line through Point *A* with peak values of 10.80% (high medication) and 32.08% (low medication). Finally, it is again seen that the tumour centre shifts to the right depending on drug concentration of the infusion.

**Fig. 17:**
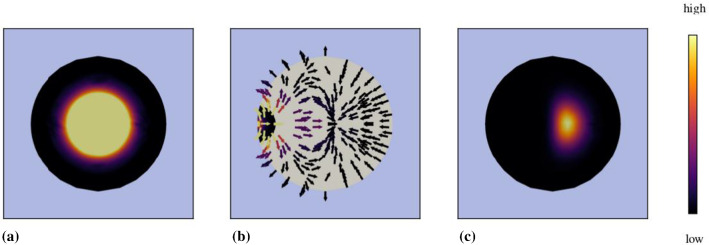
Results of the infusion of the therapeutic agent. **a** Volume fraction of the metastasis before the infusion has started with $n^{ST}\,\in \,[\,0,\,\,8.7\times 10^{-6}\,]$, **b** therapeutic agent concentration $c^{ID}_m\,\in \,[\,10^{-5},\,\,10^{-7}\,]$ in ${\mathrm{mol/m}}^3$ with arrows of the seepage velocity of the interstitial fluid solvent $\left| {\mathbf {w}}_{IL}\right| \,\in \,[\,10^{-12},\,10^{-11}]$ in ${\mathrm{m/s}}$ with colour-coded magnitude at the initiation of the infusion process, **c** metastasis volume fraction $n^{ST}\,\in \,[\,0,\,9.2\cdot \,10^{-6}\,]$ after the therapeutic agent concentration has declined below the threshold level of ${\bar{c}}^{ID}_m = 10^{-11}\,{\mathrm{mol/m}}^3$

To close up, Fig. 17a shows the metastasis volume fraction before the medical treatment has been started with the tumour site in the centre of the domain, while Fig. 17c exhibits the same tumour, however, once the treatment is over and the drug concentration $c^{ID}_m$ has decreased below the threshold level of ${\bar{c}}^{ID}_m = 10^{-11}$ $\mathrm {mol/m^3}$. As before and as result of the infusion from the left side, it is seen that the centre of the reduced metastasis is now more to the right than before. Completing the 2-d example, Fig. 17b displays a state in between the situations at (a) and (c), where the therapeutic agent concentration is given together with the arrows of the seepage velocity ${\mathbf{w}}_{IL}$ of the interstitial fluid solvent with colour-coded normalised values. Note again that, as a result of the little masses of the fluid solutes compared to the solvent mass, ${\mathbf{w}}_{IL}\approx {\mathbf{w}}_{I}$.

### Three-dimensional simulation of a tumour treatment

#### Set-up

**Fig. 18:**
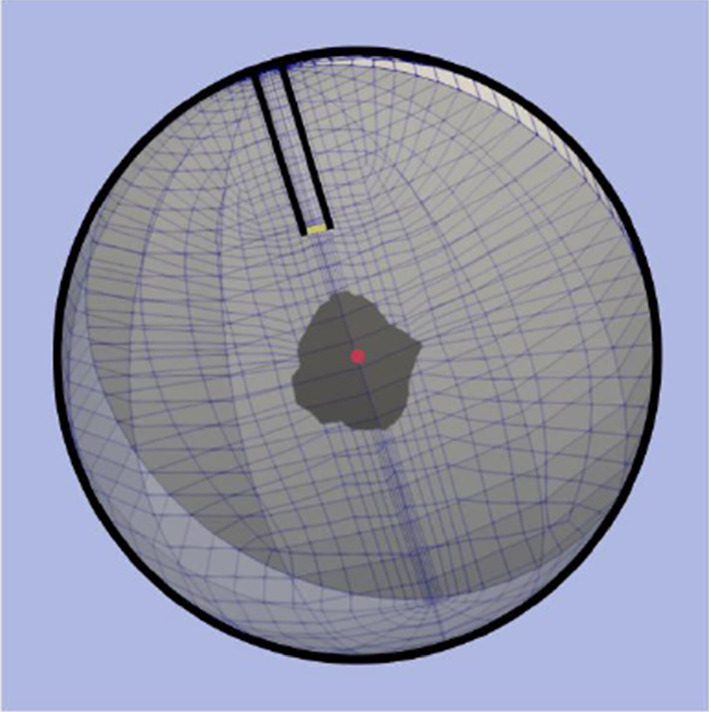
Spherical domain and mesh of the 3-d initial-boundary-value problem (diameter 0.2 m) with boundary conditions as in the 2-d problem before, tumour site seen as black area with a red mark (Point *A*) in the centre, and medical treatment through the infusion needle (inner radius 3.6 mm, insertion length 50 mm) close to the top

The present example, cf. Fig. 18, is based on a 3-d domain with a diameter of 0.2 m and a red mark in the centre. In a realistic scenario, a brain tumour metastasis is usually detected by sufficiently resolved medical imaging techniques, once it has reached a certain size. In the present example, the tumour is assumed to have been found as the irregular white region around the centre of the domain, such that a surgical intervention can be planned. The initial location and shape of the cancer cells or of the metastatic volume fraction, respectively, are based on a MRT scan of a meningioma which is used here in the sense of a proxy with data originating from the 3DSlicer Registration Case Library found on the MIDAS webpage (https://www.insight-journal.org/midas) in the section National Alliance for Medical Image Computing (NAMIC). Apart of the spherical domain consisting of 5104 spatial Taylor–Hood elements, the problem is completely non-symmetric, meaning that a fully 3-d problem had to be investigated. The initial and boundary conditions are chosen as the same that have been applied in the 2-d problem before. The planned surgery has been defined as a CED-based medical drug infusion through a little whole in the skull, such that an infusion needle with inner radius of 3.6 mm could be inserted at a length of 50 mm. Different infusion scenarios, cf. Fig. 20, have been studied in order to find the most appropriate surgery result.

**Fig. 19:**
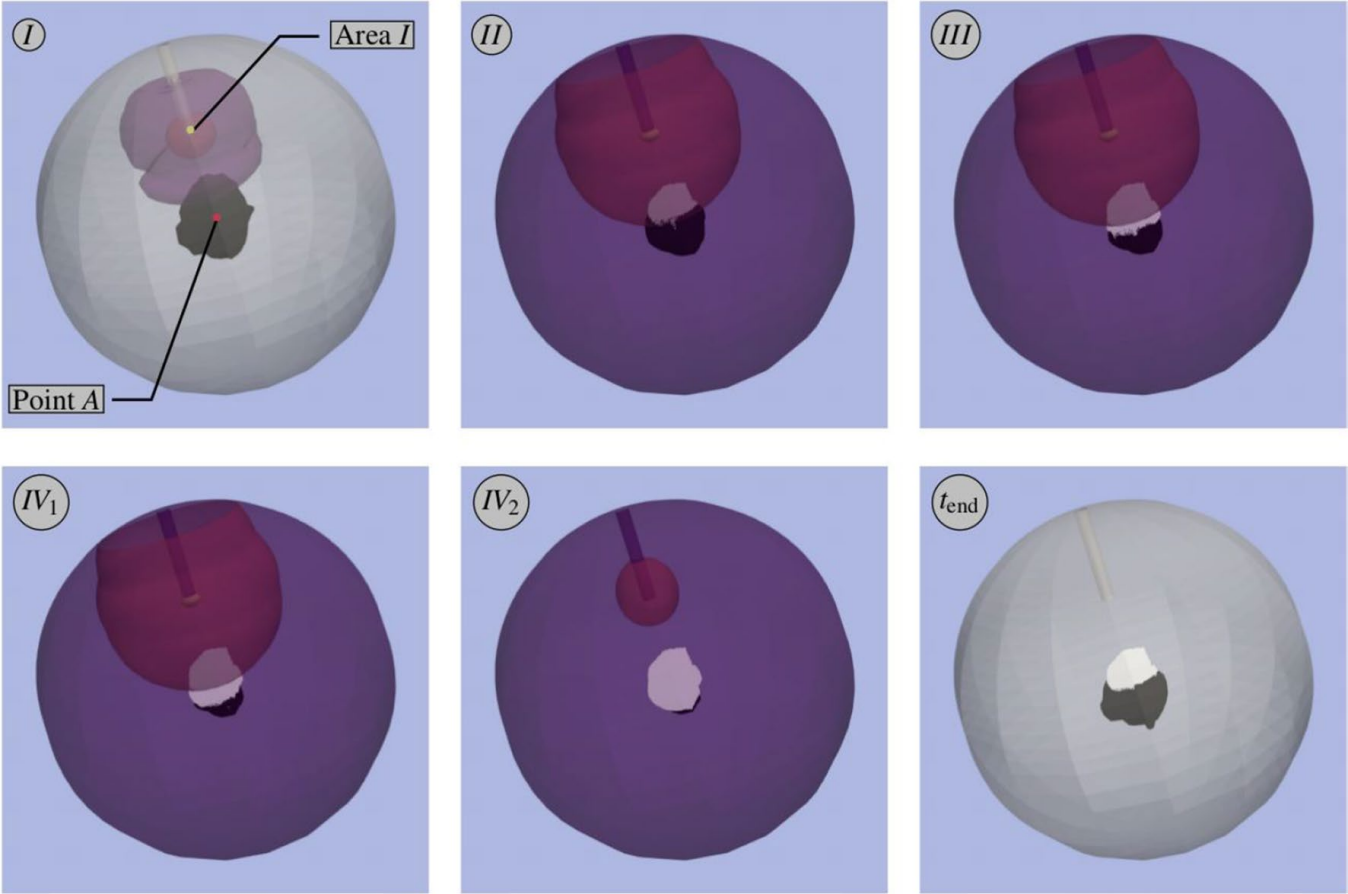
Results of the 3-d computations corresponding to four infusions with a drug concentration of $c^{ID}_m = 2.5\times 10^{-4}\,\text {mol}/\text {m}^3$ at an interval of five days. The infusions are inserted at the end of the infusion shaft (Area *I*). The metastasis volume fraction is illustrated as contour volume corresponding to $n^{ST} = 0.01$ with an initial metastasis location in black. During the phases *I* to *IV*, coloured isosurfaces enclose areas with different minimum concentrations (purple: $c^{ID}_m\ge {{\bar{c}}}^{ID}_m=10^{-11}\,\text {mol}/\text {m}^3$ | red: $c^{ID}_m\ge \,5\times 10^{-8}\,\text {mol}/\text {m}^3$ | yellow: $c^{ID}_m\ge \,10^{-5}\,\text {mol}/\text {m}^3$)

**Fig. 20:**
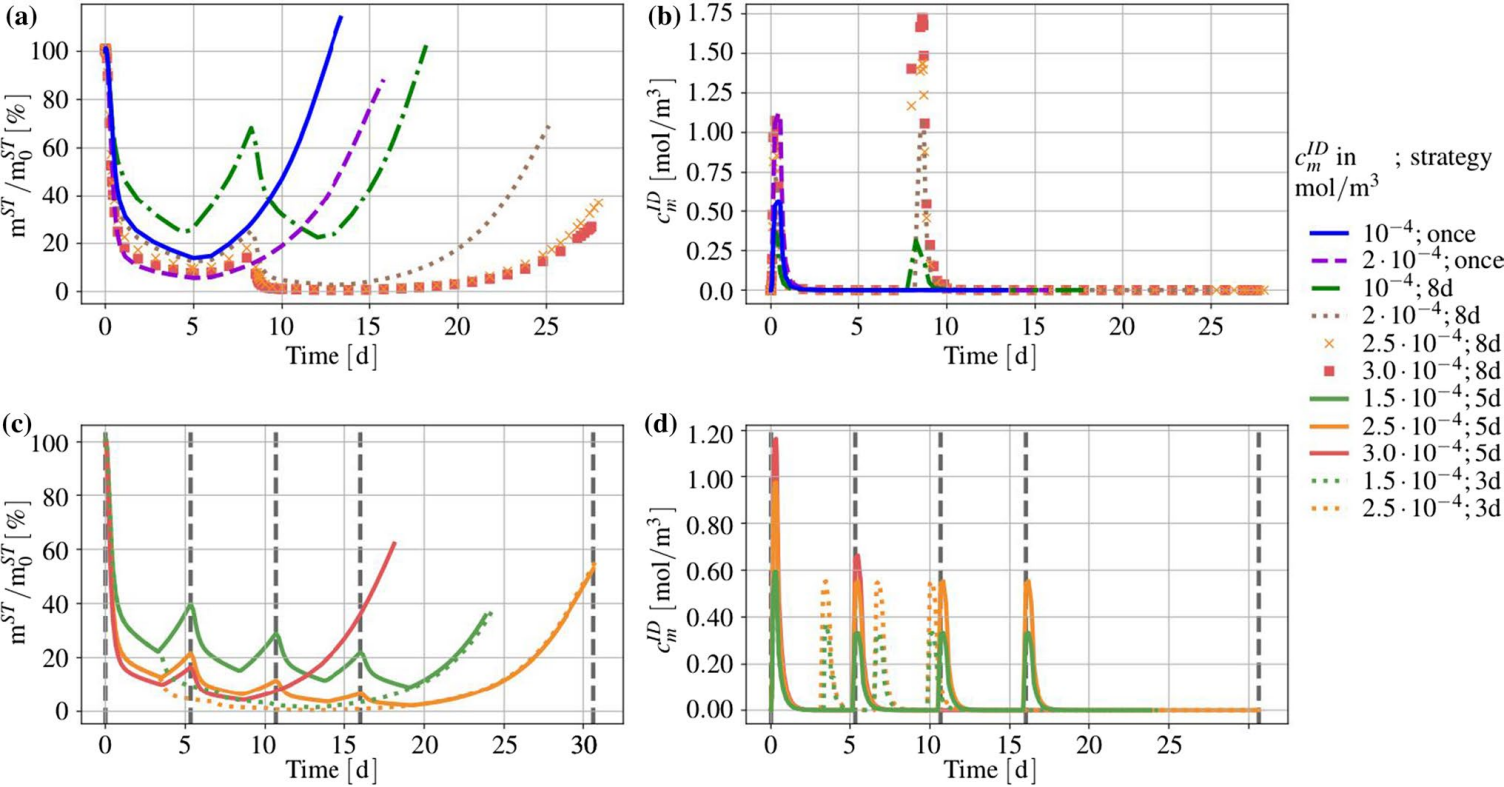
Results of 3-d simulations by the use of different infusion strategies. **a** Metastasis mass $m^{ST}$ over its initial mass $m^{ST}_0$ and **b** drug concentrations $c^{ID}_m$ at the centre of the domain (Point *A*) both versus time for a single and a double application (application interval 8 days) with TRAIL at the following values of $c^{ID}_m$: $10^{-4}\,{\text{mol/m}}^3$ (once, solid blue line), $2\times 10^{-4}\,{\mathrm{mol/m}}^3$ (once, dashed purple line), $10^{-4}\,{\mathrm{mol/m}}^3$ (twice, dash-dotted green line), $2\times 10^{-4}\,{\mathrm{mol/m}}^3$ (twice, dotted brown line), $2.5\times 10^{-4}\,{\mathrm{mol/m}}^3$ (twice, crossed orange line), $3\times 10^{-4}\,{\mathrm{mol/m}}^3$ (twice, dotted red line). **c** Metastasis mass $m^{ST}$ over its initial mass $m^{ST}_0$ and **d** drug concentrations $c^{ID}_m$ at the centre of the domain (Point *A*) both versus time for different application strategies with TRAIL concentrations of $1.5\times 10^{-4}\,{\mathrm{mol/m}}^3$ (solid green line, 4 applications), $2.5\times 10^{-4}\,{\mathrm{mol/m}}^3$ (solid orange line, 4 applications), $3\times 10^{-4}\,{\mathrm{mol/m}}^3$ (solid red line, 2 applications) all at an application interval of 5 days, while the concentrations $\mathrm {1.5\times 10^{-4}\,mol/m^3}$ (dotted green line, 2 applications), $\mathrm {2.5\times 10^{-4}\,mol/m^3}$ (dotted orange line, 2 applications) correspond to an application interval of 3 days

#### Results

During neurosurgical drug injection of the metastasis, cf. Fig. 19, four infusions (Phases $I-IV$) with a drug concentration of $c^{ID}_m=\mathrm{2.5\times 10^{-4}}$ ${\mathrm{mol/m^3}}$ are given at an interval of five days. Numerically, Phase *I* displays the treatment 25 s after the computation has been initiated with the tumour region around Point *A* in black. Obviously, the highest drug concentration is here exactly at the outlet of the infusion needle (Area *I*) surrounded by red and purple areas with threshold concentrations of $\mathrm{5\times 10^{-8}}$ ${\mathrm{mol/m^3}}$ and ${\mathrm{10^{-11}}}$ $\mathrm{mol/m^3}$. Once the therapeutic drug reaches the tumour site, the degradation of tumour material starts displayed by a colour change of the tumour volume fraction from black to white. After the second treatment five days after the first infusion (Phase *II*), the whole domain is at least in purple with a drug concentration of more than ${\mathrm{10^{-11}}}$ ${\mathrm{mol/}}$ ${\mathrm{m^3}}$, while the red area with $c^{ID}_m\ge {\mathrm{5\times 10^{-8}}}$ ${\mathrm{mol/m^3}}$ has reached the tumour. As a result, the degraded part of the tumour is rapidly growing and continues growing after the third treatment 10 days after the initiation of the treatment (Phase *III*). This behaviour continues after the forth treatment 15 days after the first treatment at Phase $IV_1$, such that the tumour has nearly vanished half a day after the forth infusion (Phase $IV_2$). Only a small region at the bottom of the tumour remains. However, exactly this is the problem. Without further medication, if medically meaningful, the tumour regrows and reaches at the end of the computation at $t_{{\mathrm{end}}}\approx 30.7$ days later the displayed condition.

Figure 20 exhibits numerical results obtained for different infusion scenarios. In particular, Fig. 20a, b exhibits the degradation and regrow of a brain metastasis under different TRAIL applications. Based on a single or a twice applied drug infusion, it is obvious that a higher drug concentration results in a stronger degradation of the tumour, although a regrow could not be avoided. In addition to this, Fig. 20c, d, presents multiply applied drug infusions with different TRAIL concentrations. The numerical studies presented in this chapter provide an option to plan a surgical treatment in advance, such that an optimal treatment with the most suited infusion protocol can be offered to the patient.

## Concluding remarks

In the present article, the computation of metastatic lung cancer cell proliferation and atrophy in brain tissue has been investigated based on experimental data that have been obtained by people around Professor Morrison at the University of Stuttgart. Based on these data, it was possible to calibrate a continuum mechanical model that has been elaborated on the basis of the Theory of Porous Media (TPM). Only by the use of the TPM, it was feasible to include a deforming solid skeleton, the porous solid brain structure, an interstitial fluid and blood as mutually acting constituents of the overall model. As living tissues need nutrients and further ingredients for their basal nutrition and growth, the interstitial fluid has been treated as a real mixture of a fluid solvent and various solutes or species, respectively. When cancer comes into play, a clustered cell structure nests in the skeleton tissue, here the brain, and starts to grow without accepting apoptosis signals.

The fact of growth, in particular proliferation and atrophy, has been included in the description by the introduction of a multiplicative decomposition of the solid deformation gradient in a mechanical and a growth-dependent part, on the one hand, and by the addition of a mass production term to the mass balance of the solid skeleton that interacts with the interstitial fluid, on the other hand. Based on the integration of production terms in the mass balance equations of solid and interstitial fluid, it could be shown that this procedure leads to the definition of a growth-dependent intermediate configuration that is different from the usual definitions in elasto-plasticity or in solid-based thermomechanics.

The constitutive equations of the overall model that had to be formulated for the complex description of solid deformation mutually coupled with flow processes of the interstitial fluid and the blood have been proven to fulfil the thermodynamical restrictions resulting from the entropy inequality of the multiphasic aggregate. In conclusion, the full set of constitutive equations including the fluid species that trigger cancer proliferation or apoptosis, the latter in the course of a medical treatment, is convenient for the computational description of the full and complex model under consideration.

Therewith, a model has been designed that can be used for pre-clinical studies to further validate and optimise model performance and to ultimately improve treatments and treatment outcomes to optimise the application of medical drugs, for example, in the skull.

## Figures and Tables

**Fig. 21:**
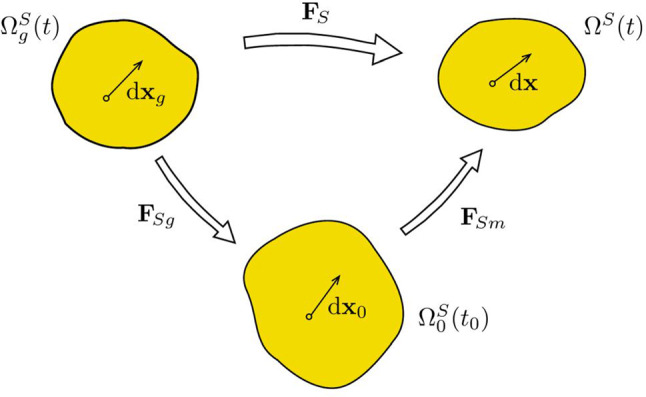
Line elements and corresponding configurations

## Appendix

The following relations make use of the tensor calculus, as it is explained in Ehlers (2018a) “Vector and Tensor Calculus—An Introduction”, release 2018, free download from https://www.mib.uni-stuttgart.de/en/institute/team/Ehlers-00001/.

### (a) Integration of the general mass balance

In porous media and mixture theories, the general mass balance of a species $\varphi ^\alpha$ reads

116 $$\begin{aligned} (\rho ^\alpha )^\prime _\alpha \,+\,\rho ^\alpha \,\hbox {div}\, \overset{\prime }{{{\mathbf{x}}}}_\alpha = {\hat{\rho }}{}^\alpha \,, \end{aligned}$$

cf. (4)$_1$. Integration of (116) needs a rewritten form of $\hbox {div}\, \overset{\prime }{{{\mathbf{x}}}}_\alpha$. This can be obtained by the use of the definition of the divergence operator yielding

117 $$\begin{aligned} \hbox {div}\, \overset{\prime }{{{\mathbf{x}}}}_\alpha&= \hbox {grad}\, \overset{\prime }{{{\mathbf{x}}}}_\alpha \,\cdot \,{\mathbf{I}} = ({\mathbf{F}}^T_\alpha )^{-1}\cdot \,(\mathbf{F}_\alpha )^{\prime }_\alpha \,, \end{aligned}$$

where $\hbox {grad}\,\overset{\prime }{{{\mathbf{x}}}}_\alpha =\mathbf{L}_\alpha =({\mathbf{F}}_\alpha )^{\prime }_\alpha ({\mathbf{F}}_\alpha )^{-1}$ has been used. As

118 $$\begin{aligned} ({\mathbf{F}}^T_\alpha )^{-1}=\frac{\hbox {cof}\,\mathbf{F}_\alpha }{\hbox {det}\,{\mathbf{F}}_\alpha } \end{aligned}$$

with $\hbox {cof}\,{\mathbf{F}}_\alpha$ as the cofactor of $\mathbf{F}_\alpha$, one obtains

119 $$\begin{aligned} \hbox {det}\,{\mathbf{F}}_\alpha \,\,\hbox {div}\, \overset{\prime }{{{\mathbf{x}}}}_\alpha= & {} \, \hbox {cof}\,\mathbf{F}_\alpha \cdot \,(\mathbf{F}_\alpha )^{\prime }_\alpha =\frac{\partial \hbox {det}\,\mathbf{F}_\alpha }{\partial {\mathbf{F}}_\alpha }\cdot \,(\mathbf{F}_\alpha )^{\prime }_\alpha \nonumber \\= & {} \, (\hbox {det}\,{\mathbf{F}}_\alpha )^\prime _\alpha \,. \end{aligned}$$

Therein, the derivative of $\hbox {det}\,{\mathbf{F}}_\alpha$ with respect to ${\mathbf{F}}_\alpha$ yielding $\hbox {cof}\,{\mathbf{F}}_\alpha$ has been taken into consideration. Rearranging (119) leads to

120 $$\begin{aligned} \hbox {div}\, \overset{\prime }{{{\mathbf{x}}}}_\alpha =\frac{(\hbox {det}\,\mathbf{F}_\alpha )^\prime _\alpha }{\hbox {det}\,{\mathbf{F}}_\alpha }\,. \end{aligned}$$

Based on (120), the mass balance (116) can be written as

121 $$\begin{aligned} \frac{(\rho ^\alpha )^\prime _\alpha }{\rho ^\alpha }\,+\frac{(\hbox {det}\,\mathbf{F}_\alpha )^\prime _\alpha }{\hbox {det}\,\mathbf{F}_\alpha } = \frac{{\hat{\rho }}{}^\alpha }{\rho ^\alpha }\,. \end{aligned}$$

With $(\,\cdot \,)^\prime _\alpha =\displaystyle \frac{\mathrm{d}(\,\cdot \,)}{{\mathrm{d}}t}$, the above equation yields

122 $$\begin{aligned} \frac{1}{\rho ^\alpha }\frac{\mathrm{d}\rho ^\alpha }{{{\mathrm{d}}}t}\,+\frac{1}{\hbox {det}\,{\mathbf{F}}_\alpha } \frac{{\mathrm{d}}\,(\hbox {det}\,{\mathbf{F}}_\alpha )}{\mathrm{d}t} = \frac{{\hat{\rho }}{}^\alpha }{\rho ^\alpha }\,. \end{aligned}$$

Multiplication of (122) with the time increment ${\mathrm{d}}t$ and integration between the terms of the initial configuration $\varOmega ^\alpha _0$ and the current configuration $\varOmega ^\alpha$ produces

123 $$\begin{aligned} \int ^{\rho ^\alpha }_{\rho ^\alpha _0}\frac{1}{\rho ^\alpha }\mathrm{d}\rho ^\alpha \,+\int ^{\mathrm {det}\,\mathbf{F}_\alpha }_{\mathrm {det}\,{\mathbf{F}}_{\alpha \,0}} \frac{1}{\hbox {det}\,{\mathbf{F}}_\alpha } {\mathrm{d}}\,(\hbox {det}\,\mathbf{F}_\alpha ) = \int ^t_{t_0}\frac{{\hat{\rho }}{}^\alpha }{\rho ^\alpha }{\mathrm{d}t}\,. \end{aligned}$$

As a result, one gets

124 $$\begin{aligned} \ln \rho ^\alpha -\ln \rho ^\alpha _0+\ln \,(\hbox {det}\,\mathbf{F}_\alpha )-\ln \,(\hbox {det}\,\mathbf{F}_{\alpha \,0}) = \int ^t_{t_0}\frac{{\hat{\rho }}{}^\alpha }{\rho ^\alpha }{\mathrm{d}t}\,. \end{aligned}$$

Proceeding from a natural reference configuration $\varOmega ^\alpha _0$, where ${\mathbf{F}}_{\alpha \,0}=\,{\mathbf{I}}$ and, as a result, $\hbox {det}\,{\mathbf{F}}_{\alpha \,0}=1$, such that (124) reads

125 $$\begin{aligned} \ln \bigg (\frac{\rho ^\alpha }{\rho ^\alpha _0}\bigg )+\ln \,(\hbox {det}\,\mathbf{F}_\alpha ) = \int ^t_{t_0}\frac{{\hat{\rho }}{}^\alpha }{\rho ^\alpha }{\mathrm{d}t}\,, \end{aligned}$$

one obtains

126 $$\begin{aligned} \ln \bigg (\frac{\rho ^\alpha }{\rho ^\alpha _0}\hbox {det}\,\mathbf{F}_\alpha \bigg ) = \int ^t_{t_0}\frac{{\hat{\rho }}{}^\alpha }{\rho ^\alpha }{\mathrm{d}t}\,. \end{aligned}$$

Applying the exponential function $\hbox {exp}\,(\,\cdot \,)$ to this equation, one obtains the integrated version of the general mass balance in the form

127 $$\begin{aligned} {\rho ^\alpha } = {\rho ^\alpha _0}\,\hbox {exp} \bigg (\int ^t_{t_0}\frac{{\hat{\rho }}{}^\alpha }{\rho ^\alpha }{\mathrm{d}t}\bigg )\,(\hbox {det}\,{\mathbf{F}}_\alpha )^{-1}\,. \end{aligned}$$

In case that no mass productions ${\hat{\rho }}^\alpha$ exist, (127) reduces to the standard form of the integrated mass balance reading

128 $$\begin{aligned} {\rho ^\alpha } = {\rho ^\alpha _0}\,(\hbox {det}\,\mathbf{F}_\alpha )^{-1}\,. \end{aligned}$$

Finally, if $\varphi ^\alpha$ can be considered as materially incompressible, such that $\rho ^{\alpha R}=\hbox {const.}$, (127) can also be written as

129 $$\begin{aligned} \begin{aligned} {n^\alpha }&= {n^\alpha _0}\,\hbox {exp} \bigg (\int ^t_{t_0}\frac{{\hat{\rho }}{}^\alpha }{\rho ^\alpha }{\mathrm{d}t}\bigg )\,(\hbox {det}\,{\mathbf{F}}_\alpha )^{-1}\\&= {n^\alpha _0}\,\hbox {exp} \bigg (\int ^t_{t_0}\frac{{\hat{n}}^\alpha }{n^\alpha }{\mathrm{d}t}\bigg )\,(\hbox {det}\,{\mathbf{F}}_\alpha )^{-1}\,, \end{aligned} \end{aligned}$$

where ${\hat{\rho }}^\alpha ={\hat{n}}^\alpha \rho ^{\alpha R}$ has been used.

### (b) Multiplicative decomposition of the deformation gradient ${\mathbf{F}}_S$

Based on a pioneering work by Lee (1969) on “elastic–plastic deformation at finite strains” and earlier work by Kröner (1955) and Bilby et al. (1957), the solid deformation gradient ${\mathbf{F}}_S$ can be split into an elastic and a plastic part:

130 $$\begin{aligned} {\mathbf{F}}_S={\mathbf{F}}_{Se}{\mathbf{F}}_{Sp}\,. \end{aligned}$$

This decomposition has been associated with a plastic intermediate configuration $\varOmega ^S_p$, where the plastic deformation is stored in the memory of the material.

Following the basic idea of (130), the concept of a multiplicative decomposition of the deformation gradient has also been applied in thermomechanics, cf. Lu and Pister (1975), where

131 $$\begin{aligned} {\mathbf{F}}_S={\mathbf{F}}_{Sm}{\mathbf{F}}_{S\theta } \end{aligned}$$

with ${\mathbf{F}}_{Sm}$ as the purely mechanical and ${\mathbf{F}}_{S\theta }$ as the purely thermal part of ${\mathbf{F}}_S$ has been introduced in combination with an isotropic a-priori constitutive equation for ${\mathbf{F}}_{S\theta }$ reading

132 $$\begin{aligned} {\mathbf{F}}_{S\theta }= & {} (\hbox {det}\,{\mathbf{F}}_{S\theta })^{1/3}\,\mathbf{I}~~~~\hbox {with}\nonumber \\ \hbox {det}\,\mathbf{F}_{S\theta }= & {} \text{ exp }(3\alpha ^S\,\varDelta \theta )\,. \end{aligned}$$

Therein, $\alpha ^S$ is the solid’s coefficient of thermal expansion and $\varDelta \theta =\theta -\theta _0$ the temperature variation compared to the initial temperature $\theta _0$.

When isotropic growth comes into play, as it is the case in the present article as a result of tumour growth, the above procedure can also be applied to growing material yielding

133 $$\begin{aligned}&{\mathbf{F}}_S={\mathbf{F}}_{Sm}{\mathbf{F}}_{Sg} ~~~\hbox {with}~~~ \mathbf{F}_{Sg}=(\hbox {det}\,{\mathbf{F}}_{Sg})^{1/3}\,{\mathbf{I}}\,,\nonumber \\&\hbox {such that}~~~{\mathbf{F}}_S=(\hbox {det}\,{\mathbf{F}}_{Sg})^{1/3}\,\mathbf{F}_{Sm}\,. \end{aligned}$$

As growing material is governed by the general mass balance, cf. for example (129), this gives rise to the a priori constitutive equation

134 $$\begin{aligned} \hbox {det}\,{\mathbf{F}}_{Sg}=\hbox {exp} \bigg (\int ^t_{t_0}\frac{{\hat{n}}^S}{n^S}{{\mathrm{d}}t}\bigg )=\hbox {exp} \bigg (\int ^t_{t_0}\frac{{\hat{\rho }}^S}{\rho ^S}{{\mathrm{d}}t}\bigg )\,, \end{aligned}$$

such that

135 $$\begin{aligned} {n^S} = {n^S_0}\,\hbox {det}\,{\mathbf{F}}_{Sg}\,(\hbox {det}\,\mathbf{F}_S)^{-1}=\,{n^S_0}\,(\hbox {det}\,{\mathbf{F}}_{Sm})^{-1}\,, \end{aligned}$$

where $\hbox {det}\,{\mathbf{F}}_S=\hbox {det}\,\mathbf{F}_{Sm}\,\hbox {det}\,{\mathbf{F}}_{Sg}$ has been used, thus furthermore substituting (128) in case of materially incompressible $\varphi ^S$. From (135), it is seen that $(\hbox {det}\,{\mathbf{F}}_{Sm})^{-1}$ plays the role of a push-forward transformation of $n^S_0$ belonging to the reference configuration $\varOmega ^S_0$ towards $n^S$ belonging to the current configuration $\varOmega ^S$. From (135), we additionally have

136 $$\begin{aligned} n^S\,\hbox {det}\,{\mathbf{F}}_S=n^S_0\,\hbox {det}\,\mathbf{F}_{Sg}\,, \end{aligned}$$

where both, $\hbox {det}\,{\mathbf{F}}_S$ and $\hbox {det}\,{\mathbf{F}}_{Sg}$, are pull-back transformations of volume fractions pulling $n^S$ from $\varOmega ^S$ and $n^S_0$ from $\varOmega ^S_0$ towards $n^S_g$ of the same configuration $\varOmega ^S_g$. As a result,

137 $$\begin{aligned} n^S_g=n^S\,\hbox {det}\,\mathbf{F}_S=n^S_0\,\hbox {det}\,{\mathbf{F}}_{Sg}\,, ~~\hbox {while}~~~ n^S_0=n^S\,\hbox {det}\,{\mathbf{F}}_{Sm}\,. \end{aligned}$$

Therein, $\hbox {det}\,{\mathbf{F}}_S$, $\hbox {det}\,{\mathbf{F}}_{Sm}$ and $\hbox {det}\,{\mathbf{F}}_{Sg}$ do not only define pull-back transformations of volume fractions but also push-forward transformations of volume elements yielding

138 $$\begin{aligned} {\mathrm{d}}v=\hbox {det}\,{\mathbf{F}}_S\,{\mathrm{d}}v_g\,,~~ {\mathrm{d}}v_0=\hbox {det}\,{\mathbf{F}}_{Sg}\,{\mathrm{d}}v_g\,,~~ \mathrm{d}v=\hbox {det}\,{\mathbf{F}}_{Sm}\,{\mathrm{d}}v_0\,, \end{aligned}$$

where the corresponding deformation gradients ${\mathbf{F}}_S$, $\mathbf{F}_{Sg}$ and ${\mathbf{F}}_{Sm}$ transform line elements via

139 $$\begin{aligned}&{\mathrm{d}}{\mathbf{x}}={\mathbf{F}}_S\,{\mathrm{d}}{\mathbf{x}}_g\,,~~ {\mathrm{d}}\mathbf{x}_0={\mathbf{F}}_{Sg}\,{\mathrm{d}}{\mathbf{x}}_g\,,~~ {\mathrm{d}}{\mathbf{x}}=\mathbf{F}_{Sm}\,{\mathrm{d}}{\mathbf{x}}_0\nonumber \\& \hbox {resulting in}~~{\mathrm{d}}{\mathbf{x}}={\mathbf{F}}_{Sm}{\mathbf{F}}_{Sg}\,\mathrm{d}{\mathbf{x}}_g\,. \end{aligned}$$

By these means, one obtains the order of configurations as in Fig. 21 differing from the standard literature, cf. Sect. 3.3.

### (c) Nonlinear elasticity of growing brain tissue

As in thermoelasticity, the purely mechanical deformation-versus-stress behaviour is governed by ${\mathbf{F}}_{Sm}$ also defining the right Cauchy–Green tensor ${\mathbf{C}}_{Sm}$, cf. (77)$_1$, while the growth behaviour depends on (133). Based on this equation, the growth-depending Cauchy–Green strain yields

140 $$\begin{aligned} {\mathbf{C}}_{Sg}={\mathbf{F}}_{Sg}^T\mathbf{F}_{Sg}=(\hbox {det}\,{\mathbf{F}}_{Sg})^{2/3}\,{\mathbf{I}}\,. \end{aligned}$$

As ${\mathbf{C}}_S$, ${\mathbf{C}}_{Sm}$ and ${\mathbf{C}}_{Sg}$ are coupled to each other through the multiplicative decomposition (133), only two of them are independent, while the third is a function of these two. In terms of the exploitation of the entropy inequality of the whole system, cf. (33), it is convenient to proceed with ${\mathbf{C}}_S={\mathbf{F}}^T_S{\mathbf{F}}_S$ and ${\mathbf{C}}_{Sg}$, where $\mathbf{C}_S$ is directly coupled to the solid motion trough the solid displacement ${\mathbf{u}}_S$ and the deformation gradient ${\mathbf{F}}_S$, while ${\mathbf{C}}_{Sg}$ is a function of growth through ${\hat{n}}^S$ or ${\hat{\rho }}^S$, respectively, cf. (134).

From the entropy inequality, one has (41) reading

141 $$\begin{aligned} {\mathbf{T}}^S=-n^S p^{FR}\,{\mathbf{I}}\,+\mathbf{T}^S_{{\mathrm{eff}}} \quad \text{ with }\quad \mathbf{T}^S_{{\mathrm{eff}}}=\rho ^S\frac{\partial \psi ^S}{\partial \mathbf{F}_S}{\mathbf{F}}^T_S\,, \end{aligned}$$

where, by means of the principle of determinism, $\psi ^S$ was assumed as a function of ${\mathbf{F}}_S$. Based on the principle of frame indifference, the dependence of $\psi ^S$ on ${\mathbf{F}}_S$ must be found in such a way that $\psi ^S$ depends on ${\mathbf{F}}_S$ through ${\mathbf{C}}_S$. This results in (78). To obtain (78) from (134), we use the following procedure. Starting with the chain rule, one obtains

142 $$\begin{aligned} \frac{\partial \psi ^S}{\partial \mathbf{F}_S}=\left( \frac{\partial {\mathbf{C}}_S}{\partial \mathbf{F}_S}\right) {}^T\frac{\partial \psi ^S}{\partial {\mathbf{C}}_S}\,, \end{aligned}$$

where

143 $$\begin{aligned} \frac{\partial {\mathbf{C}}_S}{\partial {\mathbf{F}}_S}=(\mathbf{F}^T_S\otimes \,{\mathbf{I}})^{\overset{23}{T}}+({\mathbf{I}}\,\otimes \mathbf{F}_S)^{\overset{24}{T}} \end{aligned}$$

and

144 $$\begin{aligned} \bigg (\frac{\partial {\mathbf{C}}_S}{\partial \mathbf{F}_S}\bigg ){}^T=({\mathbf{F}}_S\otimes \,{\mathbf{I}})^{\overset{23}{T}}+(\mathbf{F}_S\,\otimes {\mathbf{I}})^{\overset{24}{T}} \end{aligned}$$

result in

145 $$\begin{aligned} \frac{\partial \psi ^S}{\partial {\mathbf{F}}_S}= & {} \bigg [(\mathbf{F}_S\otimes \,{\mathbf{I}})^{\overset{23}{T}}+({\mathbf{F}}_S\,\otimes \mathbf{I})^{\overset{24}{T}}\bigg ]\frac{\partial \psi ^S}{\partial {\mathbf{C}}_S}\nonumber \\= & {} {\mathbf{F}}_S\frac{\partial \psi ^S}{\partial {\mathbf{C}}_S}+\mathbf{F}_S\left( \frac{\partial \psi ^S}{\partial {\mathbf{C}}_S}\right) {} ^T =2\,\mathbf{F}_S\frac{\partial \psi ^S}{\partial {\mathbf{C}}_S}\,, \end{aligned}$$

where the symmetry of ${\mathbf{C}}_S$ has been used. Thus, combining (141)$_2$ with (145) by the use of $W^S=\rho ^S_0\psi ^S$ and $n^S/n^S_0=\rho ^S/\rho ^S_0=\hbox {det}\,\mathbf{F}_{Sm}$, the effective stress reads

146 $$\begin{aligned} {\mathbf{T}}^S_{{\mathrm{eff}}}=2\,(\text{ det }\,\mathbf{F}_{Sm})^{-1}{\mathbf{F}}_S\frac{\partial W^S}{\partial {\mathbf{C}}_S}{} \mathbf{F}^T_S\,. \end{aligned}$$

Given the stored energy $W^S$ after (79) as a function of ${\mathbf{C}}_{Sm}$ through $I_m={\mathbf{C}}_{Sm}\cdot \,{\mathbf{I}}$ and $J_{Sm}=\hbox {det}\,{\mathbf{F}}_{Sm}$, one needs the derivative of $W^S$ with respect to ${\mathbf{C}}_S$. Thus,

147 $$\begin{aligned}&\frac{\partial W^S}{\partial {\mathbf{C}}_S}=\bigg (\frac{\partial \mathbf{C}_{Sm}}{\partial {\mathbf{C}}_S}\bigg ){}^T\,\frac{\partial W^S}{\partial {\mathbf{C}}_{Sm}} \end{aligned}$$

needs to be computed. From (77)$_1$, one concludes to

148 $$\begin{aligned}&{\mathbf{C}}_{Sm}={\mathbf{F}}^{T-1}_{Sg}{\mathbf{C}}_S\,\mathbf{F}^{-1}_{Sg}\,,~~~\hbox {such that}\nonumber \\&\frac{\partial {\mathbf{C}}_{Sm}}{\partial {\mathbf{C}}_S}=(\mathbf{F}^{T-1}_{Sg}\otimes \mathbf{F}^{T-1}_{Sg})^{\overset{23}{T}}~~~\hbox {and}\nonumber \\&\bigg (\frac{\partial {\mathbf{C}}_{Sm}}{\partial \mathbf{C}_S}\bigg ){}^T=({\mathbf{F}}^{-1}_{Sg}\otimes \mathbf{F}^{-1}_{Sg})^{\overset{23}{T}}\,. \end{aligned}$$

Combining (147) and (148) yields

149 $$\begin{aligned} \frac{\partial W^S}{\partial \mathbf{C}_{S}}=\bigg (\frac{\partial {\mathbf{C}}_{Sm}}{\partial \mathbf{C}_{S}}\bigg ){}^T\frac{\partial W^S}{\partial {\mathbf{C}}_{Sm}}=\mathbf{F}^{-1}_{Sg}\frac{\partial W^S}{\partial {\mathbf{C}}_{Sm}}{} \mathbf{F}^{T-1}_{Sg}\,. \end{aligned}$$

A further combination of (146) and (149) finally yields

150 $$\begin{aligned} {\mathbf{T}}^S_{{\mathrm{eff}}}=2\,(\text{ det }\,\mathbf{F}_{Sm})^{-1}{\mathbf{F}}_{Sm}\frac{\partial W^S}{\partial \mathbf{C}_{Sm}}{\mathbf{F}}^T_{Sm}\,. \end{aligned}$$

Based on this equation and the stored energy $W^S$ after (79), the finite elasticity law for growing material can be found. Building

151 $$\begin{aligned} \frac{\partial W^S}{\partial \mathbf{C}_{Sm}}= & {} {\textstyle \frac{1}{2}}\mu ^S_0\,\,\mathbf{I}\,-\mu ^S_0J^{-1}_m\frac{\partial J_{Sm}}{\partial {\mathbf{C}}_{Sm}}\nonumber \\&+\,\lambda ^S_0(1-n^S_0)^2\bigg (\frac{1}{1-n^S_0}-\frac{1}{J_{Sm}-n^S_0} \bigg )\frac{\partial J_{Sm}}{\partial {\mathbf{C}}_{Sm}}\,, \end{aligned}$$

where

152 $$\begin{aligned} \frac{\partial J_{Sm}}{\partial {\mathbf{C}}_{Sm}}= & {} \frac{\partial (III_m)^{1/2}}{\partial III_m}\frac{\partial III_m}{\partial \mathbf{C}_{Sm}}\nonumber \\= & {} {\textstyle \frac{1}{2}}J^{-1}_m\hbox {cof}\,{\mathbf{C}}_{Sm} ={\textstyle \frac{1}{2}}J_m{\mathbf{C}}^{-1}_{Sm} \end{aligned}$$

and $III_m=\hbox {det}\,{\mathbf{C}}_{Sm}=J^2_m$ have been used, the effective solid stress reads

153 $$\begin{aligned} {\mathbf{T}}^S_{{\mathrm{eff}}}=J_{Sm}^{-1}\,\Bigg [\,2\,\mu ^S_0\,\mathbf{K}_{Sm}+\lambda ^S_0(1-n^S_0)^2\Bigg (\frac{J_{Sm}}{1-n^S_0} -\frac{J_{Sm}}{J_{Sm}-n^S_0}\Bigg )\,\mathbf{I}\,\Bigg ] \end{aligned}$$

with ${\mathbf{K}}_{Sm}=\frac{1}{2}({\mathbf{B}}_{Sm}-\,{\mathbf{I}}\,)$ as the mechanical part of the Karni–Reiner strain, cf. (80).

## Footnotes

1 **P**orous media** a**daptive** n**onlinear finite-element solver based on** d**ifferential **a**lgebraic** s**ystems (http://www.get-pandas.com).

2 TRAIL:** T**umour-necrosis-factor-**R**elated** A**poptosis** I**nducing** L**igand.

3 Matlab Version R 2017 b (9.3.0.713579).
